# A medical imaging analysis system for trigger finger using an adaptive texture-based active shape model (ATASM) in ultrasound images

**DOI:** 10.1371/journal.pone.0187042

**Published:** 2017-10-27

**Authors:** Bo-I Chuang, Li-Chieh Kuo, Tai-Hua Yang, Fong-Chin Su, I-Ming Jou, Wei-Jr Lin, Yung-Nien Sun

**Affiliations:** 1 Department of Computer Science and Information Engineering, National Cheng Kung University, Tainan, Taiwan; 2 Department of Occupational Therapy, National Cheng Kung University, Tainan, Taiwan; 3 Department of Biomedical Engineering, National Cheng Kung University, Tainan, Taiwan; 4 Department of Orthopedics, E-Da Hospital, Kaohsiung, Taiwan; Beijing University of Technology, CHINA

## Abstract

Trigger finger has become a prevalent disease that greatly affects occupational activity and daily life. Ultrasound imaging is commonly used for the clinical diagnosis of trigger finger severity. Due to image property variations, traditional methods cannot effectively segment the finger joint’s tendon structure. In this study, an adaptive texture-based active shape model method is used for segmenting the tendon and synovial sheath. Adapted weights are applied in the segmentation process to adjust the contribution of energy terms depending on image characteristics at different positions. The pathology is then determined according to the wavelet and co-occurrence texture features of the segmented tendon area. In the experiments, the segmentation results have fewer errors, with respect to the ground truth, than contours drawn by regular users. The mean values of the absolute segmentation difference of the tendon and synovial sheath are 3.14 and 4.54 pixels, respectively. The average accuracy of pathological determination is 87.14%. The segmentation results are all acceptable in data of both clear and fuzzy boundary cases in 74 images. And the symptom classifications of 42 cases are also a good reference for diagnosis according to the expert clinicians’ opinions.

## 1. Introduction

Trigger finger, which is usually caused by repetitive or forceful use of the finger, has become a frequent occupational disease in recent years. It occurs when a nodule is formed in the tendon, causing a mismatch of the volumes of the tendon and pulley. The tendon may become stuck at the mouth of the tendon sheath tunnel so that flexion and extension become ragged [1]. Treatment depends on severity. Corticosteroid injections can cure moderate cases. However, only surgery can resolve the symptoms at later stages.

**Fig 1:**
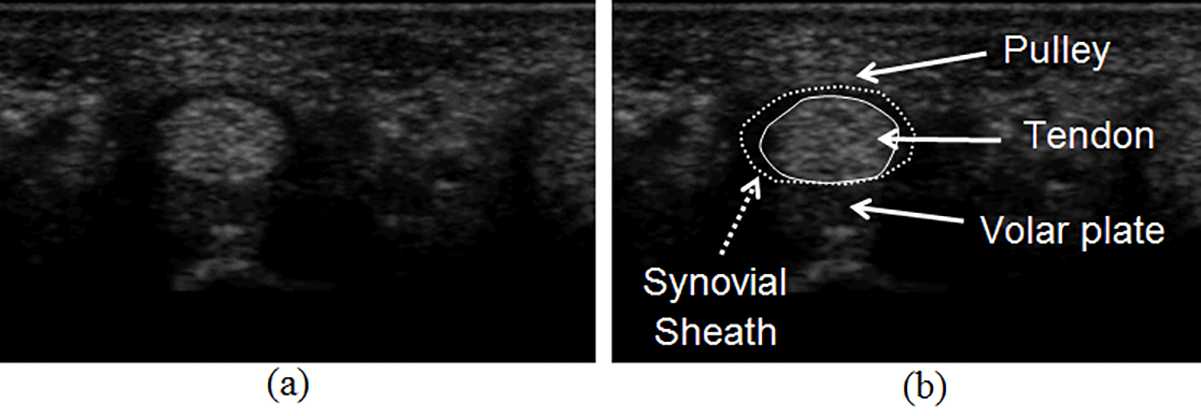
Tendon and synovial sheath in the finger ultrasound image. (a) acquired original image; (b) tendon (solid line), synovial sheath (broken line) area and surrounding tissues.

In clinical settings, ultrasound imaging is used most in trigger finger diagnosis and surgery. Fig 1(A) shows an ultrasound tendon image acquired transversely at the first annular (A1) pulley position, and Fig 1(B) indicates the tendon and surrounding tissues. Pathological changes alter the tissue’s characteristics when it becomes irritated. Some research demonstrated that the A1 pulley or tendon becomes thick and hypoechoic. However, these studies segmented the tendon and pulley manually. Manual assessments may yield inconsistent results due to intra- and inter-observer deviations [2–4]. An image analysis system that automatically segments the tendon and synovial sheath may address these limitations.

**Fig 2:**
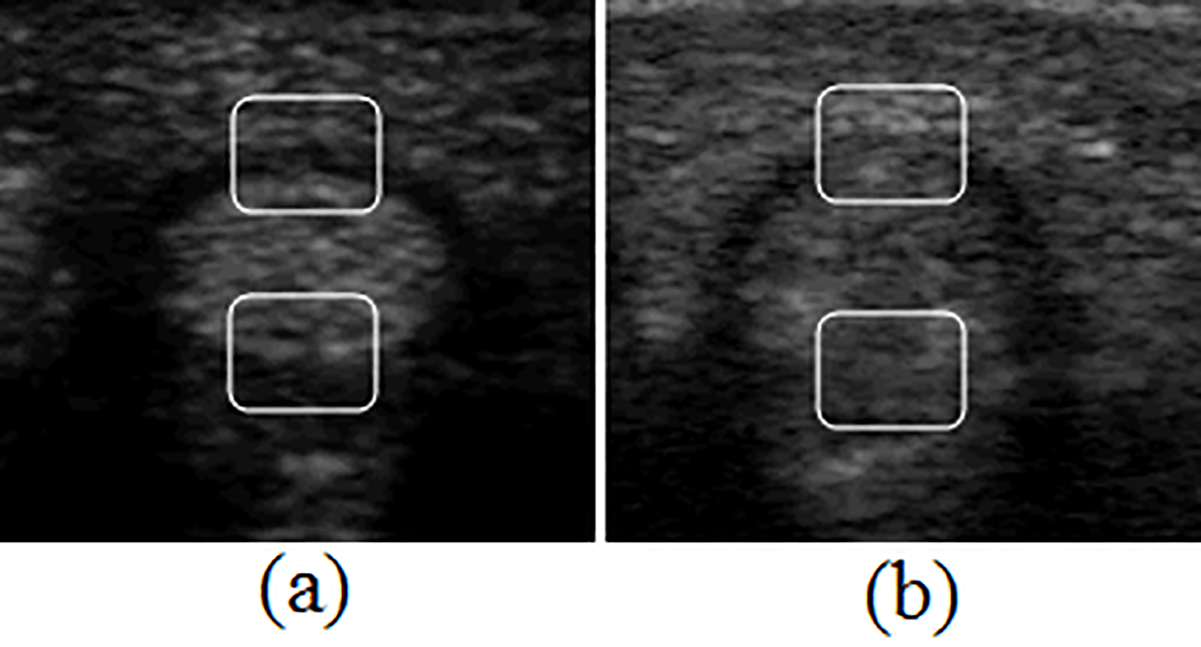
Different characteristics of edge at the synovial sheath (top region) and the volar plate (bottom region). (a) better image quality; (b) worse image quality.

As shown in Fig 1(B), the tendon is elliptical. The synovial sheath surrounds it, and the volar plate is attached underneath. The underlying tissues and the acquisition position and angle affect an ultrasound image’s quality. The synovial sheath’s boundary exhibits good contrast at the top region of Fig 2(A), while it is fuzzy in Fig 2(B). Similarly, the tendon’s lower boundaries appear different in Fig 2(A) and 2(B) because the volar plates echo at a wide range of intensities. Such variations may make it difficult to segment the tendon and sheath using only intensity-based clues. However, shape and local texture measures, such as the Gabor texture [5], are commonly used to increase the accuracy of the segmentation process. Several research groups have introduced shape-based methods, such as the active contour model (ACM) and active shape model (ASM), for tissue segmentation in ultrasound images [6–15]. Hamameh and Gustavsson [6] presented a method that combined the ASM with an ACM to clarify the boundary of the left ventricle in echocardiograms. Tsai et al. [11] and Chen et al. [12] proposed an automatic image analysis system that precisely measures the fetal craniofacial structures using a surface template model.

Many of these studies segmented tissue by applying models to sonograms. However, model-based methods cannot always overcome the noise and deviations in images that prevent accurate segmentation. To increase reliability, many studies incorporated local texture features into model-based methods [16–24]. Kim et al. [16] and Huang and Dony [17] used frequency-based texture features for tendon segmentation in equine legs. Christodoulou et al. [18] classified both symptomatic and asymptomatic atherosclerotic carotid plaques using 61 texture features and a self-organized map classifier. In these studies, using the texture features provided good results.

In this paper, we propose a new model-based method called the adaptive texture-based active shape model (ATASM) to segment tendons and synovial sheaths in clinical ultrasound images. We combine the model-based method with the use of texture features to segment the tendon and synovial sheath at the A1 pulley position from the ultrasound image. A new weighting mechanism is designed to adjust the contributions of different features in the objective function. To validate the proposed method, 58 finger ultrasound images are acquired and analyzed. In addition, we adopt the SVM classifier, using tissue size and textural features extracted from the segmented area to classify tendons as symptomatic or asymptomatic.

## 2. Materials and methods

### 2.1. Ultrasound data acquisition and ethics

The ultrasound image data are acquired from National Cheng Kung University Hospital and Ton-Yen General Hospital using MicroMaxx portable ultrasound system (Sonosite, Bothell, WA, USA) and t3000 ultrasound system (Terason, Burlington, MA, USA) with a 13MHz probe. Prior to participation, all participants were informed about the study’s aims and procedures and signed consent forms approved by the Institutional Review Board of National Cheng Kung University Hospital and Ton-Yen General Hospital (IRB number: HR-98-048). Each participant laid the hand on the table palm up for ultrasound acquisition. In all images, each pixel is 0.075x0.075mm^2^. To evaluate the characteristic differences between symptomatic (trigger finger) and asymptomatic samples, 21 images of each hand are acquired from both patients and normal individuals.

### 2.2. ATASM overview

**Fig 3:**
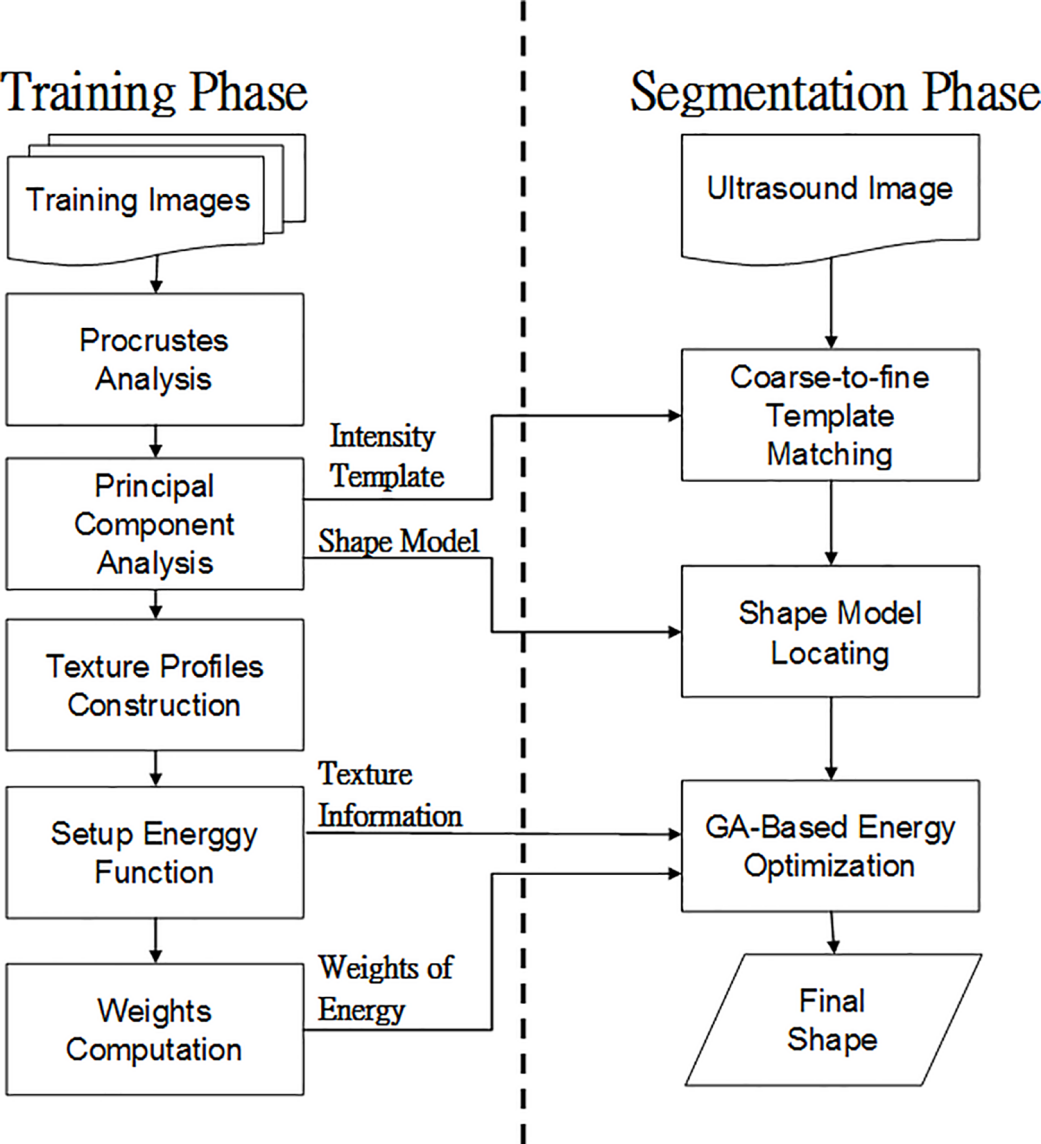
ATASM Flowchart.

Fig 3 shows the ATASM procedure, which included a training phase and segmentation phase. In the training phase, the model is constructed with several manually drawn contours, which are first aligned using a Procrustes analysis [25]. A principal component analysis (PCA) [26] is then applied to construct the point distribution model:

$$X=\bar{X}+\mathrm{Pb},$$ (1)

where $\bar{X}$ is the average shape, ***P*** is the set of eigenvectors of shape variability, and ***b*** is the set of tunable shape parameters that control the weight of the eigenvectors. Tuning the parameters in ***b*** can adjust the shape to fit different targets. To prevent large deformation, each parameter must satisfy the following constraint:

$$-3\sqrt{\lambda_{i}}\leq b_{i}\leq 3\sqrt{\lambda_{i}},$$ (2)

where *λ*_*i*_ is the *i*th eigenvalue of the point distribution model.

In the segmentation phase, the point distribution model is modified by iteratively adjusting the pose parameters, including translation, scaling, and rotation, and then the shape parameters in ***b***. Tendon images usually have poor quality and complex boundary characteristics. Therefore, to overcome complex tendon segmentation problems, we modify the ASM principle to include texture features as one of the energy terms and adopt the adaptive weighting mechanism. A genetic algorithm (GA, [27]) is then applied to optimize the final shape parameters by maximizing the objective function, which includes texture and gradient energies.

### 2.3. Shape model construction

**Fig 4:**
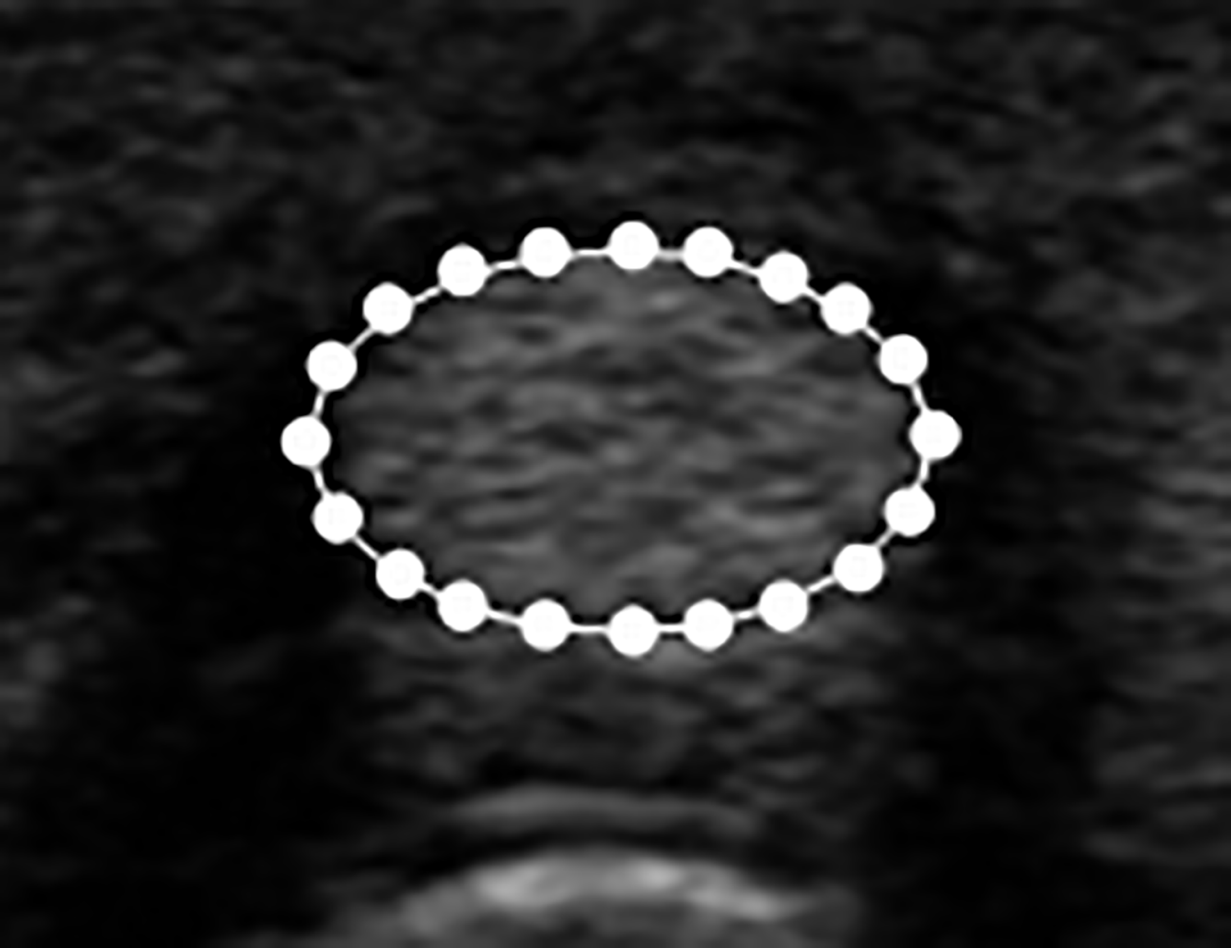
Landmark points of the training data.

In the training phase, the contour correspondence among the different training images is established. Contour correspondences are sampled as a set of control points according to the objects’ characteristic, such as a corner or cross point. Tendon and synovial sheath contours drawn by an expert are used for training the point distribution model separately. Because both the tendon and synovial sheath have elliptical shapes, the points at the top, bottom, left, and right are selected as control points. Four points between every two adjacent control points are evenly resampled to form a contour with 20 control points on the boundary, as shown in Fig 4. The control points on each training contour are then aligned using the Procrustes analysis [25]. After the alignment process, the PCA is applied to construct the point distribution model. The obtained mean shape $\bar{X}$ and the eigenvector ***P*** are then used in Eq (1) to fit the image in the segmentation process.

### 2.4. Texture profile construction

The model is deformed by maximizing the energy function, which includes curvature, gradient, and texture information. Thus, texture profiles that contain the texture information from the training data are needed. Considering the textural characteristics of the tendon boundary, some of the Gabor [5] and Laws’ texture features [28] are also used in the energy function. The Gabor texture is a general texture descriptor that comprises a real component and an imaginary component representing the orthogonal directions. Both real and imaginary components are extracted using the real and imaginary parts of the Gabor filtered results:

$$G_{R}\left(x,y\right)=\frac{1}{2\pi \sigma^{2}}\cdot \exp\left(-\frac{x^{2}+y^{2}}{2\sigma^{2}}\right)\cdot \cos\left[2\pi \phi \left(x\cos\theta +y\sin\theta \right)\right];$$ (3)

$$G_{I}\left(x,y\right)=\frac{1}{2\pi \sigma^{2}}\cdot \exp\left(-\frac{x^{2}+y^{2}}{2\sigma^{2}}\right)\cdot \sin\left[2\pi \phi \left(x\cos\theta +y\sin\theta \right)\right].$$ (4)

**Fig 5:**
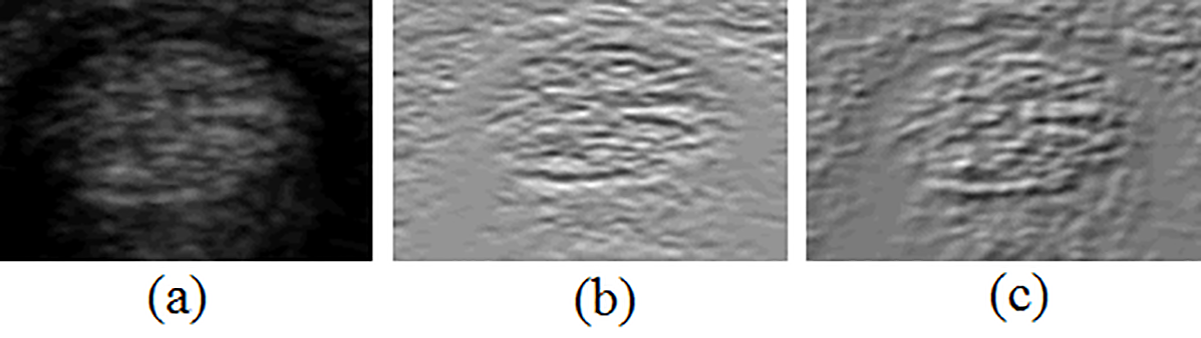
Texture images with different settings. (a) original image; (b) imaginary section of Gabor with 0 degrees; (c) level-edge part of Laws’ texture energy measure images.

The real and imaginary components of the Gabor images are extracted using Gabor filters with angles of 0°, 30°, 60°, and 90°. The tendon contours are longer and stabler on the upper and lower boundaries. The imaginary components of 0°in the Gabor images shows clear tendon boundary on the upper and lower parts, as shown in Fig 5(B), and thus is adopted as one of the texture features.

The Laws’ texture energy measure is another famous texture feature and includes three types of masks: local averaging, an edge detector, and a spot detector. These are applied in both directions to obtain the texture images. Six texture features are extracted using six different 5x5-pixel square kernels. The tendon boundary using the level-edge kernel in Eq (5) has the most significant edge and is adopted as one of the texture features, as shown in Fig 5(C).

$$\left[\begin{array}{ccccc} -1 & -4 & -6 & -4 & -1\\ -2 & -8 & -12 & -8 & -2\\ 0 & 0 & 0 & 0 & 0\\ 2 & 8 & 12 & 8 & 2\\ 1 & 4 & 6 & 4 & 1 \end{array}\right].$$ (5)

The texture features are computed on each control point and its four related points, two inside and two outside the contour every two pixels on the search line perpendicular to the current contour. Because we has 20 control points, each of which forms a 1x5 texture vector, we can concatenate the vectors and obtain a 1x100 vector of 100 texture values as the texture profile for each texture feature from each training image. The texture model is then constructed by averaging the texture profiles from all of the training images.

### 2.5. Energy function setup

In the original ASM, the gradient value along the search line at each control point is defined as the energy function. In tendon images, the gradient provides boundary information because of the high contrast between the hyperechoic tendon area and the hypoechoic area surrounding the synovial fluid. However, the gradient information is not adequate to find the tendon contour in complex ultrasound images. Clinicians also rely on texture. We thus adopt the Gabor and Laws’ texture measures as the features for the energy function in the ATASM. Because the tendon contour is usually smooth, the curvature of the control points is considered another feature of the energy function. In the ATASM, the energy function of the model deformation is maximized and defined as

$$F=\sum_{k}\sum_{i}w_{i}^{k}F_{i}^{k},$$ (6)

where $w_{i}^{k}$ and $F_{i}^{k}$ are the weight and the value of *k*th energy term on the *i*th control point, respectively. Due to the different image characteristics of the tendon and synovial sheath, the energy terms are defined distinctively and are described in later sections.

### 2.6. Weight of energy term computation

The weights of the energy terms vary at different contour positions depending on the corresponding image characteristics. When computing the total ATASM energy, the most effective energy is assigned the highest weight. The larger energy value and larger standard deviation for a given energy term at a control point implies the energy term and position are more effective in ASM model modification. Thus, energy value and standard deviation are used to compute the weights. Along the search line, if the energy value on the correct tendon boundary is larger than on the other points and the standard deviation of the energy values is large, the energy term is considered effective in discerning the control point’s position. Based on this concept, the weights of energy terms are assigned by the following equation:

$$w_{i}^{k}=\frac{1}{T}\sum_{t=1}^{T}\sigma_{t,i}^{k}*\frac{F_{t,i}^{k}}{{\tilde{F}}_{t,i}^{k}},$$ (7)

where $F_{t,i}^{k}$, ${\tilde{F}}_{t,i}^{k}$, and $\sigma_{t,i}^{k}$ are the value, maximum value, and standard deviation, respectively, of the *k*th energy term at the *i*th control point across the search line in the *t*th training image and *T* is the total number of training images.

### 2.7. Shape model locating

In the ATASM, the shape model requires an initial location for the subsequent procedure. Due to the different characteristics of the tendon and synovial sheath, we employ different locating methods for the two types of tissue. In tendon segmentation, a coarse-to-fine matching mechanism is applied to locate the contour’s position automatically. In the synovial sheath segmentation, the contour is localized by the segmented tendon instead. The locating details are described in the next section.

### 2.8. GA-based energy optimization

Due to the noisy nature of ultrasound images, deformation may be trapped in many local optima. To obtain a near optimal tendon shape and prevent any deformation from being trapped in the local optima, we use the GA to search for the best shape parameter ***b***.

GA-based energy optimization is an efficient method by which to search for the optimal solution in a complex search domain. The chromosomes are initialized with a random generator and then evolve iteratively to produce the global solution. GA’s evolution processes include reproduction, crossover, and mutation. In reproduction, the chromosomes with better fitness values are copied to the next generation. In crossover, the partial parameters in a couple of chromosomes are exchanged to produce a new chromosome. In mutation, a new parameter is randomly generated and substitutes for the old one in a chromosome.

In this research, every chromosome represents a set of shape parameters in ***b***. The chromosomes of the initial population are first generated with a random generator, and each parameter *b*_*i*_ of ***b*** is required to satisfy the constraint of point distribution model in Eq (2). In addition, the fitness function is designed as the energy function of the model deformation in Eq (6). In the GA reproduction step, roulette wheel selection is used to select the chromosomes with better fitness values. The single point crossover and mutation with probabilities of 50% and 1%, respectively, are used in each generation. The mutation process replaces a random shape parameter *b*_*i*_ with a random value that satisfies the shape constraint in Eq (2). Elitism is applied to keep the best chromosome during evolution. If the best chromosome does not change for 10 generations, the evolution converges, and the elite chromosome is determined to be the best solution for ***b***.

### 2.9. ATASM for tendon segmentation

**Fig 6:**
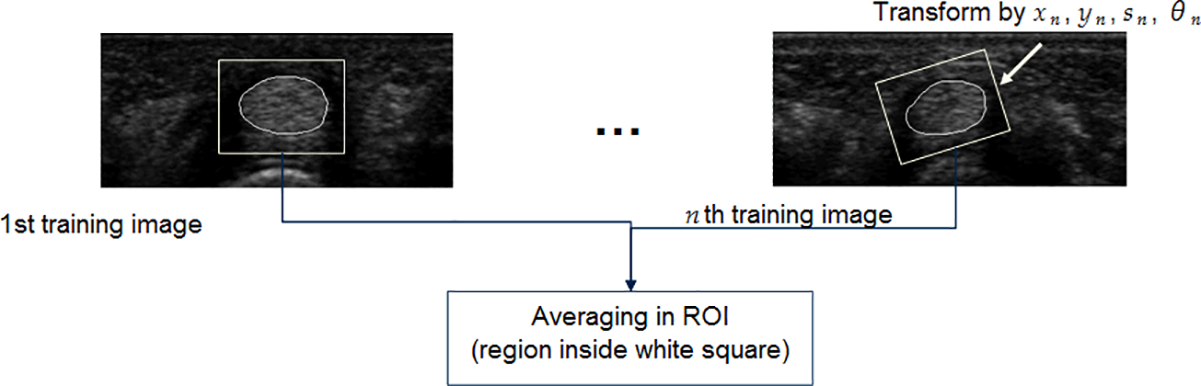
Procedure for intensity template training.

In tendon segmentation, an intensity template has to be obtained and used to search for the tendon on the test image. From a given set of training images, a reference template covering the tendon area is first selected from a proper training image. Then, the obtained contours from all the other training images are aligned to the reference tendon contour by Procrustes analysis. The final intensity template is obtained by averaging the intensities of all the aligned training images, as shown in Fig 6. The resulting intensity template cam then be used to locate the tendon area on the test ultrasound image, and the corresponding tendon contour can be used as the initial ATASM contour.

In the model localization step, a coarse-to-fine matching mechanic is applied to initialize the contour position automatically. In the coarse matching, we first reduce the image resolution by two to increase the search speed. The input image is then split into several overlapping blocks. For each block, the sum of the absolute difference (SAD) value with respect to the intensity template is computed:

$$SAD=\frac{1}{N}\sum_{\left(x,y\right)}\left|A\left(x,y\right)-B\left(x,y\right)\right|,$$ (8)

where A(x, y) and B(x, y) are the intensity values of a single block and intensity template at point (x, y) and *N* is the size of the template. The block with the smallest SAD value is selected to refine the initial position during fine matching. In the fine matching step, the transformation parameters of the minimum SAD value between the transformed intensity template and the selected block in the original image resolution are computed. The parameters are optimized with Powell’s method.

To set up the energy function, the segmentation target’s image characteristics are considered. Because the tendon boundary is usually smooth, the curvature at each control point on the contour is considered small. The internal energy term, which is the curvature value, is defined as

$$F_{i}^{Curv}=\mathrm{arc}\;\cos\left(\frac{\left(c\left(i+1\right)-c\left(i\right)\right)\cdot \left(c\left(i-1\right)-c\left(i\right)\right)}{\left|c\left(i+1\right)-c\left(i\right)\right|\left|c\left(i-1\right)-c\left(i\right)\right|}\right),$$ (9)

where *c*(*x*) is the position of control point *x* and the dot operator indicates the dot product of two vectors. The gradient properties that are bright inside and dark outside across the tendon boundary are used as two image energy terms for segmentation. Two types of energy terms, area-based gradient $F_{i}^{Area\_grad}$ and line-based gradient $F_{i}^{Line\_grad}$, are defined as

$$F_{i}^{Area\_grad}={\bar{I}}_{i}^{inner\_area}-{\bar{I}}_{i}^{outer\_area};$$ (10)

$$F_{i}^{Line\_grad}={\bar{I}}_{i}^{inner\_line}-{\bar{I}}_{i}^{outer\_line},$$ (11)

where ${\bar{I}}_{i}^{inner\_area}$ and ${\bar{I}}_{i}^{outer\_area}$ are the inside and outside 3x3-pixel rectangular areas on the perpendicular search line across the *i*th control points and ${\bar{I}}_{i}^{inner\_line}$ and ${\bar{I}}_{i}^{outer\_line}$ are the two opposite linear areas on the perpendicular search line across the *i*th control points. The area-based gradient using a rectangular window can reduce the noise effect when computing the gradient. Because the tendon area is light in intensity and the surrounding synovial fluid is dark and has a specific width, the line-based gradient is thus designed with a mask of 5x1 pixels. Both the Gabor and Laws’ texture energy terms are defined as

$$\mathrm{Gabor}:\;F^{G}=-\left(\sum_{i}{\left(G_{i}^{M,0{}^{\circ}}-G_{i}^{T,0{}^{\circ}}\right)}^{2}\right);$$ (12)

$$\mathrm{Laws}’:\;F^{L}=-\sum_{i}{\left(L_{i}^{M}-L_{i}^{T}\right)}^{2},$$ (13)

where $G_{i}^{M,d{}^{\circ}}$ is the Gabor profiles with *d* degrees computed from the texture model and $G_{i}^{T,d{}^{\circ}}$ is computed from the deformed contour. The variables $L_{i}^{M}$ and $L_{i}^{T}$ are the Laws’ texture profiles computed from the texture model and the deformed contour, respectively. For these energy terms, 5x20 weights of energy terms for all the control points are computed using Eq (7).

### 2.10. ATASM for synovial sheath segmentation

Although the initial tendon localization is complicated, the localization of the synovial sheath is simple and can be defined based on the detected tendon position. In the synovial sheath segmentation, because the synovial sheath surrounds the tendon, the center position of the tendon is close to the center of the synovial sheath and can be used as the initial guess.

In synovial sheath segmentation, the definitions of the terms for curvature, Gabor, and Laws’ textures are the same as those in tendon segmentation. Because the synovial sheath is darker inside than outside, the gradient orientation on the synovial sheath boundary is defined from dark to bright, the opposite of the gradient orientation for the tendon. The area gradient and line gradient are thus modified as

$$F_{i}^{Area\_grad}=-{\bar{I}}_{i}^{inner\_area}+{\bar{I}}_{i}^{outer\_area};$$ (14)

$$F_{i}^{Line\_grad}=-{\bar{I}}_{i}^{inner\_line}+{\bar{I}}_{i}^{outer\_line}.$$ (15)

Maximizing these energy terms help with the detection of the synovial sheath boundary.

### 2.11. Post-processing of synovial sheath segmentation

**Fig 7:**
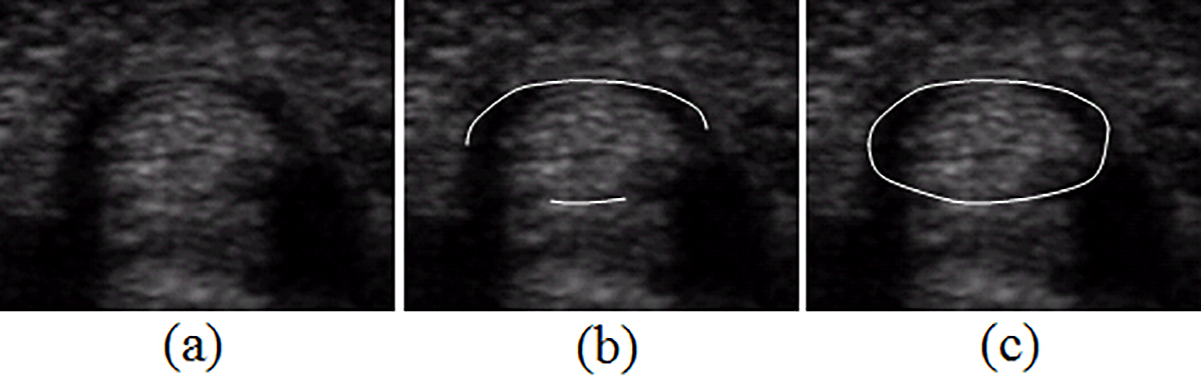
Boundary of the synovial sheath. (a) original image; (b) the lower part and upper parts of the synovial sheath boundary; (c) complete boundary after interpolation.

Because the lower portion of the synovial sheath connects to the volar plate and the tendon is tightly placed on the volar plate, the lower border of the synovial sheath is defined on the boundary between the tendon and the volar plate, as shown in Fig 7(B). The lower part of the tendon boundary within thirty degrees with respect to the y-axis is adopted as the lower synovial sheath boundary. Although the synovial sheath does have a closed boundary, the boundaries between the upper and lower endpoints of the synovial sheath are usually invisible in an ultrasound image. Therefore, we connect these endpoints using a second-order interpolation to approximate the synovial sheath boundaries on both the right and left sides as two parabolas. A segmentation example is shown in Fig 7.

## 3. Results and discussion

### 3.1. Experimental design

Due to the characteristics of the bottom side of the tendon boundary, all images are split into clear and fuzzy boundary groups by computing the average intensity difference between the two 15x15 windows above and below the bottom tendon boundary. If the average intensity of the window above is higher than the one below by 30, the image is placed in the clear boundary group. Otherwise, the image placed in the fuzzy boundary group. In each group, twenty images are used in the training phase. A total of thirty-eight images with clear boundaries and thirty-six images with fuzzy boundaries clustered from seventy-four images (S1 Dataset) are used in the experiments to evaluate the accuracy of the segmentation results. In the first experiment, the accuracy of proposed ATASM is evaluated. The ground truth is generated by a medical expert, Dr. T. H. Yang, who is asked to draw the boundaries of the tendon from seventy-four images separately three times in three consecutive weeks (once each week). The ground truth boundaries are then acquired by averaging the three boundaries (S2 Dataset). Three different segmentation methods, ACM, traditional ASM, and texture-based ASM which uses the same energy function as ATASM but with equivalent weights, are applied for comparison. Furthermore, two users who are trained to find the tendon in ultrasound are asked to outline the tendon boundaries from sixteen images. Each trained user is blinded to the ground truths and the results of the proposed method. These outlined results are used to evaluate the difference between the proposed ATASM method and human operation. In the second experiment, we evaluate the segmented synovial sheath using the proposed ATASM compared to the ground truth outlined by Dr. T. H. Yang. Thirty images with clear boundaries and twenty-seven images with fuzzy boundaries clustered from fifty-seven images are used in this experiment. In the last experiment, the segmentation results of the proposed system are used for the symptomatic vs. asymptomatic classification.

### 3.2. Evaluation metrics

To evaluate the segmentation results of the proposed method, two quantitative indexes, the mean of absolute distance (MAD) and the dice similarity coefficient (DSC), are used:

$$MAD=\frac{1}{2}\left[\frac{1}{\left|A\right|}\sum_{a\in A}\underset{b\in B}{\inf}d\left(a,b\right)+\frac{1}{\left|B\right|}\sum_{b\in B}\underset{a\in A}{\inf}d\left(a,b\right)\right];$$ (16)

$$DSC=\frac{2\left|A\cap B\right|}{\left|A\right|+\left|B\right|},$$ (17)

where *a* and *b* are the control points of contour *A* and *B* and *d*(*a*, *b*) is the distance between *a* and *b*. MAD calculates the average distance between the ground truth and the segmented contour. DSC evaluates the ratio of the overlapped area to the total area of the two contours. Conventionally, the two contours, *A* and *B*, are deemed similar if the DSC is higher than 0.75.

In the symptomatic vs. asymptomatic classification experiment, the confusion matrix is constructed using the classification results. Accuracy and precision are computed from the confusion matrix with

$$\mathrm{Accuracy}=\frac{TP+TN}{TP+TN+FP+FN};$$ (18)

$$\mathrm{Precision}=\frac{TP}{TP+FP},$$ (19)

where TP, TN, FP, and FN denote true positive, true negative, false positive, and false negative, respectively.

### 3.3. Accuracy of tendon segmentation

**Fig 8:**
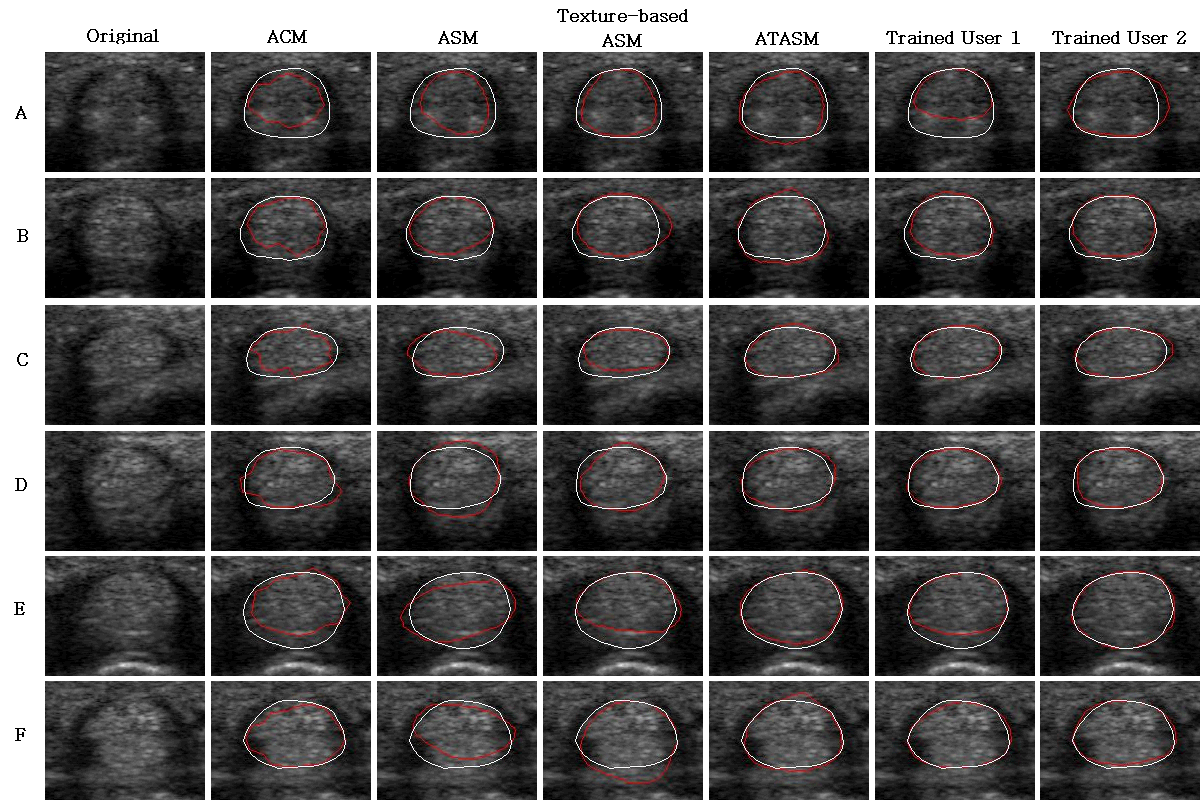
Resulting tendon segmentation images with different methods. The ground truths are indicated in white and the segmentation results are indicated in red. The first three rows are the tendons with clear boundaries and the remaining rows are with fuzzy boundaries.

The segmentation accuracies of tendons with clear and fuzzy boundary groups are shown in Tables 1 and 2, respectively. The average MAD values for tendon segmentation are 3.14 and 3.34 pixels, and the average DSC values are 0.91 and 0.90 for the two groups. Both average MAD and DSC values of the proposed ATASM are better than those of the other three segmentation methods for both groups. The first eight cases of the above two groups are also outlined by the two trained users. The results of proposed ATASM method are close to the ones of trained users. Fig 8 shows some tendon images (first column) and with the results of ACM (second column), traditional ASM (third column), texture-based ASM (fourth column), proposed ATASM (fifth column), trained user 1 (sixth column), and trained user 2 (seventh column). The first three rows are the images with clear boundaries and the remaining rows are the ones with fuzzy boundaries. Each resulting contour and ground truth are indicated as red and white line, respectively.

**Table 1:** Accuracy measure of tendon segmentation (clear boundary).

	ACM		ASM		texture-based ASM		ATASM		Trained User 1		Trained User 2	
Case	MAD	DSC	MAD	DSC	MAD	DSC	MAD	DSC	MAD	DSC	MAD	DSC
1	3.89	0.87	4.24	0.87	2.81	0.92	2.52	0.92	2.53	0.93	1.67	0.95
2	9.06	0.74	7.28	0.79	4.19	0.89	3.76	0.90	7.93	0.77	3.22	0.92
3	6.18	0.82	4.39	0.87	4.60	0.88	2.82	0.93	3.65	0.90	2.74	0.93
4	4.47	0.84	4.55	0.85	3.77	0.87	1.84	0.95	1.66	0.95	1.70	0.95
5	6.06	0.82	4.56	0.87	3.29	0.91	3.66	0.90	5.46	0.84	2.80	0.92
6	5.89	0.82	7.42	0.77	5.71	0.83	2.80	0.92	3.90	0.89	1.63	0.96
7	8.07	0.77	9.81	0.70	7.36	0.79	3.60	0.91	10.22	0.71	2.24	0.94
8	4.30	0.85	5.04	0.81	5.44	0.83	2.90	0.90	4.12	0.85	7.58	0.78
9	4.42	0.85	4.41	0.86	5.59	0.83	3.10	0.90	-	-	-	-
10	9.81	0.71	10.16	0.70	3.58	0.90	3.04	0.92	-	-	-	-
11	6.44	0.77	5.21	0.83	2.43	0.92	2.46	0.92	-	-	-	-
12	3.82	0.88	2.93	0.91	3.61	0.89	2.95	0.91	-	-	-	-
13	9.73	0.72	7.55	0.79	9.49	0.74	4.75	0.88	-	-	-	-
14	4.50	0.84	4.20	0.86	2.33	0.92	2.57	0.92	-	-	-	-
15	3.34	0.89	2.66	0.91	3.34	0.89	3.35	0.88	-	-	-	-
16	4.34	0.87	3.74	0.89	3.48	0.90	2.85	0.92	-	-	-	-
17	5.51	0.82	6.51	0.80	4.00	0.88	1.33	0.96	-	-	-	-
18	5.76	0.82	3.48	0.89	4.54	0.86	2.43	0.94	-	-	-	-
19	4.11	0.87	4.54	0.85	5.12	0.84	2.69	0.92	-	-	-	-
20	6.23	0.81	7.84	0.75	2.41	0.93	2.57	0.93	-	-	-	-
21	8.92	0.73	7.91	0.77	6.04	0.82	6.02	0.83	-	-	-	-
22	10.96	0.68	8.24	0.78	6.83	0.82	6.58	0.82	-	-	-	-
23	14.09	0.62	9.72	0.74	7.38	0.81	5.79	0.85	-	-	-	-
24	3.43	0.89	3.01	0.90	2.82	0.91	2.92	0.91	-	-	-	-
25	11.12	0.68	10.18	0.74	5.51	0.86	5.14	0.87	-	-	-	-
26	3.47	0.88	3.79	0.88	2.99	0.91	2.93	0.91	-	-	-	-
27	7.28	0.74	5.01	0.82	3.30	0.89	2.72	0.90	-	-	-	-
28	4.70	0.85	5.15	0.84	2.55	0.93	2.21	0.94	-	-	-	-
29	5.83	0.82	5.45	0.84	2.45	0.93	2.73	0.92	-	-	-	-
30	4.17	0.85	6.45	0.78	4.05	0.84	1.56	0.94	-	-	-	-
31	12.80	0.66	5.99	0.85	4.89	0.89	3.48	0.92	-	-	-	-
32	6.00	0.81	6.62	0.79	2.88	0.91	2.88	0.91	-	-	-	-
33	11.78	0.61	9.50	0.68	4.04	0.88	2.78	0.92	-	-	-	-
34	8.68	0.75	4.73	0.88	3.07	0.92	2.65	0.93	-	-	-	-
35	8.56	0.74	5.98	0.83	5.44	0.84	3.38	0.91	-	-	-	-
36	7.06	0.77	5.69	0.83	2.30	0.93	2.27	0.93	-	-	-	-
37	5.09	0.84	3.46	0.90	4.63	0.87	2.98	0.91	-	-	-	-
38	4.98	0.84	6.41	0.82	1.96	0.94	2.18	0.94	-	-	-	-
Average	6.71	0.79	5.89	0.82	4.22	0.88	3.14	0.91	4.93	0.85	2.95	0.92
									(Unit of MAD: pixel)			

**Table 2:** Accuracy measure of tendon segmentation (fuzzy boundary).

	ACM		ASM		texture-based ASM		ATASM		Trained User 1		Trained User 2	
Case	MAD	DSC	MAD	DSC	MAD	DSC	MAD	DSC	MAD	DSC	MAD	DSC
1	3.43	0.90	3.95	0.90	3.10	0.91	2.77	0.93	1.71	0.96	2.06	0.95
2	2.92	0.89	5.42	0.81	4.67	0.84	2.19	0.92	3.20	0.88	2.80	0.91
3	6.73	0.83	6.17	0.84	5.76	0.86	2.02	0.95	4.45	0.89	1.13	0.98
4	2.64	0.93	5.58	0.84	3.83	0.89	1.66	0.96	4.52	0.87	2.93	0.92
5	3.42	0.90	6.72	0.83	5.62	0.86	4.26	0.89	5.26	0.86	1.60	0.96
6	3.48	0.91	6.74	0.82	5.03	0.88	2.21	0.95	1.37	0.97	1.98	0.95
7	4.97	0.85	10.17	0.73	7.02	0.80	2.33	0.92	2.62	0.92	2.94	0.91
8	3.98	0.89	5.38	0.87	4.19	0.88	2.57	0.93	3.52	0.91	1.47	0.96
9	2.25	0.93	5.39	0.86	4.94	0.86	2.87	0.92	-	-	-	-
10	2.90	0.92	4.49	0.88	4.09	0.89	3.03	0.91	-	-	-	-
11	2.28	0.93	5.92	0.83	4.38	0.87	3.58	0.89	-	-	-	-
12	3.02	0.91	5.20	0.86	2.42	0.93	3.43	0.90	-	-	-	-
13	2.75	0.92	6.19	0.83	4.98	0.86	3.91	0.87	-	-	-	-
14	8.25	0.74	9.14	0.71	7.82	0.77	3.73	0.88	-	-	-	-
15	3.21	0.90	7.79	0.79	5.69	0.84	4.40	0.87	-	-	-	-
16	5.86	0.83	4.68	0.86	3.53	0.90	2.33	0.93	-	-	-	-
17	2.97	0.92	6.46	0.84	6.90	0.83	5.41	0.86	-	-	-	-
18	5.49	0.82	6.80	0.78	7.98	0.77	3.89	0.87	-	-	-	-
19	3.01	0.91	4.44	0.87	4.43	0.87	3.06	0.91	-	-	-	-
20	13.43	0.58	8.45	0.76	2.74	0.91	2.61	0.91	-	-	-	-
21	6.29	0.80	11.64	0.64	7.38	0.76	3.46	0.89	-	-	-	-
22	13.70	0.54	19.37	0.44	16.51	0.48	3.75	0.84	-	-	-	-
23	3.60	0.90	3.98	0.90	5.24	0.86	2.96	0.92	-	-	-	-
24	3.46	0.91	4.96	0.88	4.91	0.87	2.32	0.94	-	-	-	-
25	5.53	0.81	10.57	0.69	9.74	0.70	4.47	0.84	-	-	-	-
26	3.26	0.90	6.43	0.82	8.35	0.75	2.80	0.92	-	-	-	-
27	6.94	0.78	9.23	0.71	8.40	0.72	5.01	0.83	-	-	-	-
28	2.49	0.92	2.72	0.92	4.08	0.87	2.56	0.92	-	-	-	-
29	4.79	0.85	2.89	0.91	4.89	0.85	3.33	0.89	-	-	-	-
30	3.89	0.88	4.21	0.87	2.18	0.93	1.99	0.94	-	-	-	-
31	6.46	0.80	4.27	0.87	2.98	0.91	4.50	0.84	-	-	-	-
32	2.96	0.91	3.97	0.90	3.61	0.90	4.77	0.88	-	-	-	-
33	5.01	0.87	4.81	0.89	3.01	0.93	3.73	0.91	-	-	-	-
34	5.06	0.83	4.23	0.87	3.23	0.90	4.20	0.87	-	-	-	-
35	4.19	0.87	2.98	0.90	2.65	0.91	2.93	0.91	-	-	-	-
36	4.35	0.88	7.73	0.77	3.97	0.90	5.18	0.86	-	-	-	-
Average	4.69	0.86	6.36	0.82	5.28	0.85	3.34	0.90	3.33	0.91	2.11	0.94
									(Unit of MAD: pixel)			

### 3.4. Accuracy of synovial sheath segmentation

**Fig 9:**
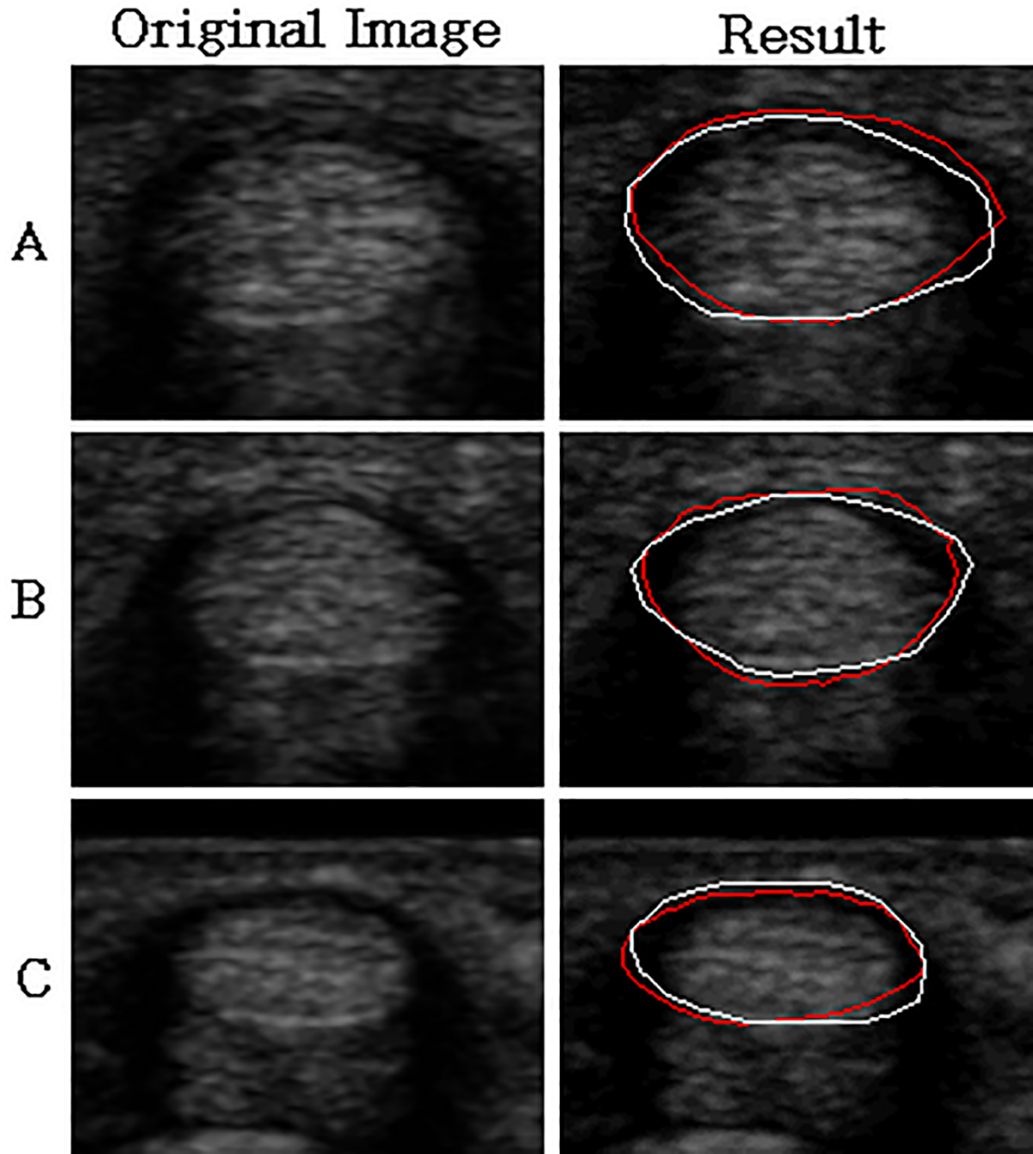
Image results for synovial sheath segmentation. The first column is the original images, and the second column is the segmented results (red line) and ground truth (white line).

As shown in Table 3, the average MAD values in images with clear and fuzzy boundaries are 5.12 and 4.54 pixels, respectively, and the average DSC values are 0.87 and 0.87, respectively. Compared with the ground truth, all the segmentation results exhibit DSC values higher than 0.75, which implies that the results are acceptable. Fig 9 shows the segmentation results of synovial sheath (red line) compared to the ground truth (white line).

**Table 3:** Accuracy measure of synovial sheath segmentation.

	Clear Boundary			Fuzzy Boundary	
Case	MAD	DSC	Case	MAD	DSC
1	4.43	0.88	1	4.68	0.89
2	6.43	0.86	2	3.01	0.93
3	2.93	0.92	3	4.33	0.87
4	7.98	0.80	4	3.99	0.89
5	4.80	0.89	5	4.57	0.88
6	5.10	0.86	6	2.28	0.93
7	7.11	0.76	7	5.35	0.85
8	5.21	0.86	8	6.33	0.86
9	2.65	0.93	9	2.83	0.94
10	3.12	0.92	10	4.62	0.87
11	5.58	0.85	11	5.85	0.81
12	5.17	0.87	12	4.00	0.87
13	4.32	0.87	13	4.63	0.84
14	7.38	0.85	14	5.56	0.86
15	3.98	0.92	15	3.62	0.91
16	4.48	0.89	16	3.37	0.88
17	4.80	0.90	17	6.22	0.84
18	3.13	0.91	18	6.49	0.79
19	2.78	0.91	19	1.53	0.96
20	7.08	0.81	20	5.41	0.84
21	5.30	0.85	21	3.69	0.90
22	6.54	0.77	22	3.80	0.90
23	9.61	0.84	23	6.16	0.86
24	4.86	0.87	24	4.56	0.91
25	5.27	0.85	25	3.91	0.90
26	4.76	0.91	26	4.79	0.87
27	4.20	0.89	27	7.06	0.84
28	3.60	0.92		-	-
29	3.86	0.92		-	-
30	7.05	0.85		-	-
Average	5.12	0.87		4.54	0.88
		(Unit of MAD: pixel)			

### 3.5. Symptomatic vs. asymptomatic classification

In this experiment, we compare the texture in the segmented tendon area to distinguish diseased tendons from normal. Two areas, the tendon area and the area inside the synovial sheath, and two types of texture features, a wavelet transform [29] and a gray level co-occurrence matrix [30], are used for symptomatic and asymptomatic finger classifications.

Wavelet transform is a commonly used transformation that decomposes data into high- and low-frequency parts in rows and columns by basis function. In this experiment, we use a stationary wavelet transform (SWT) and a wavelet packet (WP) to extract the features. The mean and standard deviation of each transformation image are computed to acquire features.

The gray level co-occurrence in four directions, 0°, 45°, 90°, and 135°, is used for feature extraction. A total of 12 features are described by Haralick et al. [30], and these features can be computed according to 1) Angular second moment, 2) Contrast, 3) Correlation, 4) Sum of squares variance, 5) Inverse difference moment, 6) Sum average, 7) Sum variance, 8) Sum entropy, 9) Entropy, 10) Difference variance, 11) Difference entropy, and 12) Information measures of correlation.

Due to the large number of features, we use a feature selection method to select the best feature set. An index called divergence value and described by Tsiaparas et al. in [31] is adopted:

$$DivergenceValue\left(f_{i}\right)=\frac{{\left(\sigma_{normal,f_{i}}-\sigma_{abnormal,f_{i}}\right)}^{2}\left(1+\sigma_{normal,f_{i}}+\sigma_{abnormal,f_{i}}\right)}{2\sigma_{normal,f_{i}}\sigma_{abnormal,f_{i}}},$$ (20)

where $\sigma_{normal,f_{i}}$ and $\sigma_{abnormal,f_{i}}$ are the standard deviations of the feature vector *f*_*i*_ for the normal and abnormal data sets. The more important feature shows the smaller variance within classes and the larger variance between classes and results in the larger divergence value.

A total of 42 pairs of finger images (S3 Dataset) are acquired in this experiment. Each image pair contains the left and right hand images from a subject. In all these image pairs, there are twenty-one symptomatic pairs and twenty-one asymptomatic pairs. In the symptomatic pairs, one of the finger images has trigger finger disease, and the contralateral one is normal. In the asymptomatic category, both finger images are normal. For each pair of finger images, the tendon and synovial sheath are first segmented. A 41x21 pixels area at the center of the segmented tendon is used to extract the above-mentioned texture features. For each pair of images, the features are extracted, and the differences in the features of both hands are computed. These feature differences are then selected using the above-mentioned feature selection process. The image pairs are then classified as symptomatic or asymptomatic using these selected feature differences. In recent years, the deep learning methods have been widely used for classification [32–34]. However, the amount of training data used to train the classification model is large. As the number of training data is not enough, deep learning method will be left for future investigation. Here, we used the support vector machine (SVM) for classification. In this study, a SVM library called LIBSVM [35], which uses second order information in the objective function for approximation [36] to attain a faster convergence than traditional SVM, is applied. The.

By computing the divergence value (Eq (20)) of each feature, we choose the most important 20 features from all the texture features for classification. The chosen features are given in Table 4, where *haar*, *db4*, *db6*, and *coif* are the different basis functions [31]. The confusion matrix is constructed and shown in Table 5. The system accuracy is 87.14%, and the precision is 82.38%. Based on the classification results, the selected geometric and texture features can be properly used for trigger finger identification.

**Table 4:** Selected features.

#	Feature	#	Feature
1	Area inside Sheath	11	SWT A1 (coif) mean
2	Area of tendon	12	SWT A1 (db4) mean
3	0° difference variance	13	WP A1 (haar) std.
4	WP A2A1 (haar) mean	14	SWT A1 (haar) std.
5	SWT A2A1 (db6) mean	15	45° Contrast
6	SWT A2A1 (coif) mean	16	WP Dv2Dv1 (haar) std.
7	SWT A2A1 (db4) mean	17	90° Contrast
8	SWT A2A1 (haar) mean	18	SWT A2A1 (db6) std.
9	WP A1 (haar) mean	19	0° sum variance
10	SWT A1 (db6) mean	20	90° sum average

**Table 5:** Confusion matrix of classification results.

		Predicted	
		Trigger Finger	Normal
Actual	Trigger Finger	173 (TP)	37 (FN)
	Normal	17 (FP)	193 (TN)

## 4. Discussion

For tendon segmentation, the proposed ATASM achieves the lowest MAD values (3.14 and 3.34 pixels for clear and fuzzy boundary groups) and the highest DSC value (0.91 and 0.90 for clear and fuzzy boundary groups) among the adopted automatic methods as shown in Tables 1 and 2. The results of proposed ATASM are better than ACM (MAD = 6.71 and 4.69 pixels, DSC = 0.79 and 0.86), traditional ASM (MAD = 5.89 and 6.36 pixels, DSC = 0.82 and 0.82), and texture-based ASM (MAD = 4.22 and 5.27 pixels, DSC = 0.88 and 0.85). The segmentation results of ACM are rugged in many cases as shown in the second column of Fig 8 due to the lack of shape information. The traditional ASM contains the information of tendon shape from training images and obtains the more applicable tendon boundary than ACM. However, the energy term which only considers the gradient information is easily affected by the speckle noise. By adding the texture information in energy terms as in the texture-based ASM, the segmentation results are improved and less affected by the noise. Since the top boundary of the tendon surrounded by a narrow band of synovial fluid, and the boundaries on both sides of the tendon are usually blurry, the texture-based ASM using equivalent weights of energy terms is less effective in segmentation as shown in the fourth column of Fig 8. By adopting different weights of energy terms at different positions, the proposed ATASM can segment the upper and lower boundaries of tendon more precisely as shown in the fifth column of Fig 8. Compared to the two trained users, the proposed ATASM achieves the better results than user 1 (MAD = 4.93 and 3.33 pixels, DSC = 0.85 and 0.91), and close to the ones of user 2 (MAD = 2.95 and 2.11 pixels, DSC = 0.92 and 0.94). The user 1 obtains the worse MAD and DSC values because the bottom areas of tendon are misjudged as volar plate in two cases, as shown in the cases A and E of Fig 8. It means that the proposed method can obtain accurate results similar to the human operation.

In the synovial sheath segmentation, all the segmentation results for clear and fuzzy boundary cases are higher than 0.85 in averaged DSC values which imply good correspondence with the expert outlines. As shown in Fig 9, the top side of the synovial sheath can be correctly segmented by using the proposed ATASM. The boundary on the bottom side of the synovial sheath is also well segmented by using the good tendon segmentation results obtained in the previous step.

**Fig 10:**
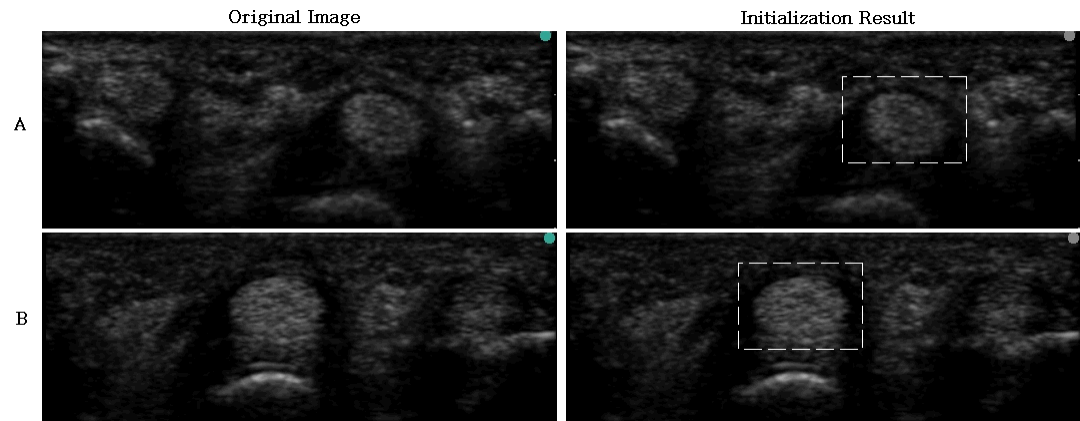
Model matching results. (a) image with clear boundary; (b) image with fuzzy boundary.

**Fig 11:**
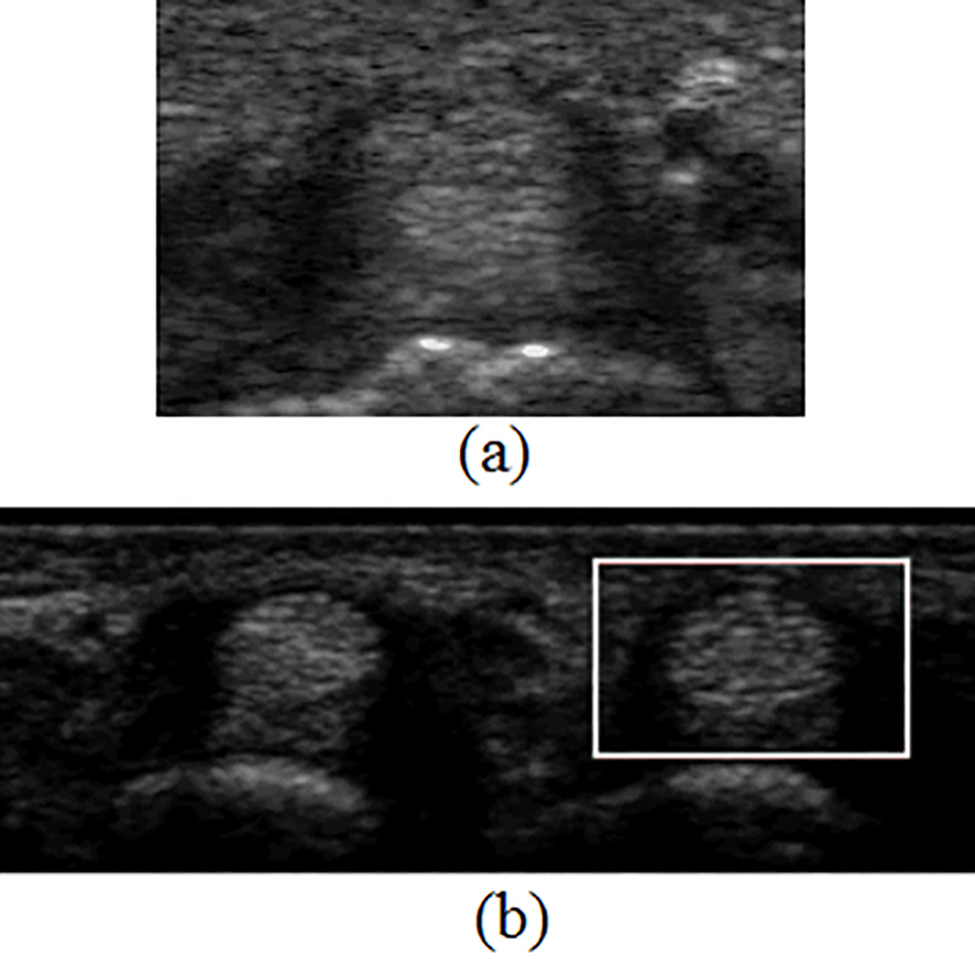
Special cases of finger image. (a) less synovial sheath area; (b) contains multiple tendons.

We also proposed the coarse-to-fine model matching for automatically locating the initial block of tendon as shown in Fig 10. Since the proposed intensity template containing both the tendon and surrounding synovial areas, which provide the necessary information for automatically locating the tendon block, the initial tendon block can be detected automatically. However, for those images with less synovial sheath areas or containing two tendons as in Fig 11(A) and 11(B), the tendon block is outlined manually.

As GA is adopted in ATASM for parameter selection, the segmentation process is repeated three times for different initials of GA and tested with five different images, and the resulting standard deviation of MAD is 0.52 pixels. This result shows that the proposed method can obtain the stable segmentation results with GA structure.

In order to test the stability of the proposed method with different training data sets, we had randomly selected ten training data from the tendon images with clear boundaries for model construction. Each set contains ten training images randomly selected from twenty training data. The constructed model was then applied to the thirty-eight images with clear boundaries to obtain the average MAD and DSC values of all segmentation results. The average MAD values of five trials were 4.07, 4.02, 4.04, 4.01 and 3.84 pixels. And the average DSC values were 0.89, 0.89, 0.88, 0.89, and 0.89. The standard deviation of MAD and DSC values of five trials were 0.08 pixels and 0.002, respectively. These results implied that the proposed ATASM structure with different training data set can achieve the good and similar segmentation results.

Our system also performs well with regard to classifying trigger finger and normal cases in the last experiment. The features selected by divergence value shows that the tissue areas and the low-frequency components of the wavelet transform, e.g., the A1 and A2A1 features, are the important features for classification, which satisfies the results of previous research [2–4] that the tendon with trigger finger may become thick and hypoechoic.

## 5. Conclusion

In this study, we develop a fully automatic segmentation system for the tendon and synovial sheath (S1 File). Because the characteristics of the tendon and synovial sheath boundaries exhibit large variations in ultrasound images, the conventional ASM cannot always achieve accurate segmentation results. The proposed ATASM can segment the tissue, which can be described using a statistical shape model with some variations in ultrasound images. The ATASM adopts the Gabor and Laws’ profiles as the texture features of tendon borders and uses adapted weighting to adjust the influence of energy terms at each control point in the energy function. Coarse-to-fine model matching is adopted for initializing the affine parameters of the ATASM model. GA-based optimization is used to maximize the energy function to determine the final organ shape. In the experiments, the MAD values are 3.14 and 3.34 pixels and the DSC values are 0.91 and 0.90 between our method and ground truth for the two image groups. These results are better than the ones by ACM, traditional ASM, and texture-based ASM, and similar to the human operations. In synovial sheath segmentations, the MAD values are 5.12 and 4.54 pixels and the DSC values are 0.87 and 0.88 that also implies good correspondence with the ground truth for synovial sheath. With the segmented area and the texture features, the proposed system performs well in classifying the normal and trigger fingers in the current clinical experiment.

The proposed study still has some limitations. Because there are acoustic shadows on the bottom two sides of synovial sheath in the axial ultrasound image, the parabolic interpolation is adopted in defining the sheath boundaries. For a better model of synovial sheath, we will acquire the contour shape either from other imaging modalities or with different ultrasound scanning structure. In addition, a larger number of subject images may also be acquired for further improving the tendon symptom classifications. Other finger symptoms, such as the de Quervain's syndrome, can also be included in the future clinical experiments.

## Supporting information

S1 Dataset Tendon images for segmentation. (ZIP)

S2 Dataset Ground truth of Testing images: Each text file contains the ground trurh contour of corresponding image with the same file name. Each row in the file is the point coordinate (X,Y) on the contour.
(ZIP)

S3 Dataset Tendon image pairs for classification. (ZIP)

S1 File Software of ATASM. (ZIP)
