# Supplementary figures and images for: A medical imaging analysis system for trigger finger using an adaptive texture-based active shape model (ATASM) in ultrasound images (part 1 of 2)
Source: PLoS One. 2017 Oct 27;12(10):e0187042. doi: 10.1371/journal.pone.0187042 (PMC5659776; doi:10.1371/journal.pone.0187042)

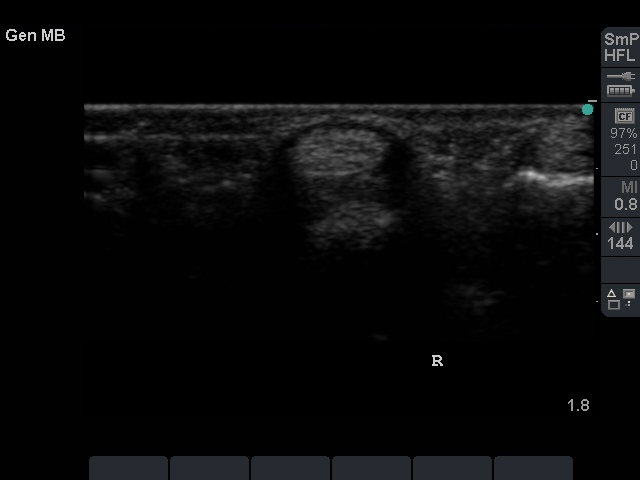

Supplement: S1 Dataset — (ZIP) [file pone.0187042.s001.zip › SegmentationData/253_L3T.bmp]

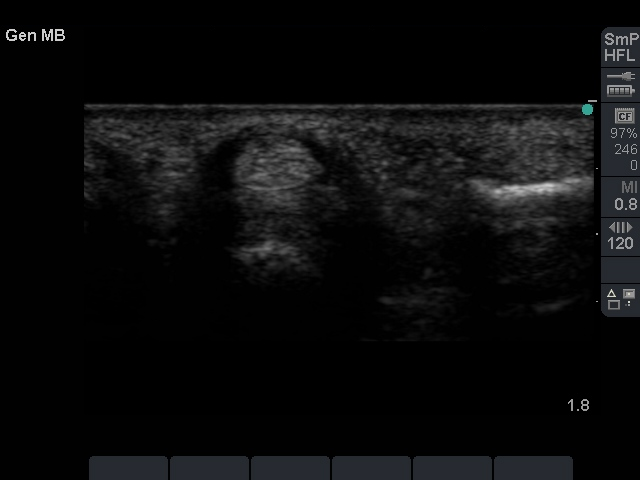

Supplement: S1 Dataset — (ZIP) [file pone.0187042.s001.zip › SegmentationData/253_R3T.bmp]

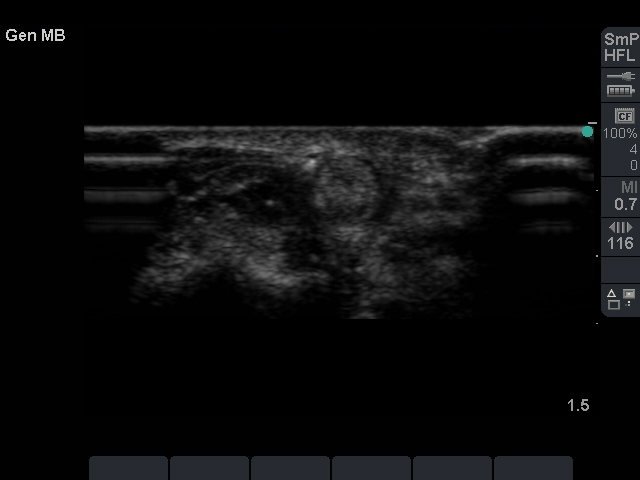

Supplement: S1 Dataset — (ZIP) [file pone.0187042.s001.zip › SegmentationData/254_L1T.bmp]

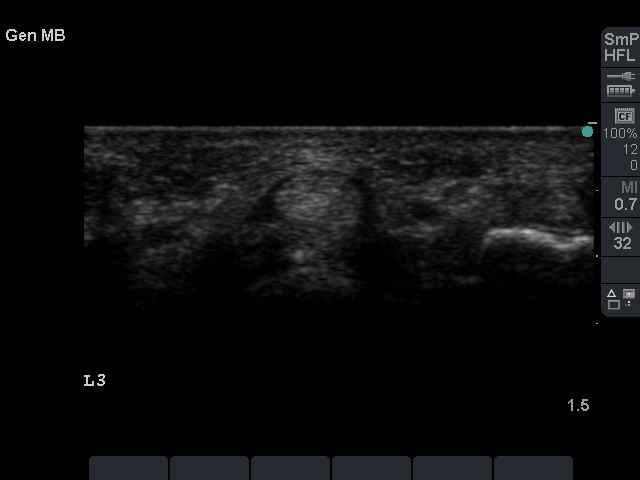

Supplement: S1 Dataset — (ZIP) [file pone.0187042.s001.zip › SegmentationData/254_L3T.bmp]

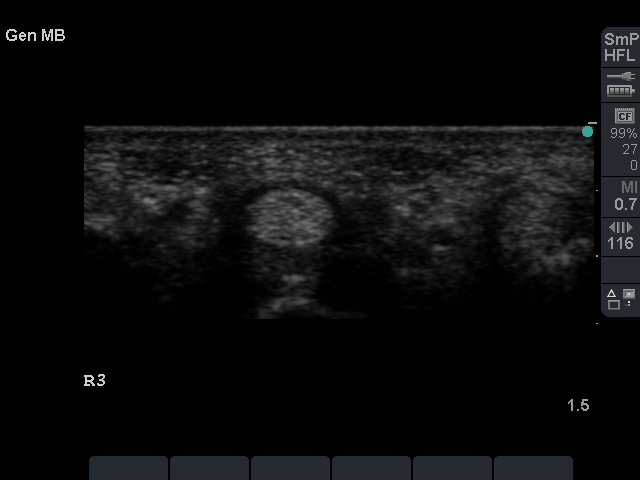

Supplement: S1 Dataset — (ZIP) [file pone.0187042.s001.zip › SegmentationData/254_R3T.bmp]

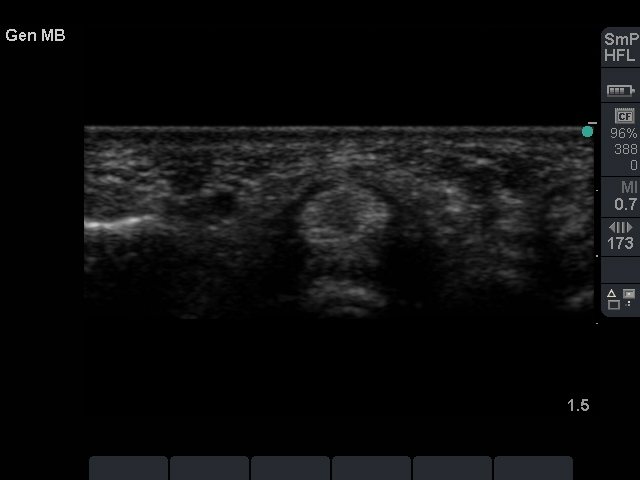

Supplement: S1 Dataset — (ZIP) [file pone.0187042.s001.zip › SegmentationData/256_R4T.bmp]

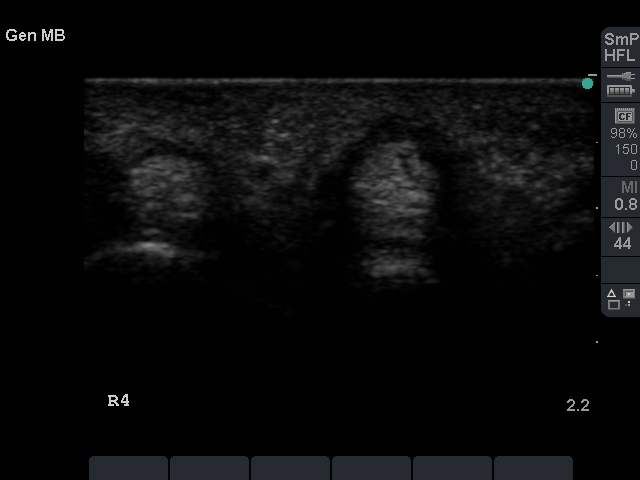

Supplement: S1 Dataset — (ZIP) [file pone.0187042.s001.zip › SegmentationData/257_R4T.bmp]

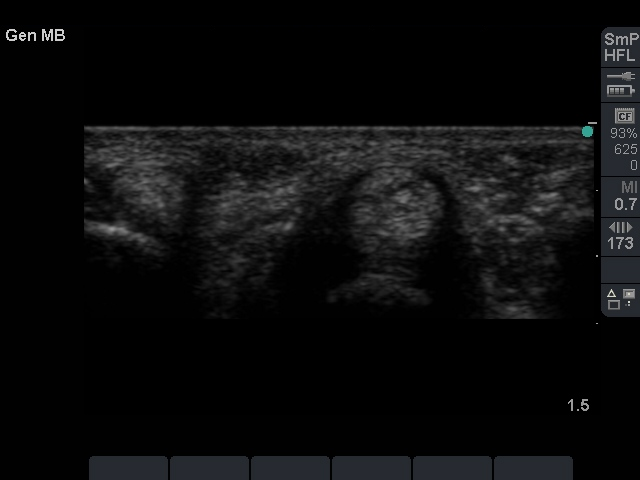

Supplement: S1 Dataset — (ZIP) [file pone.0187042.s001.zip › SegmentationData/258_R3T.bmp]

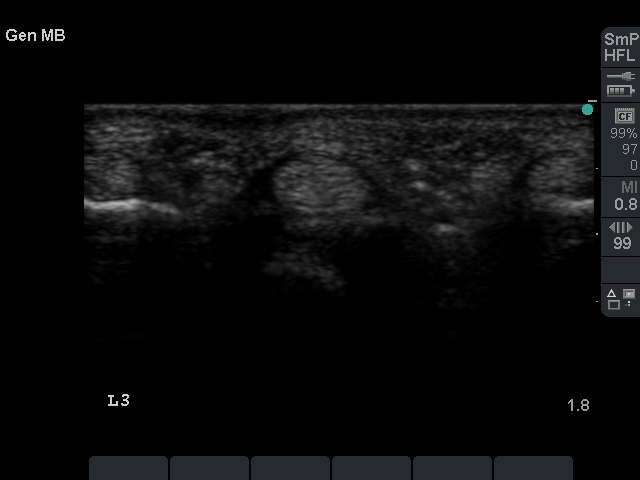

Supplement: S1 Dataset — (ZIP) [file pone.0187042.s001.zip › SegmentationData/263_L3T.bmp]

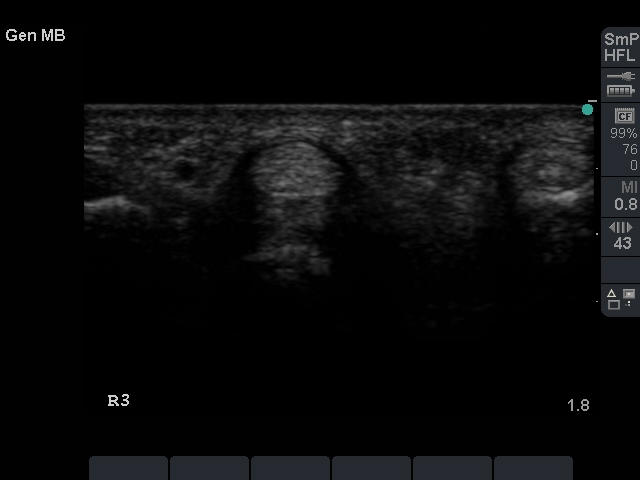

Supplement: S1 Dataset — (ZIP) [file pone.0187042.s001.zip › SegmentationData/263_R3T.bmp]

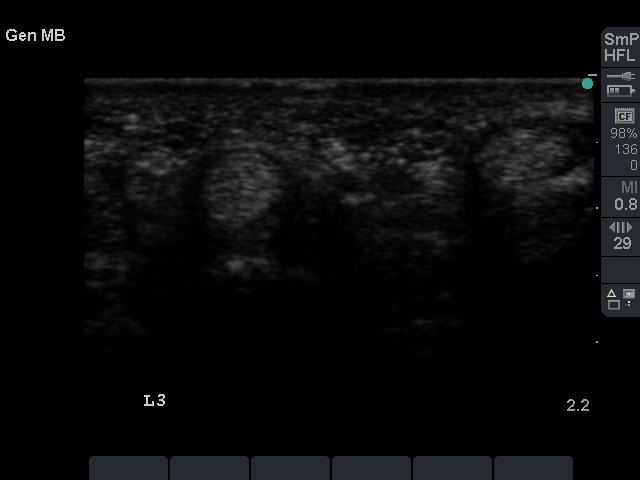

Supplement: S1 Dataset — (ZIP) [file pone.0187042.s001.zip › SegmentationData/265_L3T.bmp]

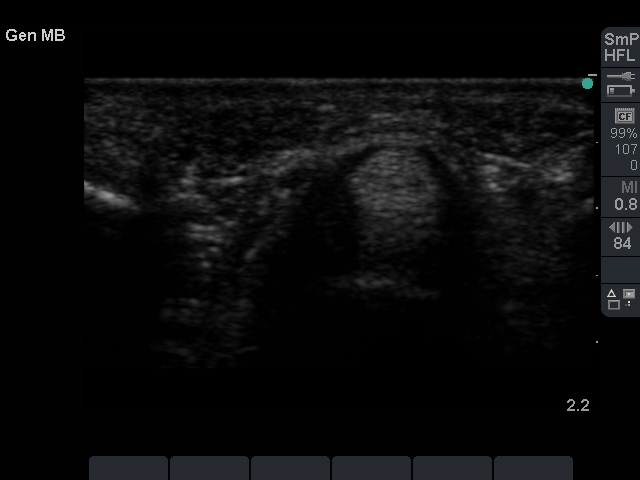

Supplement: S1 Dataset — (ZIP) [file pone.0187042.s001.zip › SegmentationData/265_R3T.bmp]

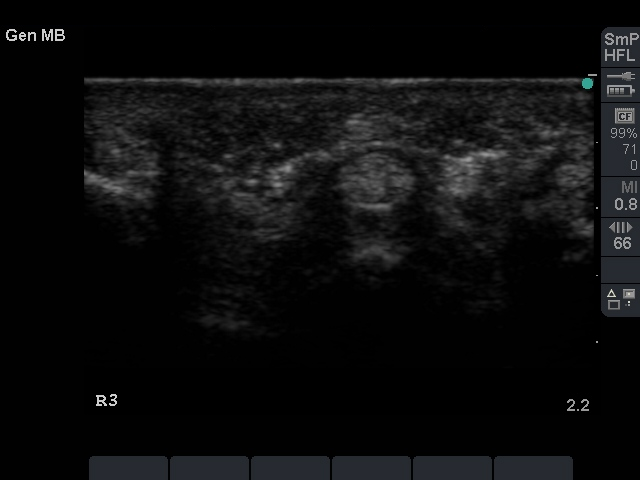

Supplement: S1 Dataset — (ZIP) [file pone.0187042.s001.zip › SegmentationData/272_R3T.bmp]

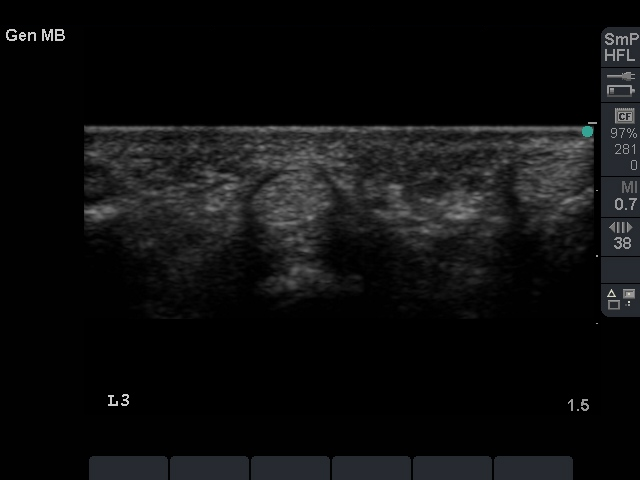

Supplement: S1 Dataset — (ZIP) [file pone.0187042.s001.zip › SegmentationData/277_L3T.bmp]

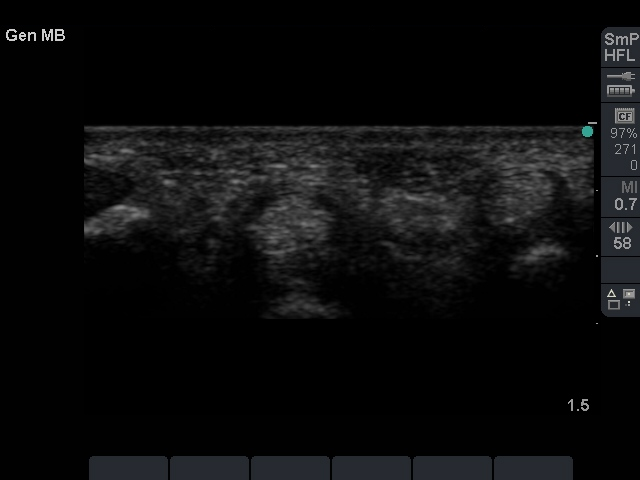

Supplement: S1 Dataset — (ZIP) [file pone.0187042.s001.zip › SegmentationData/277_R3T.bmp]

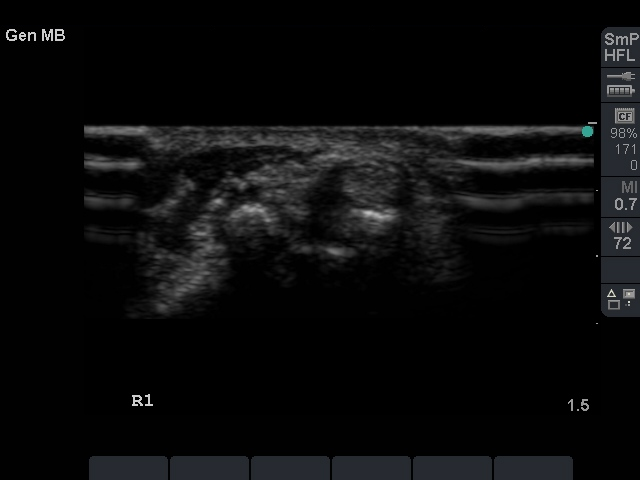

Supplement: S1 Dataset — (ZIP) [file pone.0187042.s001.zip › SegmentationData/280_R1T.bmp]

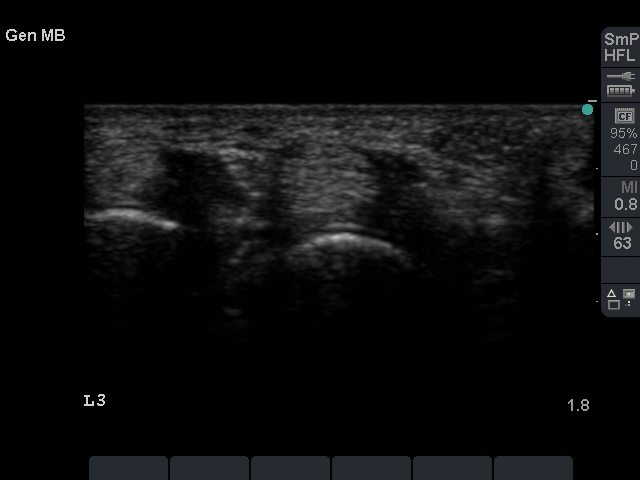

Supplement: S1 Dataset — (ZIP) [file pone.0187042.s001.zip › SegmentationData/284_L3T.bmp]

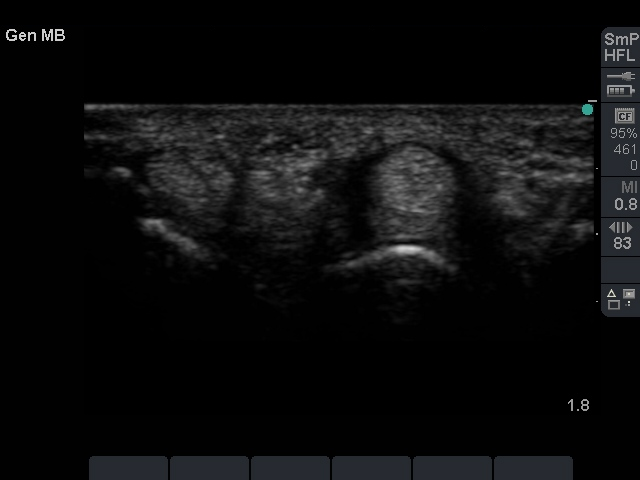

Supplement: S1 Dataset — (ZIP) [file pone.0187042.s001.zip › SegmentationData/284_R3T.bmp]

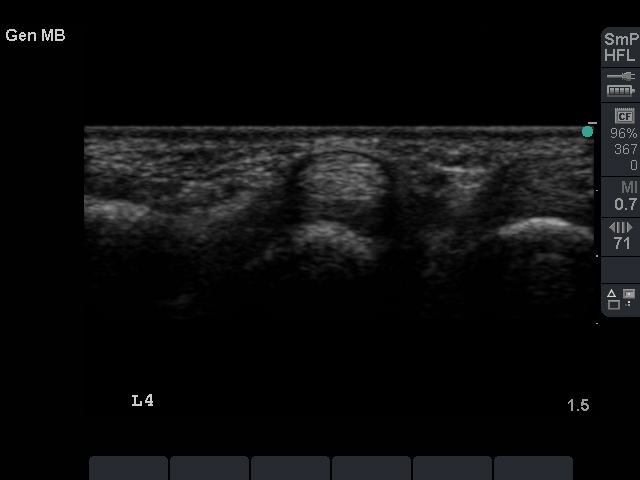

Supplement: S1 Dataset — (ZIP) [file pone.0187042.s001.zip › SegmentationData/290_L4T.bmp]

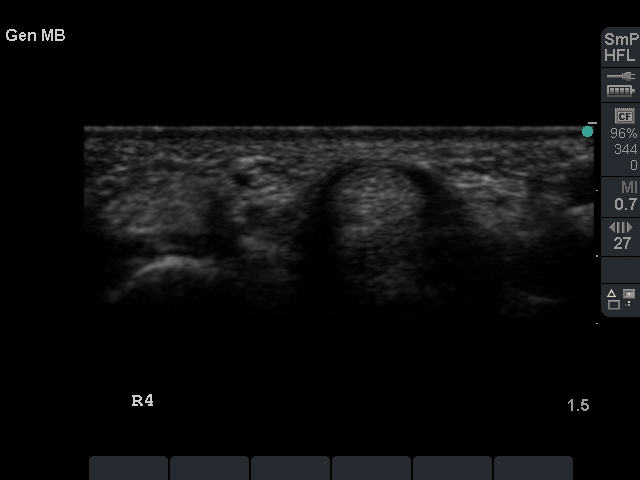

Supplement: S1 Dataset — (ZIP) [file pone.0187042.s001.zip › SegmentationData/290_R4T.bmp]

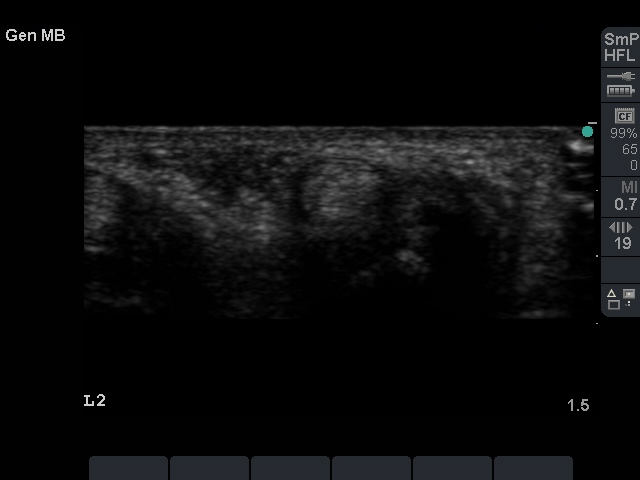

Supplement: S1 Dataset — (ZIP) [file pone.0187042.s001.zip › SegmentationData/293_L2T.bmp]

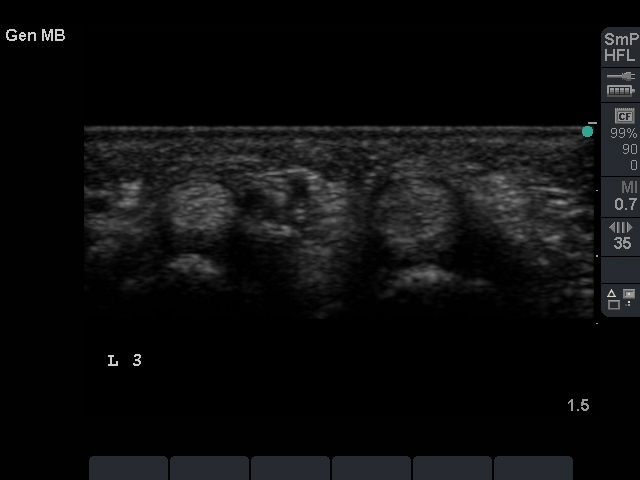

Supplement: S1 Dataset — (ZIP) [file pone.0187042.s001.zip › SegmentationData/299_L3T.bmp]

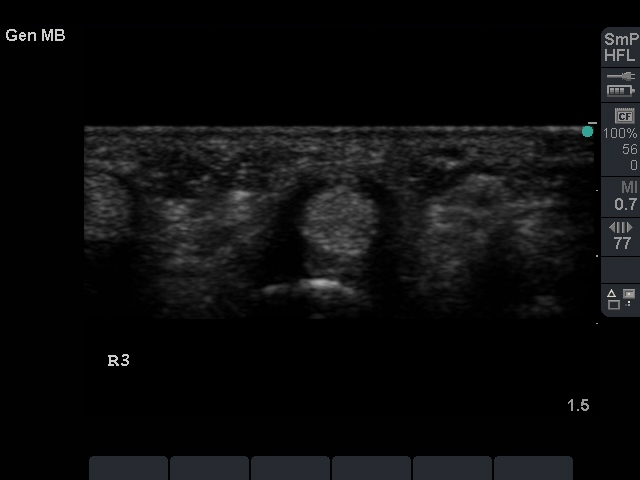

Supplement: S1 Dataset — (ZIP) [file pone.0187042.s001.zip › SegmentationData/299_R3T.bmp]

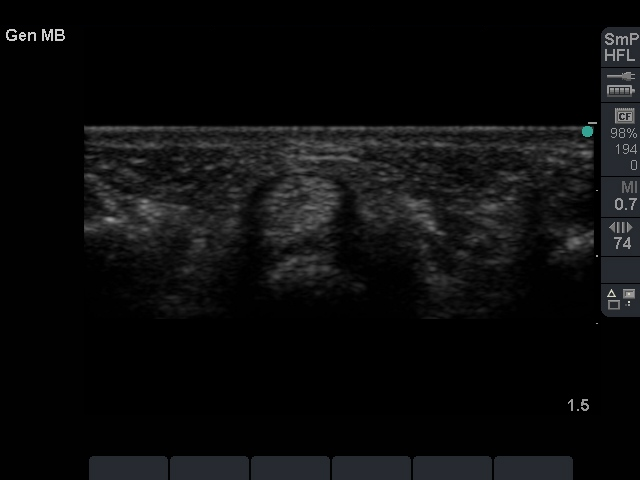

Supplement: S1 Dataset — (ZIP) [file pone.0187042.s001.zip › SegmentationData/36.bmp]

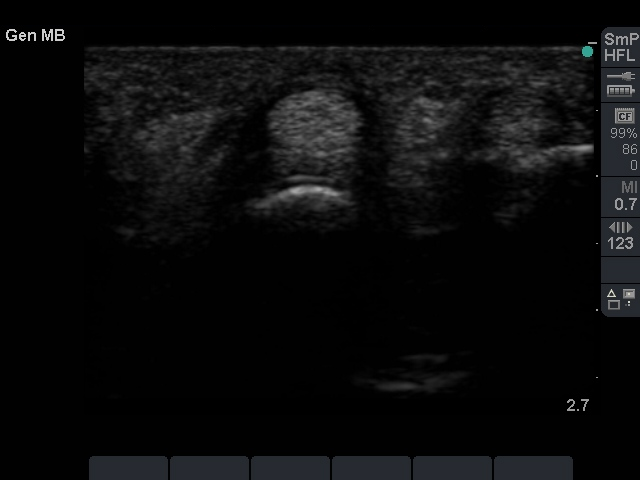

Supplement: S1 Dataset — (ZIP) [file pone.0187042.s001.zip › SegmentationData/39.bmp]

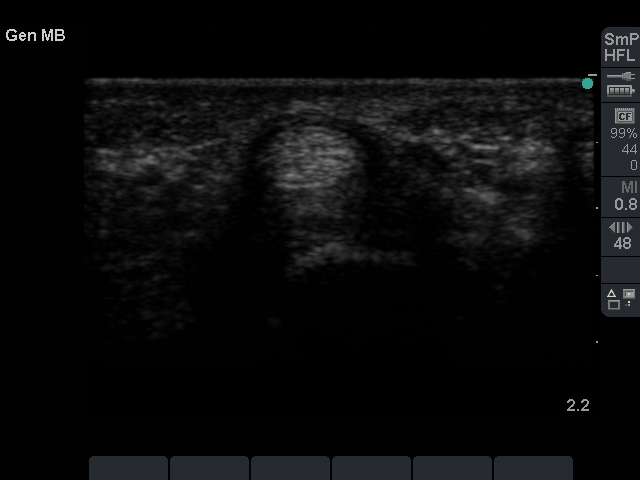

Supplement: S1 Dataset — (ZIP) [file pone.0187042.s001.zip › SegmentationData/74.bmp]

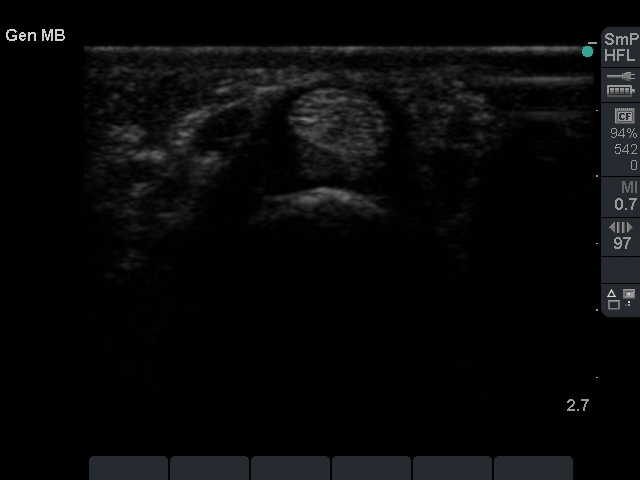

Supplement: S1 Dataset — (ZIP) [file pone.0187042.s001.zip › SegmentationData/88.bmp]

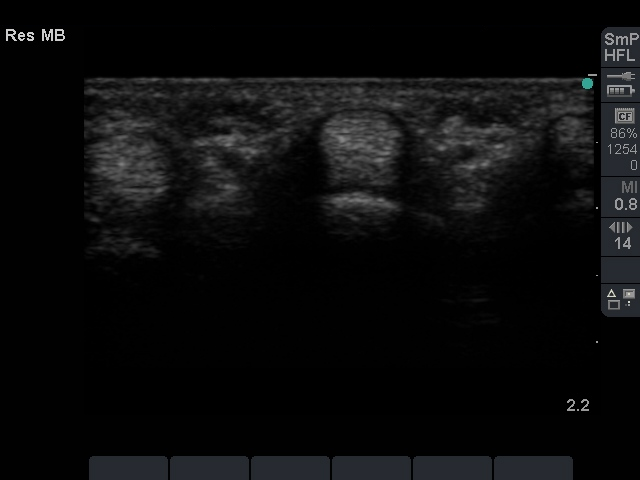

Supplement: S1 Dataset — (ZIP) [file pone.0187042.s001.zip › SegmentationData/92.bmp]

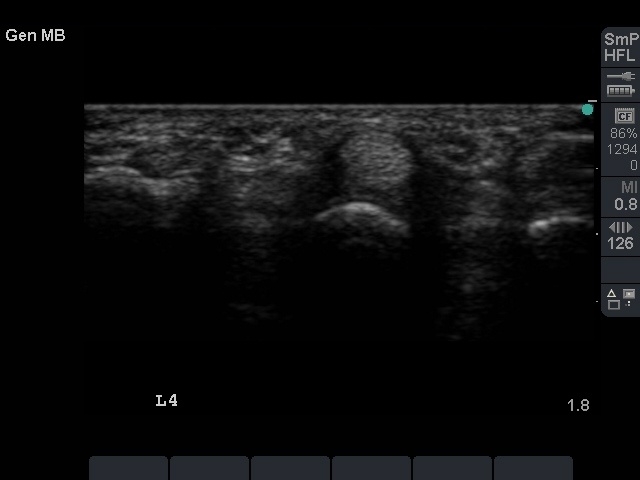

Supplement: S1 Dataset — (ZIP) [file pone.0187042.s001.zip › SegmentationData/93.bmp]

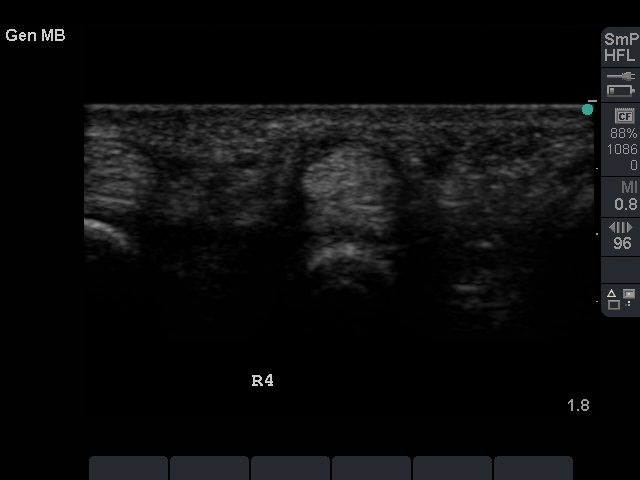

Supplement: S1 Dataset — (ZIP) [file pone.0187042.s001.zip › SegmentationData/95.bmp]

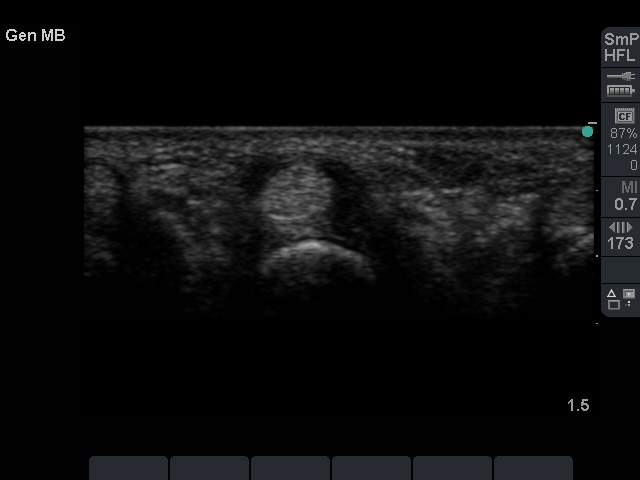

Supplement: S1 Dataset — (ZIP) [file pone.0187042.s001.zip › SegmentationData/96.bmp]

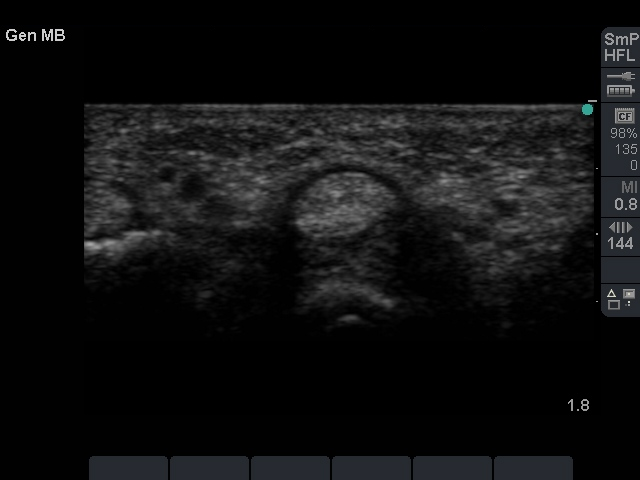

Supplement: S1 Dataset — (ZIP) [file pone.0187042.s001.zip › SegmentationData/99.bmp]

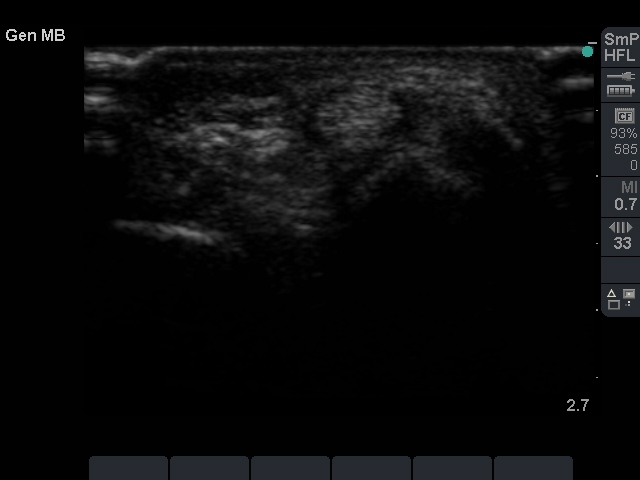

Supplement: S1 Dataset — (ZIP) [file pone.0187042.s001.zip › SegmentationData/A0.bmp]

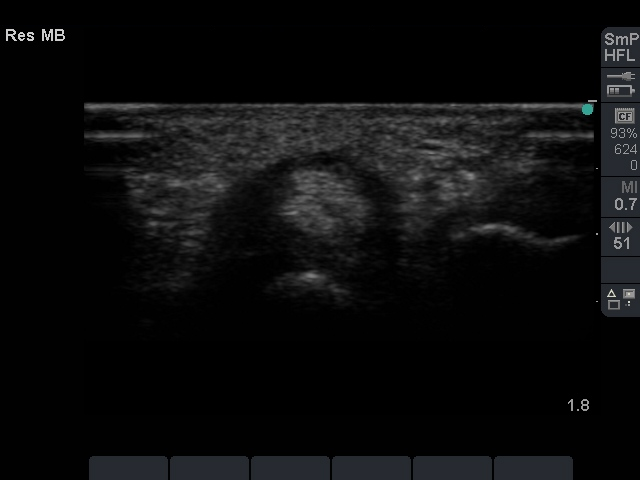

Supplement: S1 Dataset — (ZIP) [file pone.0187042.s001.zip › SegmentationData/A1.bmp]

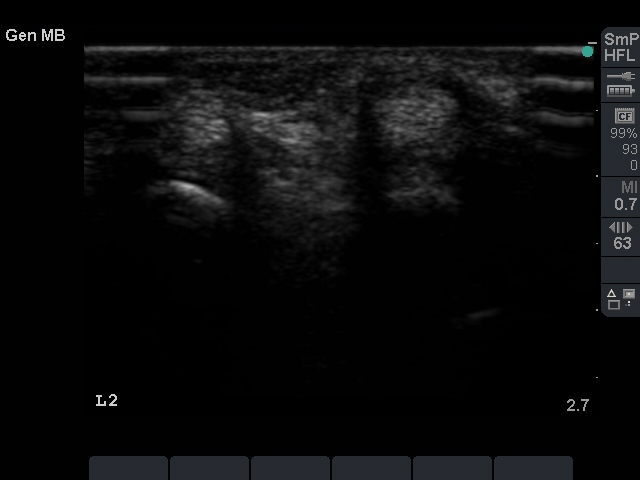

Supplement: S1 Dataset — (ZIP) [file pone.0187042.s001.zip › SegmentationData/A2.bmp]

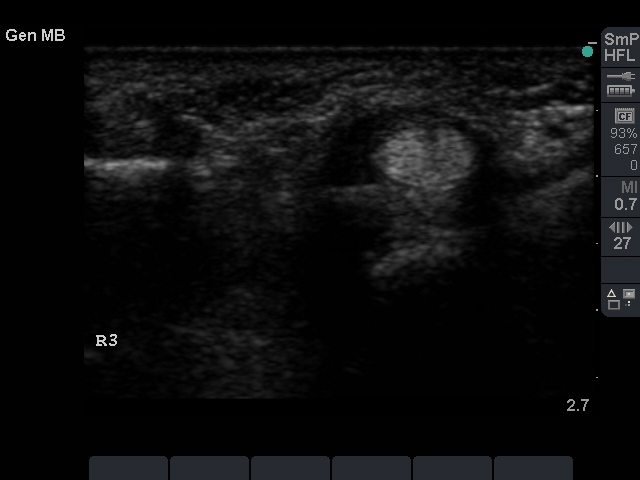

Supplement: S1 Dataset — (ZIP) [file pone.0187042.s001.zip › SegmentationData/A3.bmp]

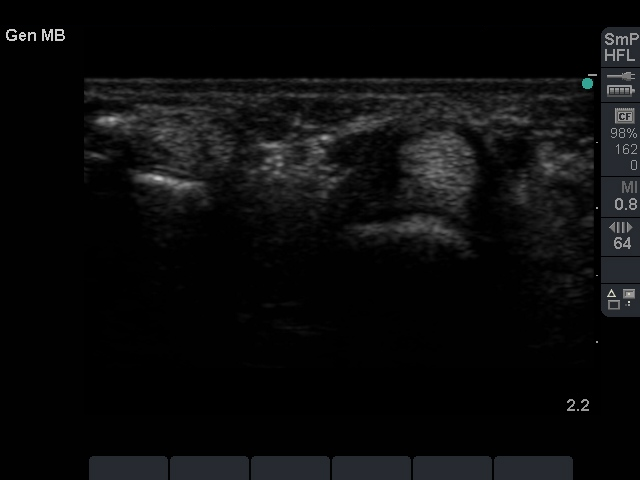

Supplement: S1 Dataset — (ZIP) [file pone.0187042.s001.zip › SegmentationData/A4.bmp]

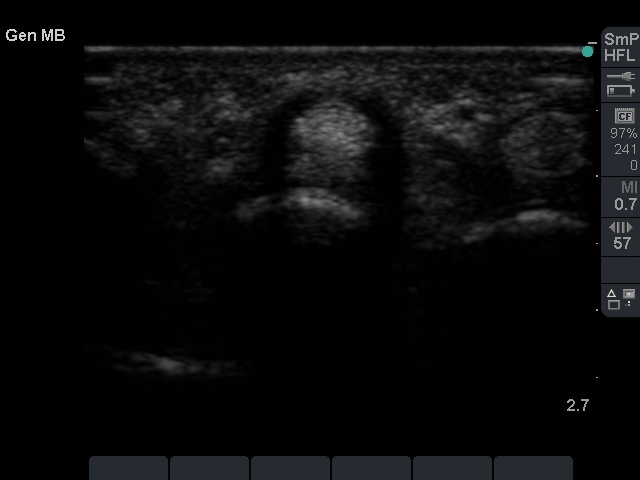

Supplement: S1 Dataset — (ZIP) [file pone.0187042.s001.zip › SegmentationData/A5.bmp]

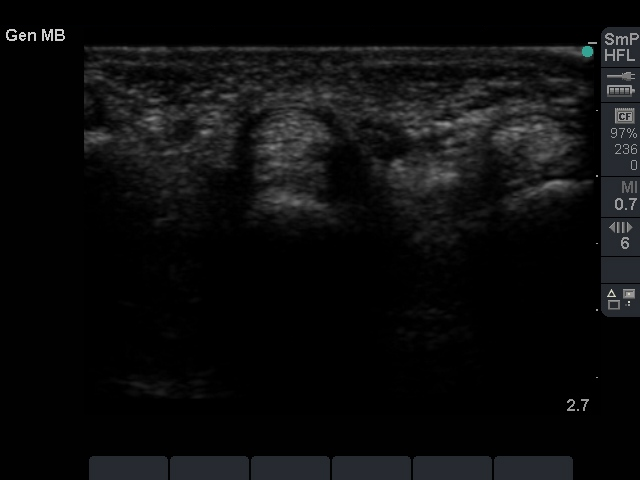

Supplement: S1 Dataset — (ZIP) [file pone.0187042.s001.zip › SegmentationData/A6.bmp]

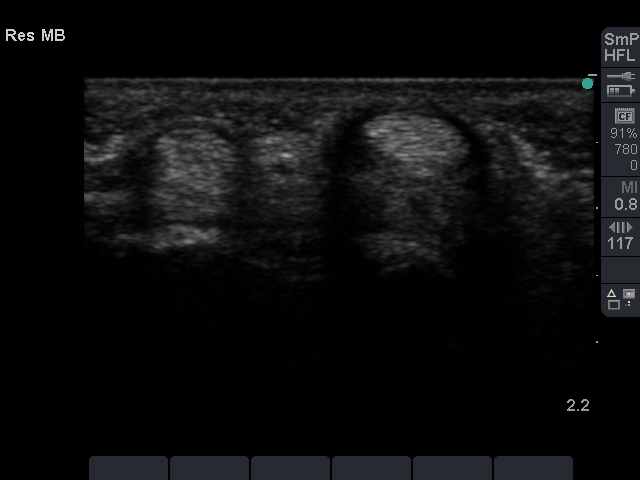

Supplement: S1 Dataset — (ZIP) [file pone.0187042.s001.zip › SegmentationData/A7.bmp]

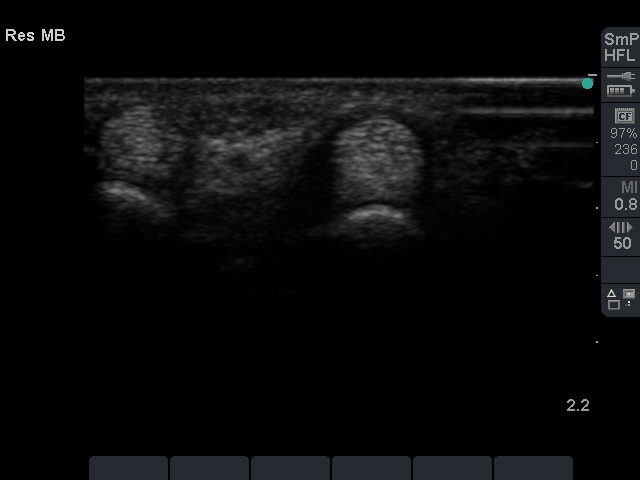

Supplement: S1 Dataset — (ZIP) [file pone.0187042.s001.zip › SegmentationData/A9.bmp]

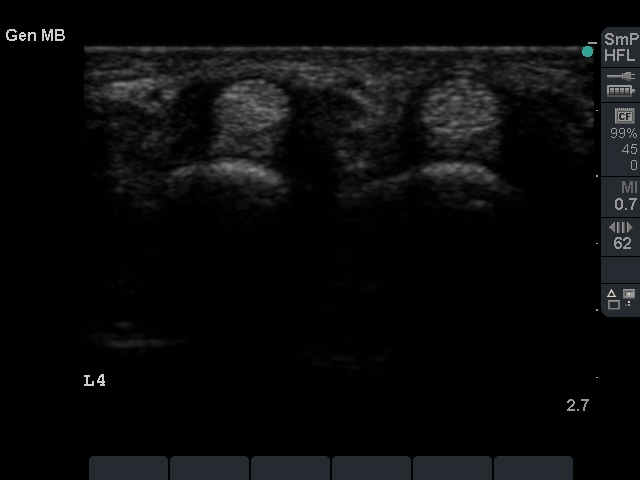

Supplement: S1 Dataset — (ZIP) [file pone.0187042.s001.zip › SegmentationData/B1.bmp]

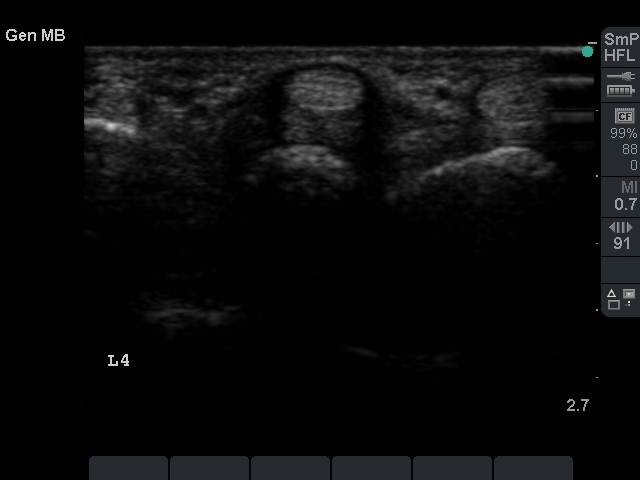

Supplement: S1 Dataset — (ZIP) [file pone.0187042.s001.zip › SegmentationData/B3.bmp]

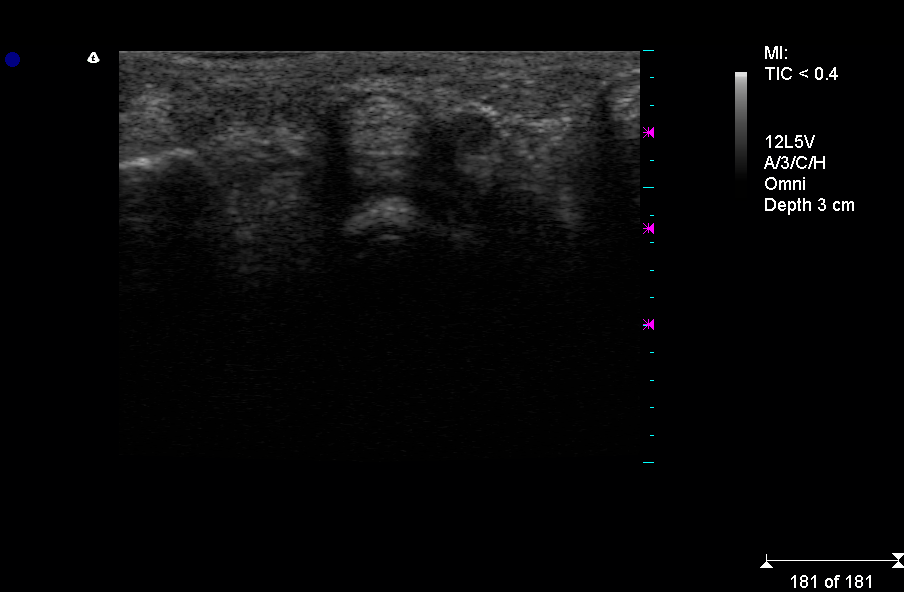

Supplement: S1 Dataset — (ZIP) [file pone.0187042.s001.zip › SegmentationData/CCK101124.bmp]

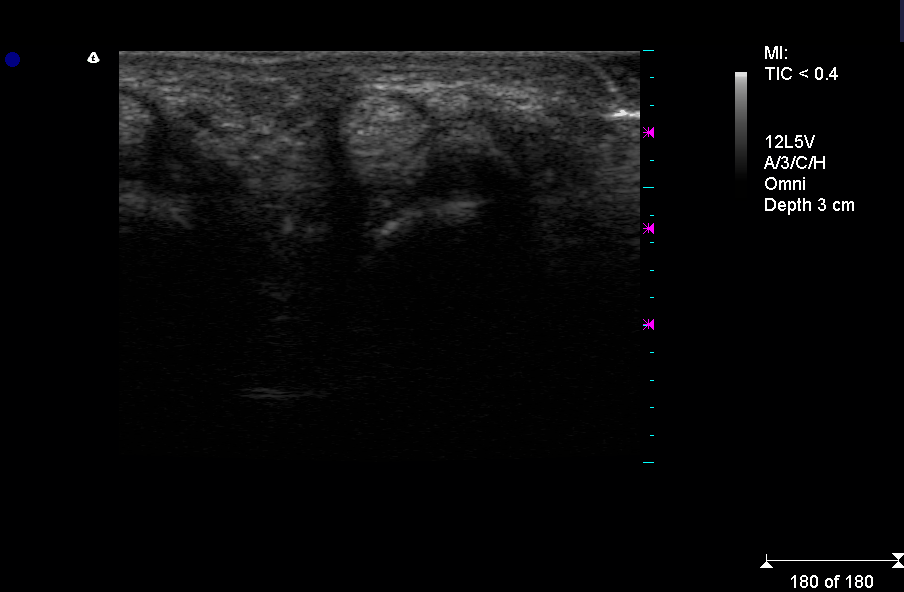

Supplement: S1 Dataset — (ZIP) [file pone.0187042.s001.zip › SegmentationData/CCS11142523_2.bmp]

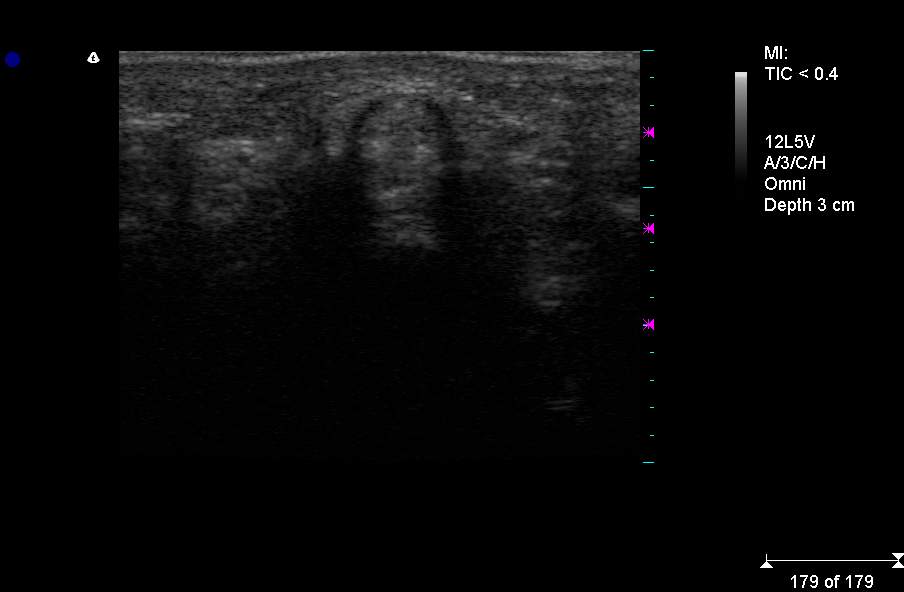

Supplement: S1 Dataset — (ZIP) [file pone.0187042.s001.zip › SegmentationData/CCS11142523_4.bmp]

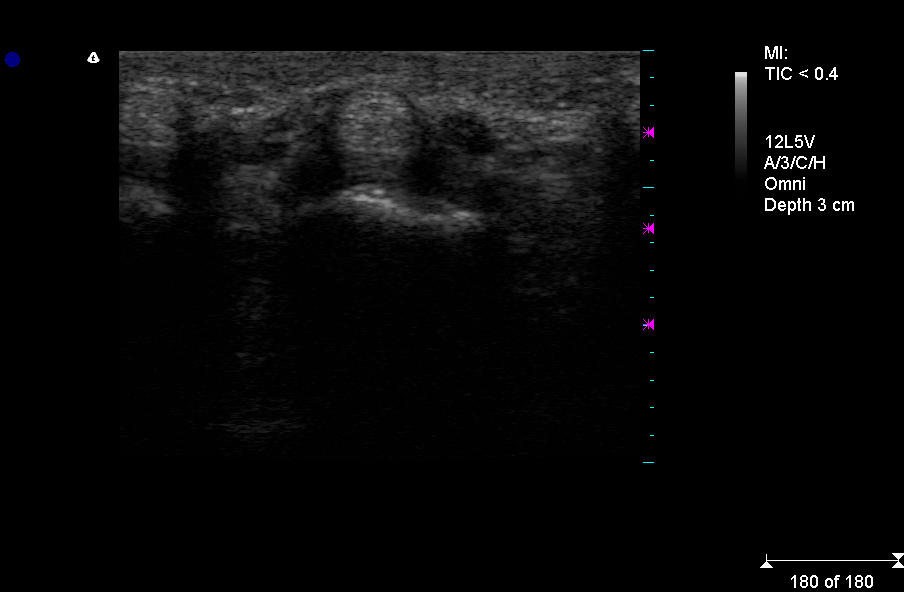

Supplement: S1 Dataset — (ZIP) [file pone.0187042.s001.zip › SegmentationData/CPY101123.bmp]

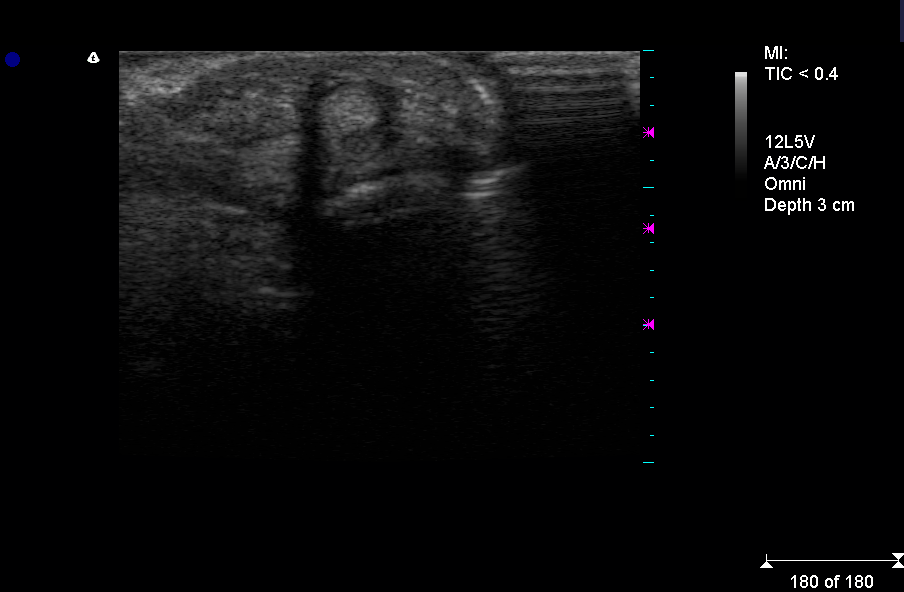

Supplement: S1 Dataset — (ZIP) [file pone.0187042.s001.zip › SegmentationData/CSZ6393945.bmp]

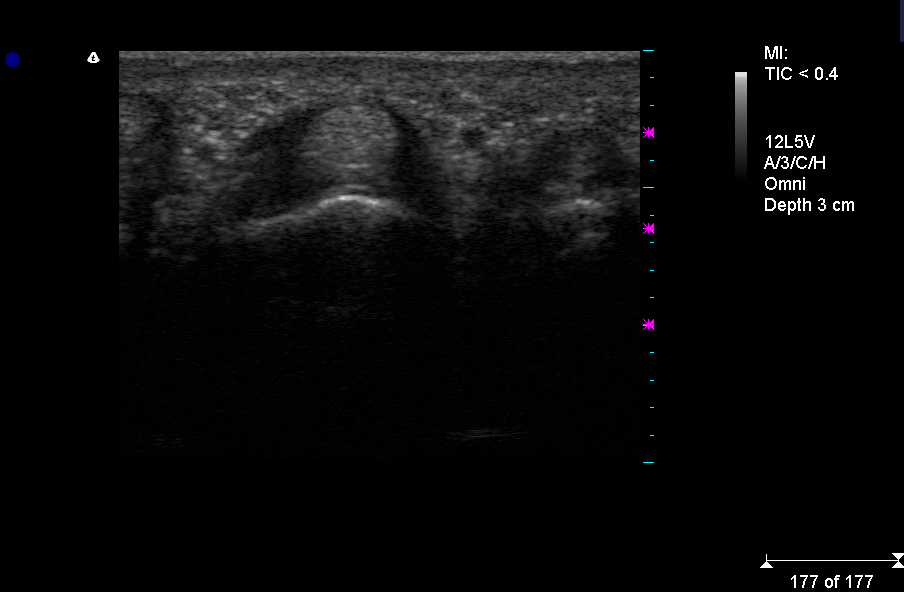

Supplement: S1 Dataset — (ZIP) [file pone.0187042.s001.zip › SegmentationData/CWC8488972.bmp]

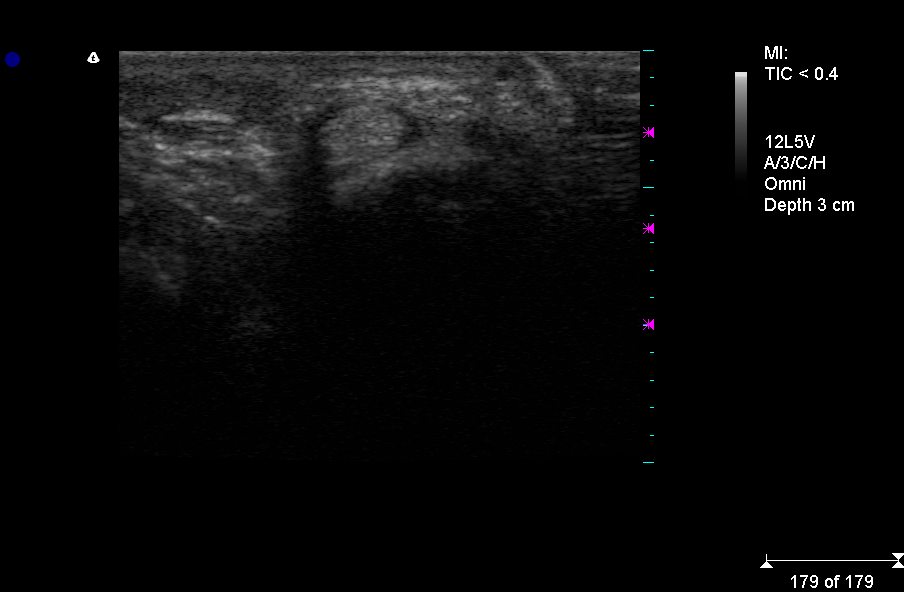

Supplement: S1 Dataset — (ZIP) [file pone.0187042.s001.zip › SegmentationData/HCM12723295_1.bmp]

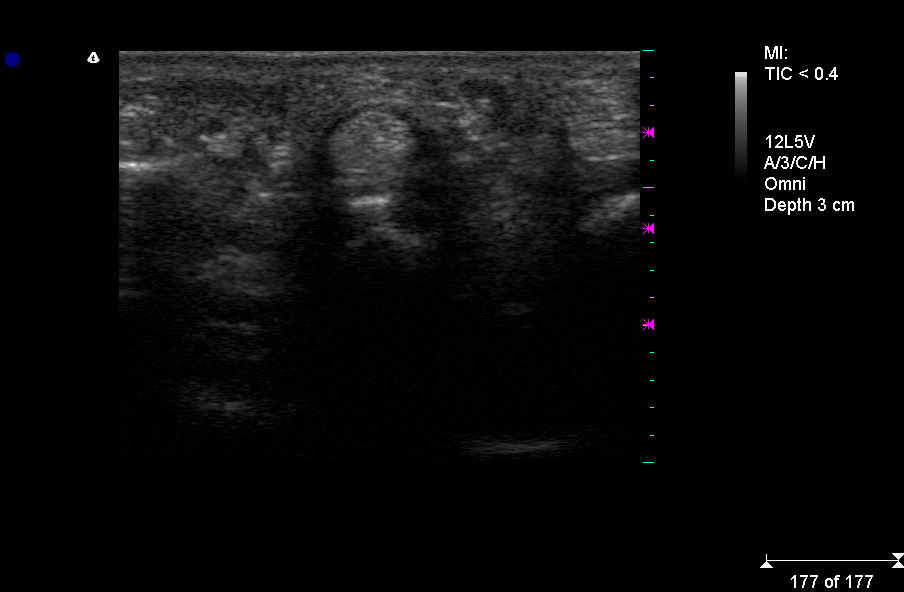

Supplement: S1 Dataset — (ZIP) [file pone.0187042.s001.zip › SegmentationData/HCM12723295_2.bmp]

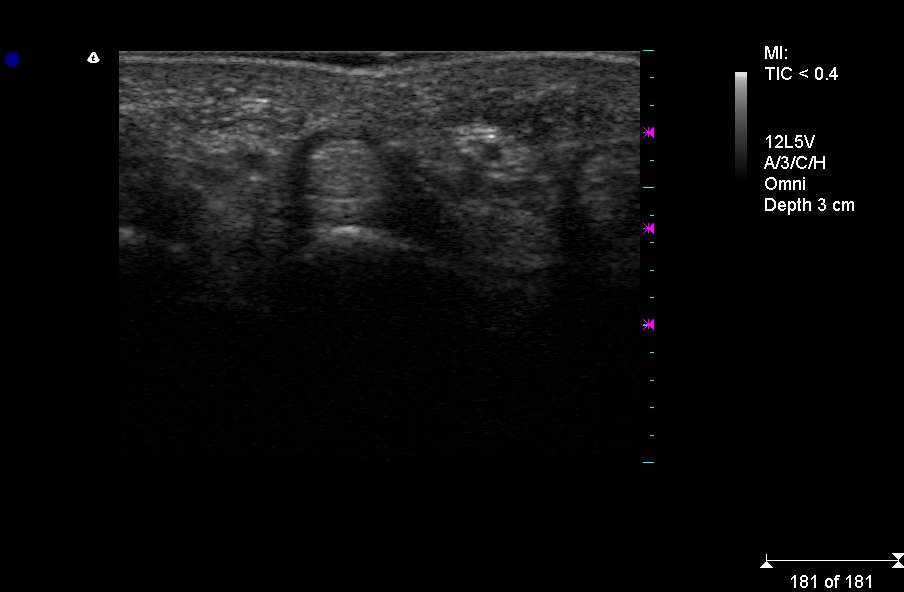

Supplement: S1 Dataset — (ZIP) [file pone.0187042.s001.zip › SegmentationData/HLS998952.bmp]

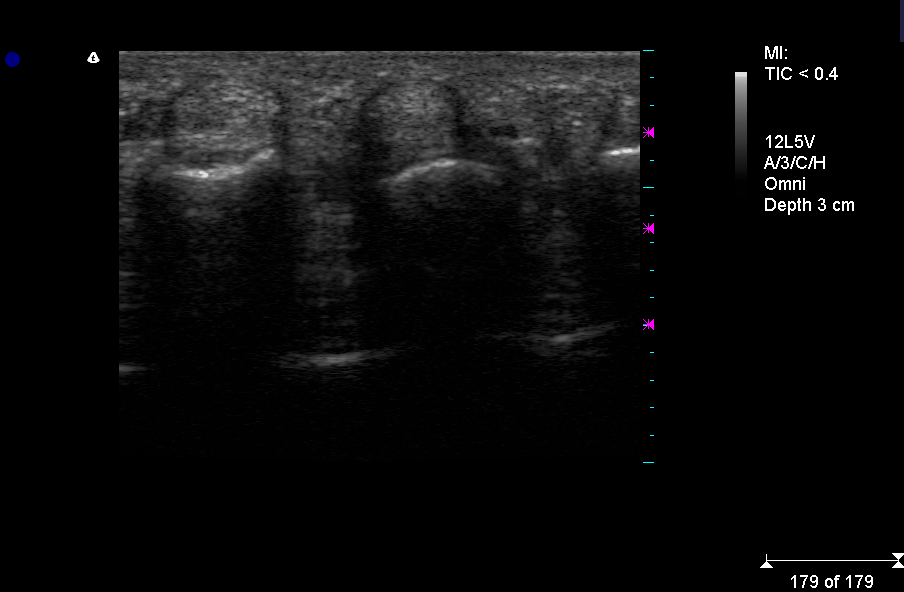

Supplement: S1 Dataset — (ZIP) [file pone.0187042.s001.zip › SegmentationData/KHSZ26402.bmp]

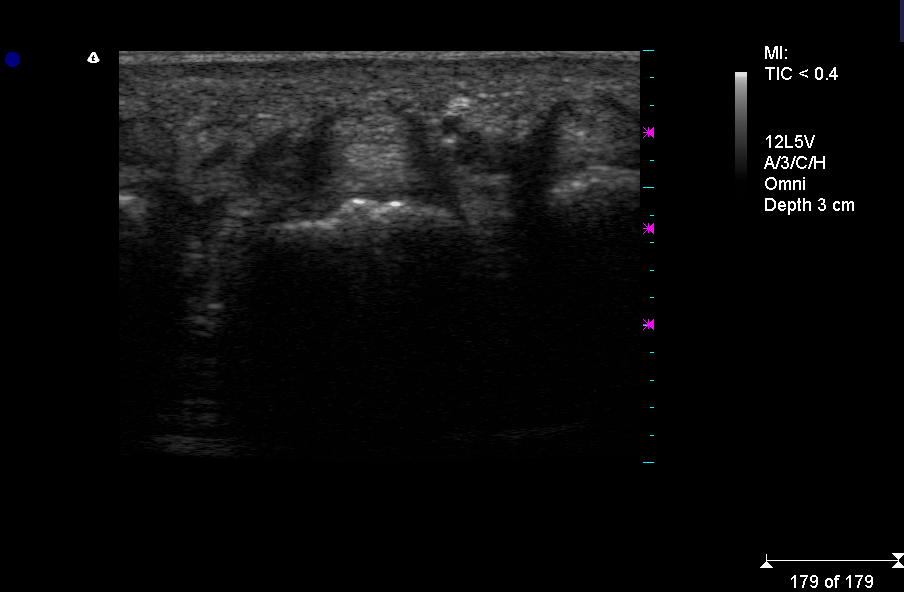

Supplement: S1 Dataset — (ZIP) [file pone.0187042.s001.zip › SegmentationData/KL874488_1.bmp]

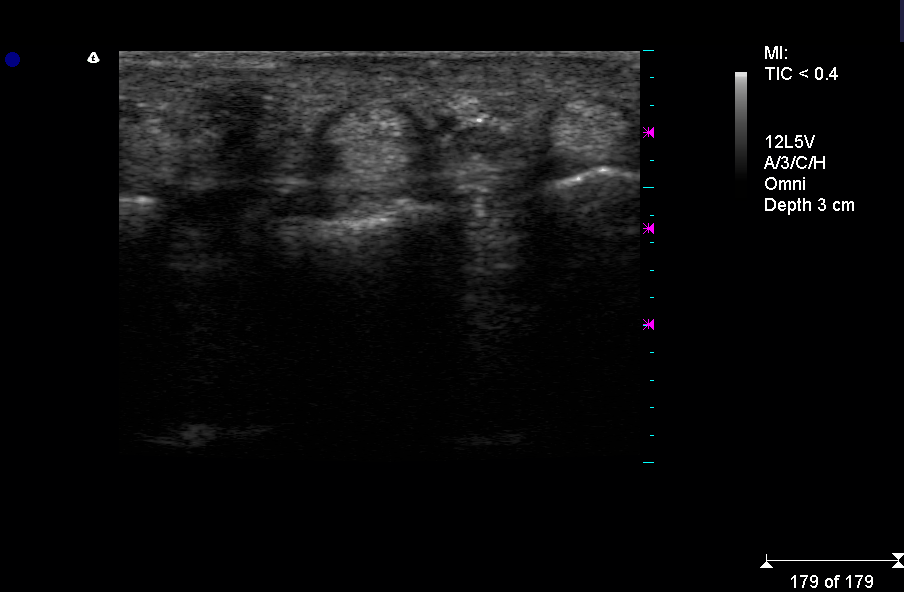

Supplement: S1 Dataset — (ZIP) [file pone.0187042.s001.zip › SegmentationData/KL874488_2.bmp]

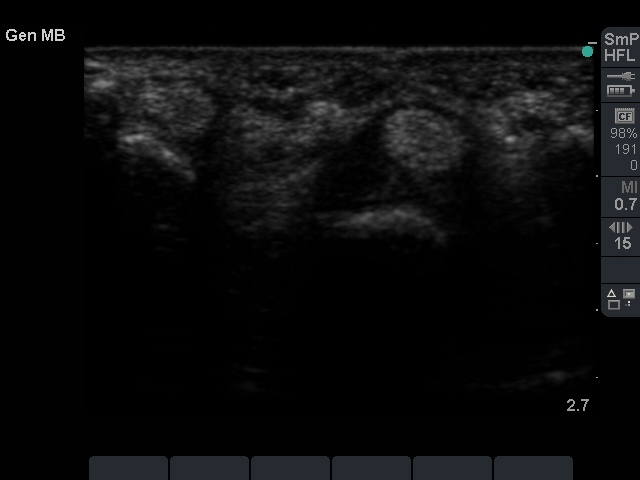

Supplement: S1 Dataset — (ZIP) [file pone.0187042.s001.zip › SegmentationData/L2T1.bmp]

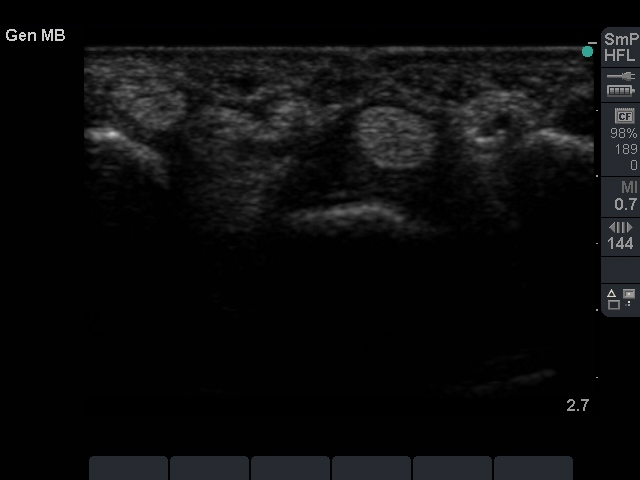

Supplement: S1 Dataset — (ZIP) [file pone.0187042.s001.zip › SegmentationData/L2T3.bmp]

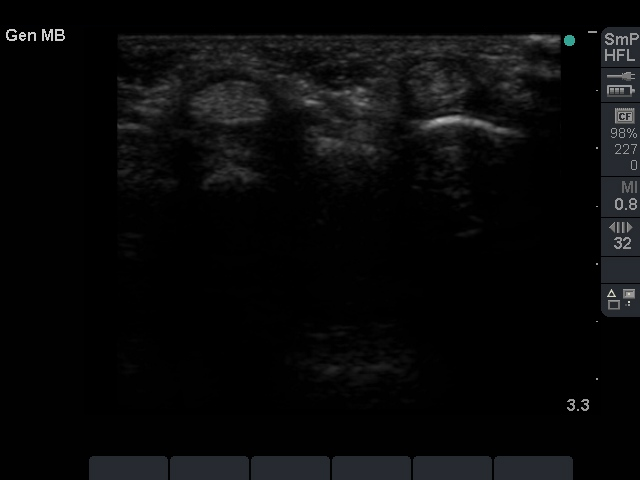

Supplement: S1 Dataset — (ZIP) [file pone.0187042.s001.zip › SegmentationData/L2T31.bmp]

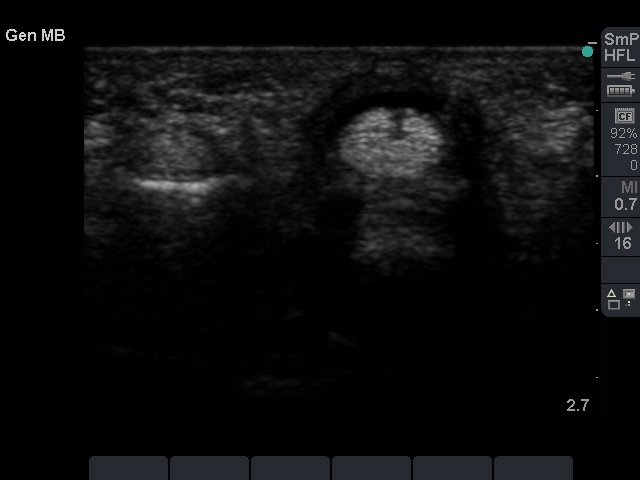

Supplement: S1 Dataset — (ZIP) [file pone.0187042.s001.zip › SegmentationData/L3T3.bmp]

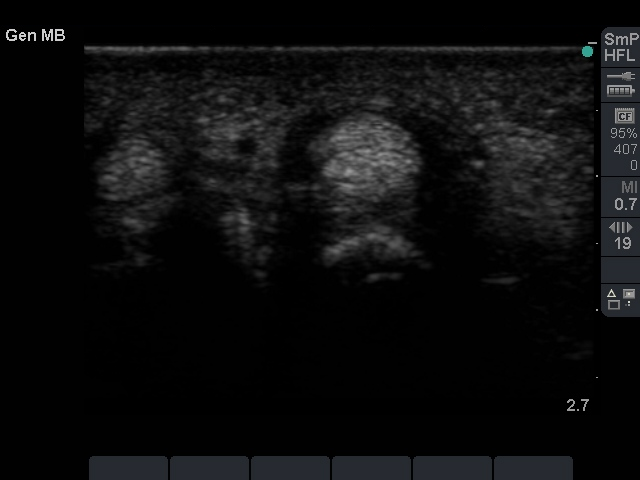

Supplement: S1 Dataset — (ZIP) [file pone.0187042.s001.zip › SegmentationData/L46.bmp]

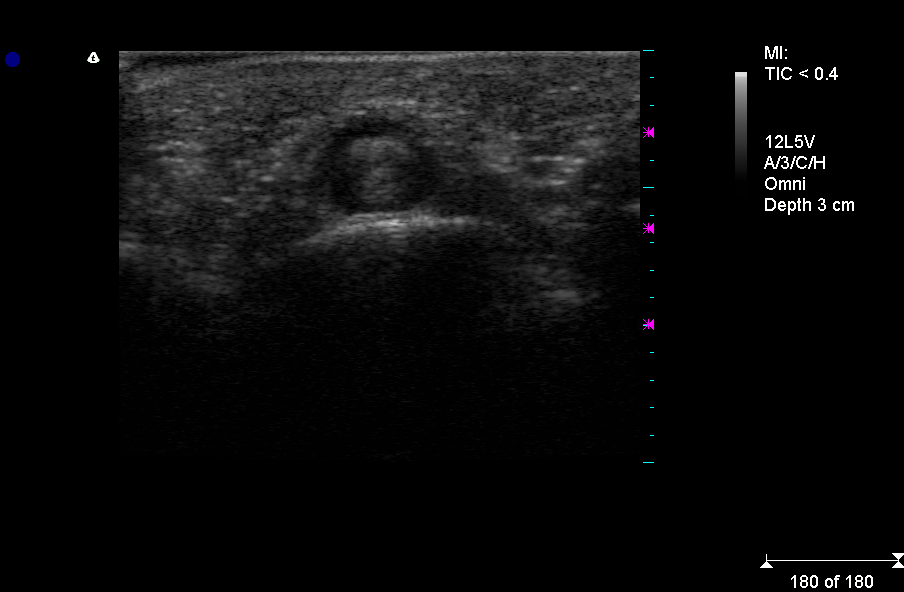

Supplement: S1 Dataset — (ZIP) [file pone.0187042.s001.zip › SegmentationData/LMC14287212.bmp]

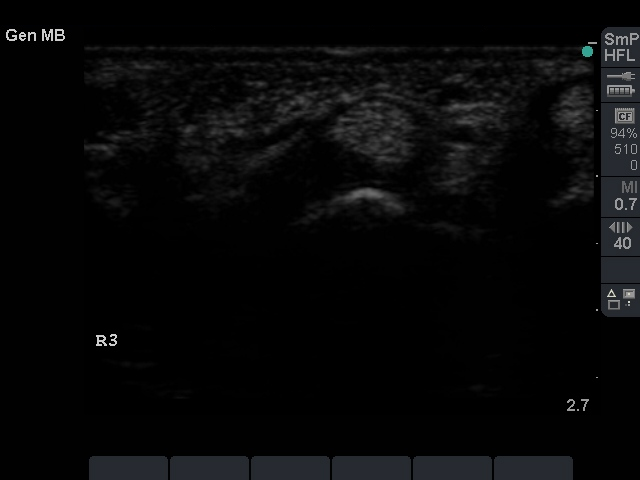

Supplement: S1 Dataset — (ZIP) [file pone.0187042.s001.zip › SegmentationData/R3T1.bmp]

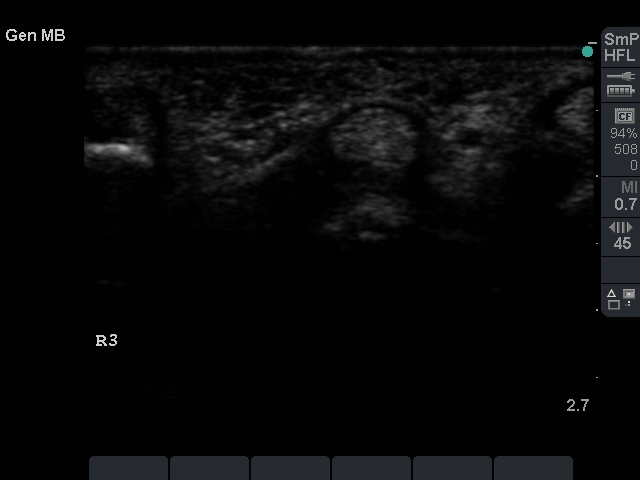

Supplement: S1 Dataset — (ZIP) [file pone.0187042.s001.zip › SegmentationData/R3T3.bmp]

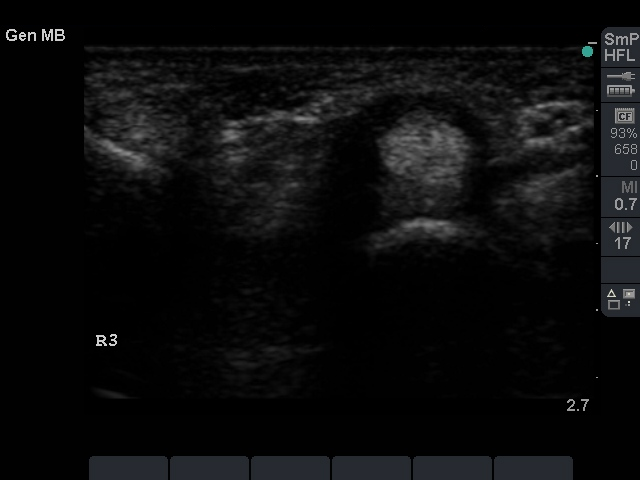

Supplement: S1 Dataset — (ZIP) [file pone.0187042.s001.zip › SegmentationData/R3T32.bmp]

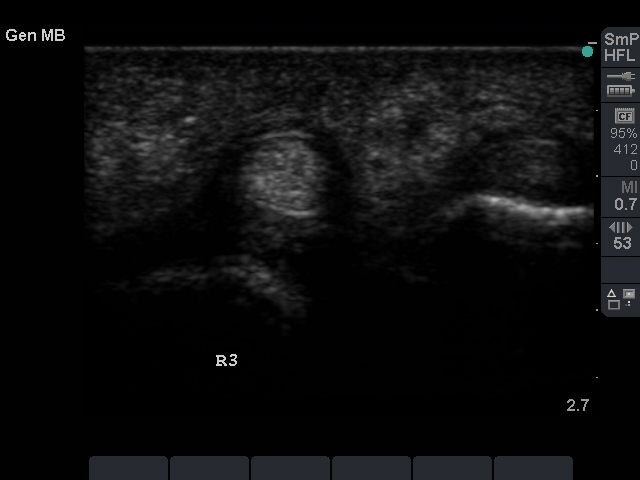

Supplement: S1 Dataset — (ZIP) [file pone.0187042.s001.zip › SegmentationData/R46.bmp]

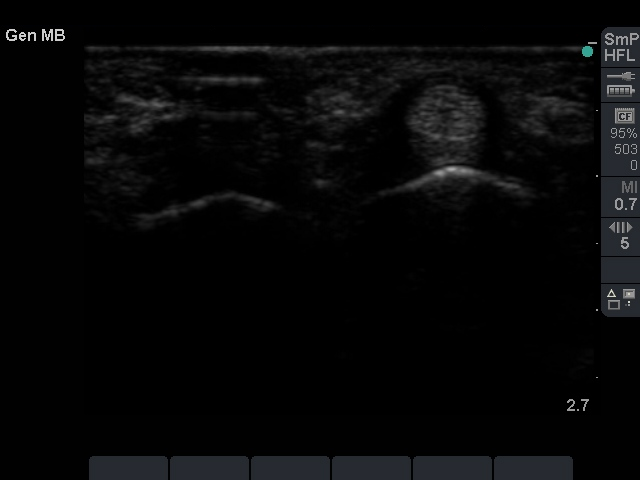

Supplement: S1 Dataset — (ZIP) [file pone.0187042.s001.zip › SegmentationData/R4T1.bmp]

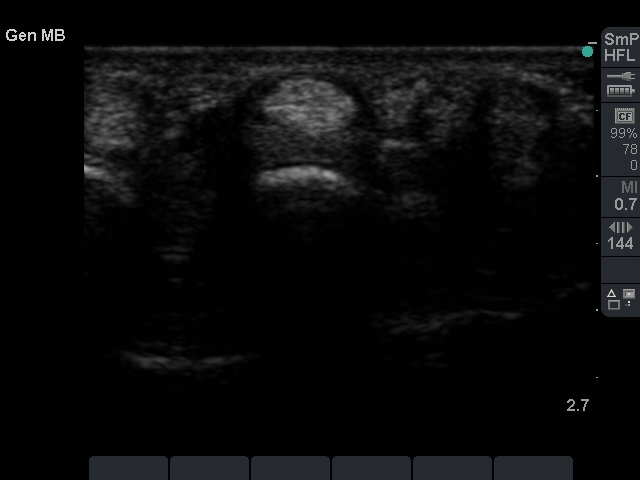

Supplement: S1 Dataset — (ZIP) [file pone.0187042.s001.zip › SegmentationData/R4T3.bmp]

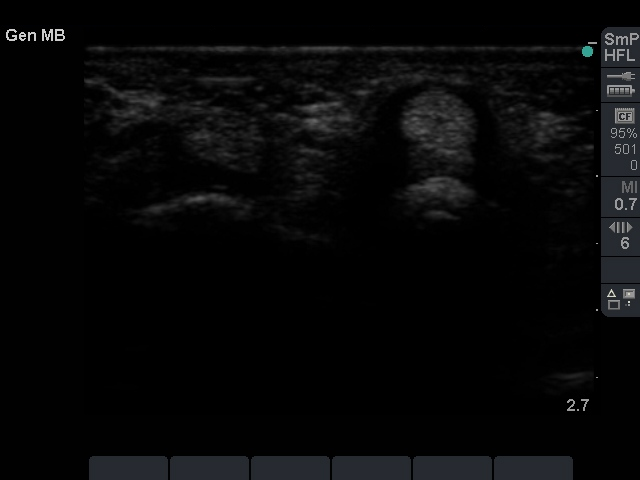

Supplement: S1 Dataset — (ZIP) [file pone.0187042.s001.zip › SegmentationData/R4T32.bmp]

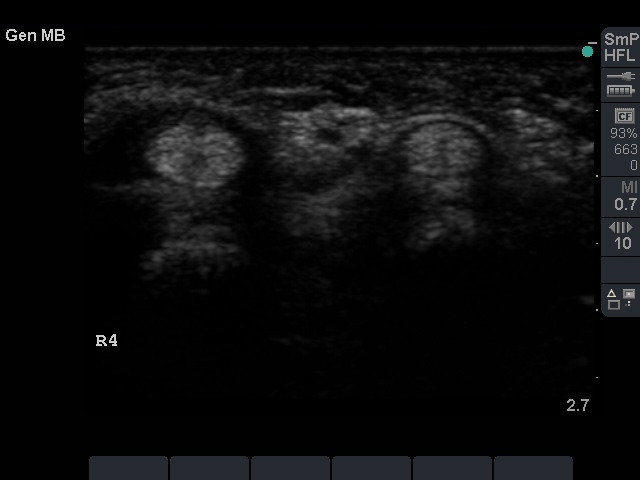

Supplement: S1 Dataset — (ZIP) [file pone.0187042.s001.zip › SegmentationData/R4T3_1.bmp]

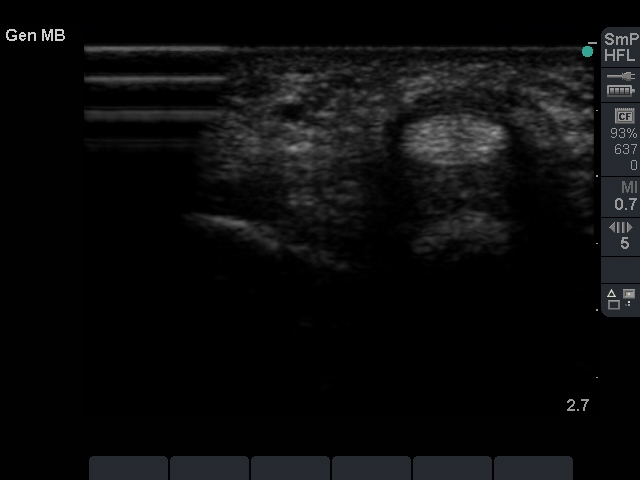

Supplement: S1 Dataset — (ZIP) [file pone.0187042.s001.zip › SegmentationData/R4T3_2.bmp]

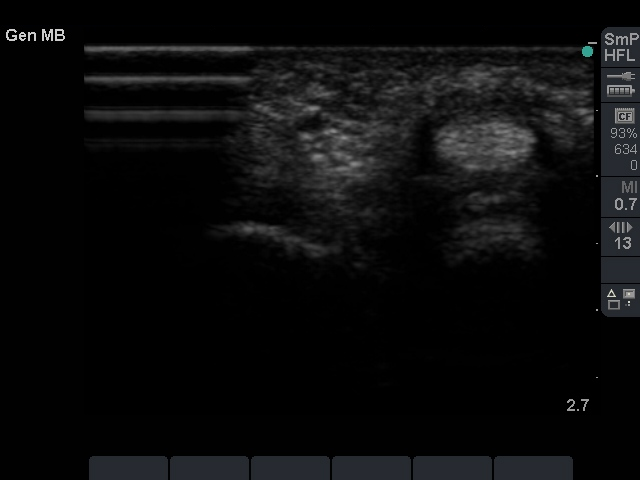

Supplement: S1 Dataset — (ZIP) [file pone.0187042.s001.zip › SegmentationData/RT32.bmp]

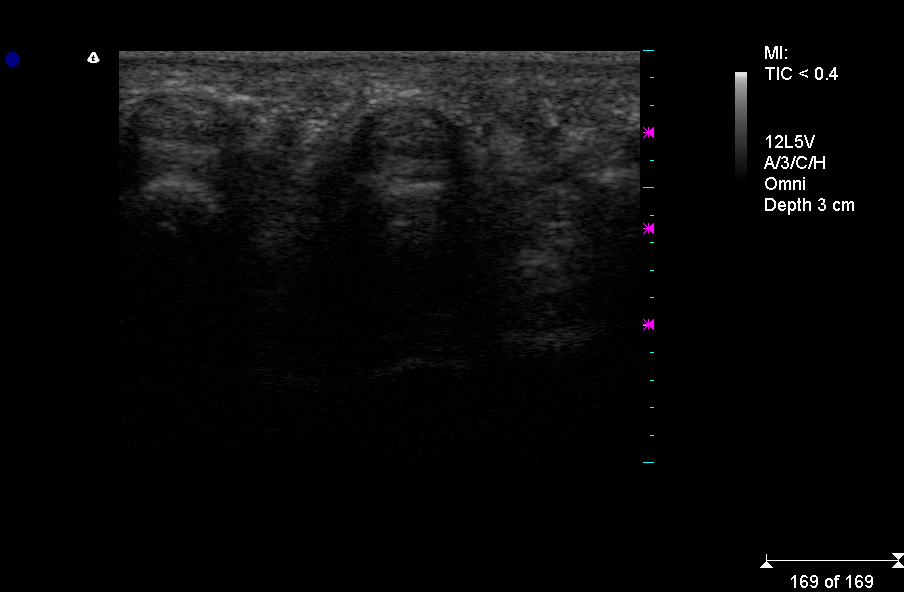

Supplement: S1 Dataset — (ZIP) [file pone.0187042.s001.zip › SegmentationData/WJY691133.bmp]

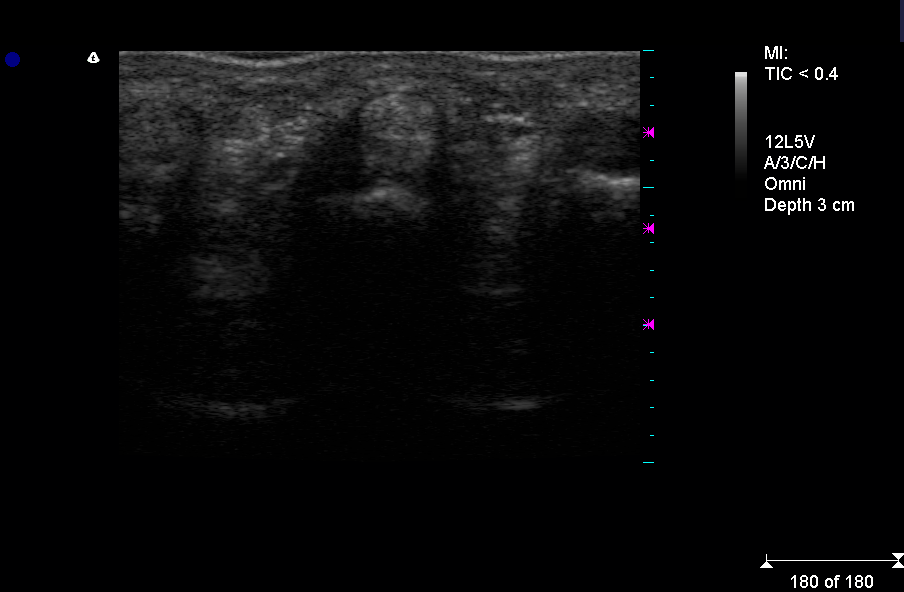

Supplement: S1 Dataset — (ZIP) [file pone.0187042.s001.zip › SegmentationData/WSH5043624_2.bmp]

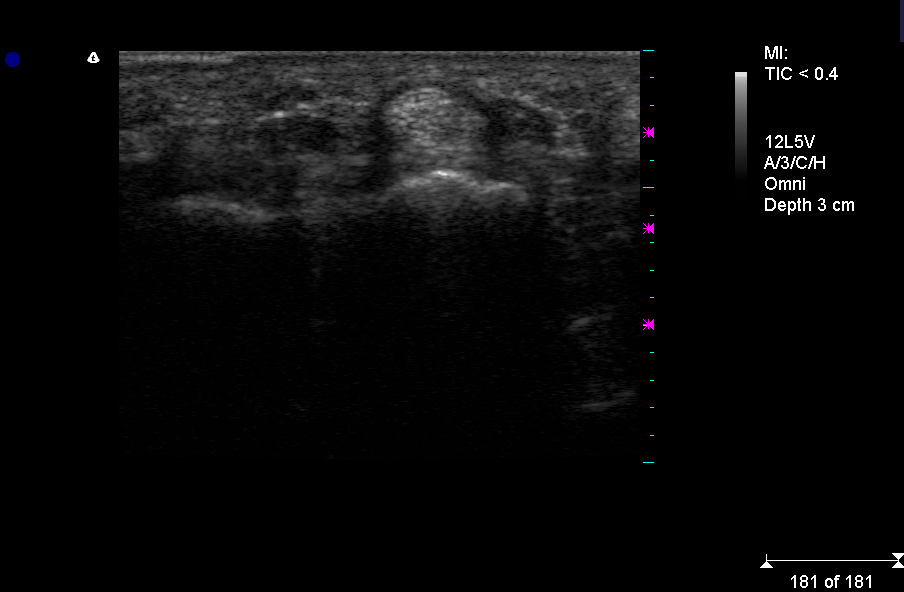

Supplement: S1 Dataset — (ZIP) [file pone.0187042.s001.zip › SegmentationData/ZCH5592493.bmp]

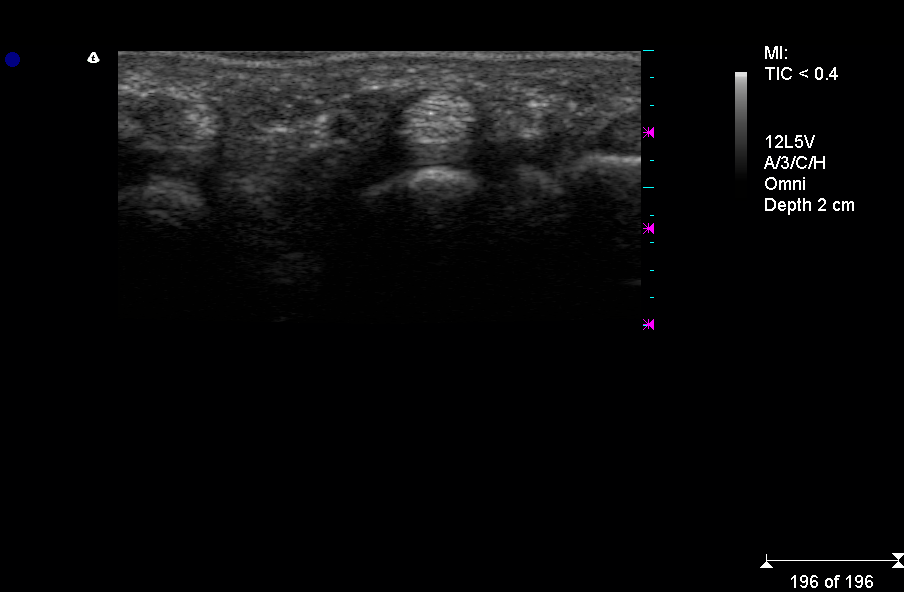

Supplement: S3 Dataset — (ZIP) [file pone.0187042.s003.zip › Classification Data/Normal/Left Hand/AS_1_L.bmp]

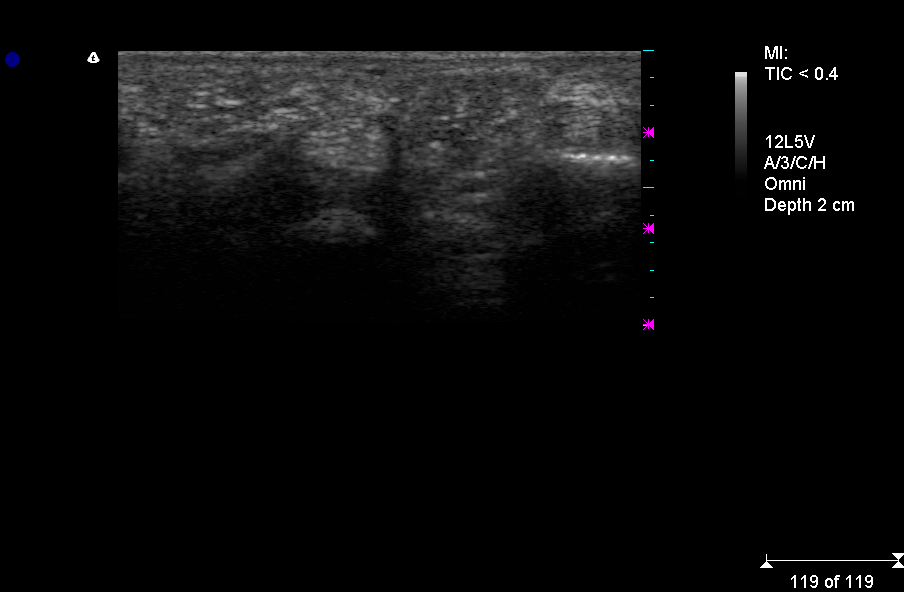

Supplement: S3 Dataset — (ZIP) [file pone.0187042.s003.zip › Classification Data/Normal/Left Hand/MD_10_L.bmp]

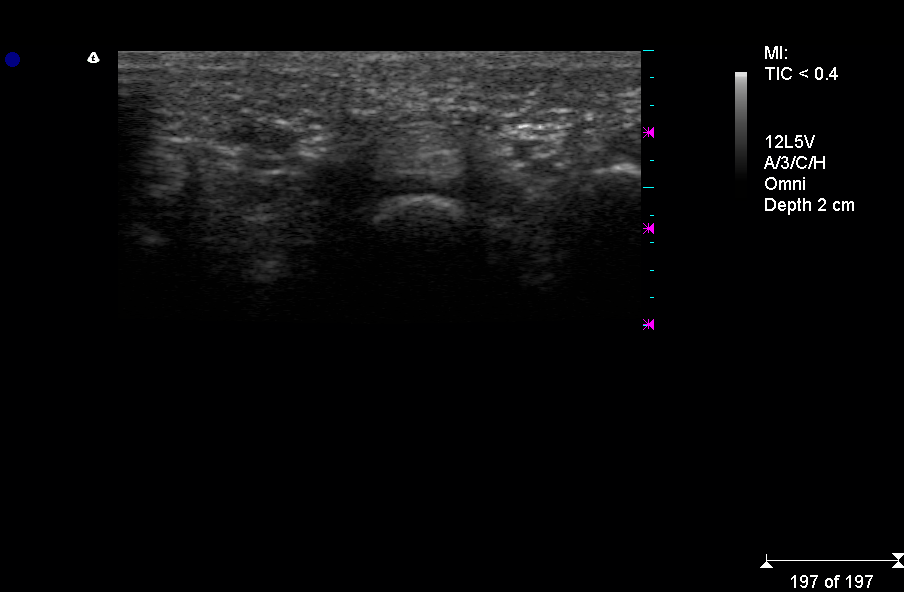

Supplement: S3 Dataset — (ZIP) [file pone.0187042.s003.zip › Classification Data/Normal/Left Hand/MD_11_L.bmp]

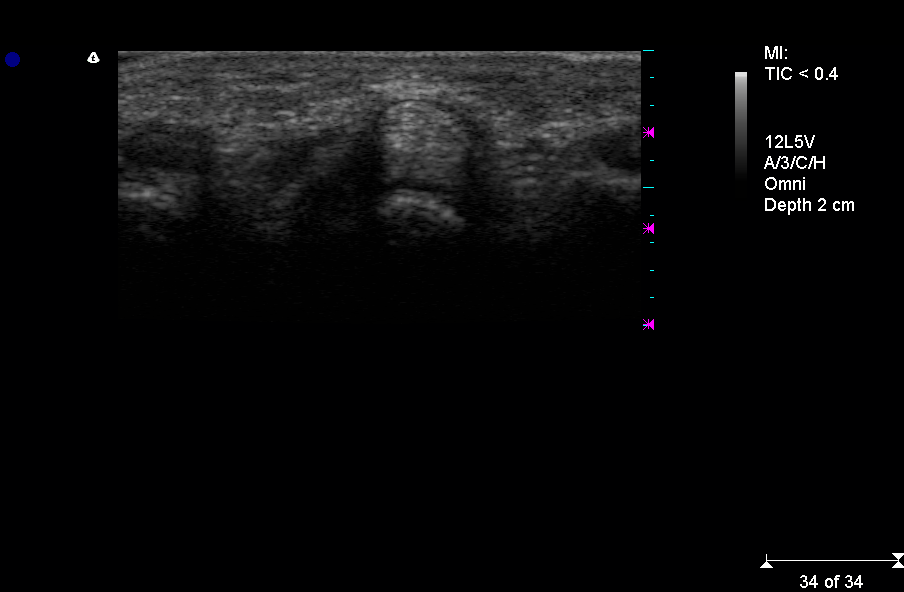

Supplement: S3 Dataset — (ZIP) [file pone.0187042.s003.zip › Classification Data/Normal/Left Hand/MD_12_L.bmp]

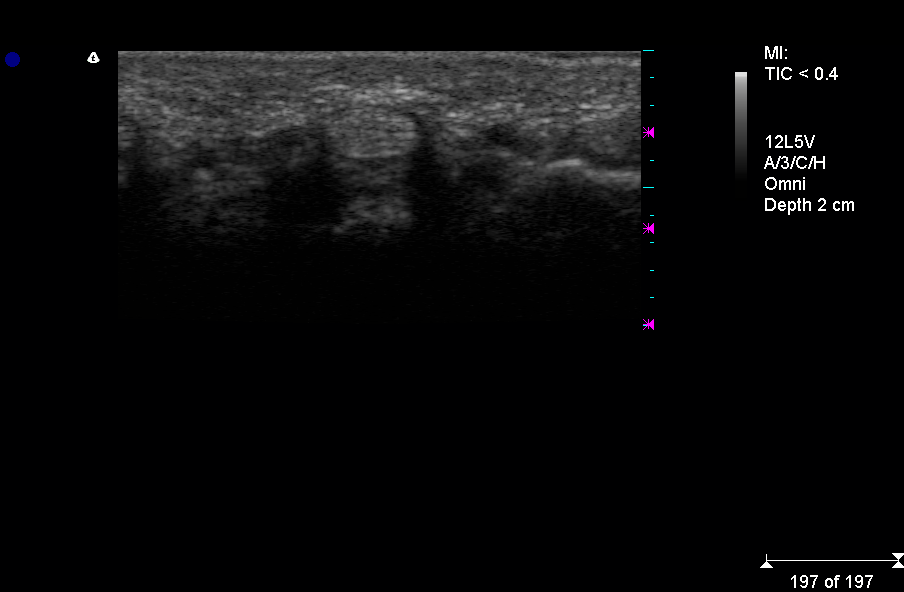

Supplement: S3 Dataset — (ZIP) [file pone.0187042.s003.zip › Classification Data/Normal/Left Hand/MD_13_L.bmp]

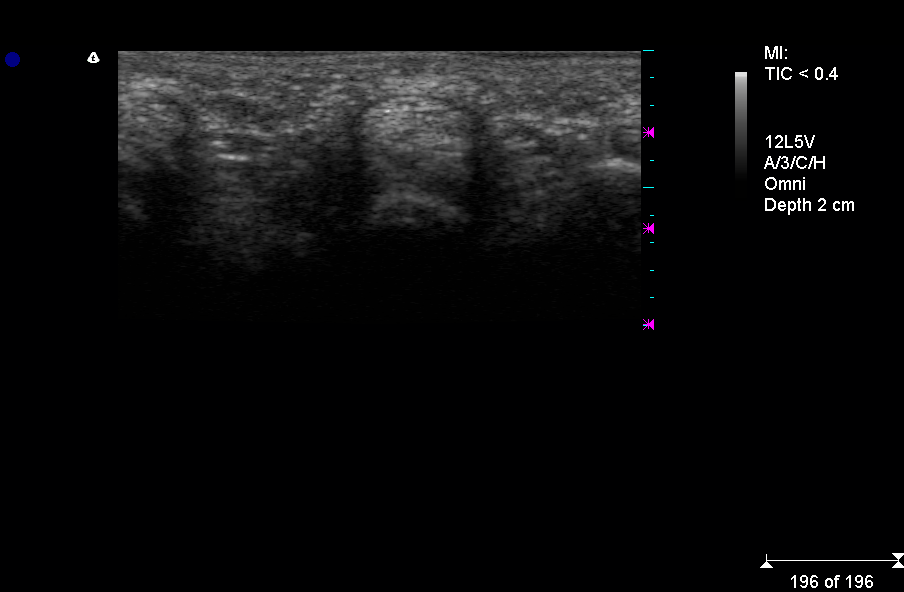

Supplement: S3 Dataset — (ZIP) [file pone.0187042.s003.zip › Classification Data/Normal/Left Hand/MD_14_L.bmp]

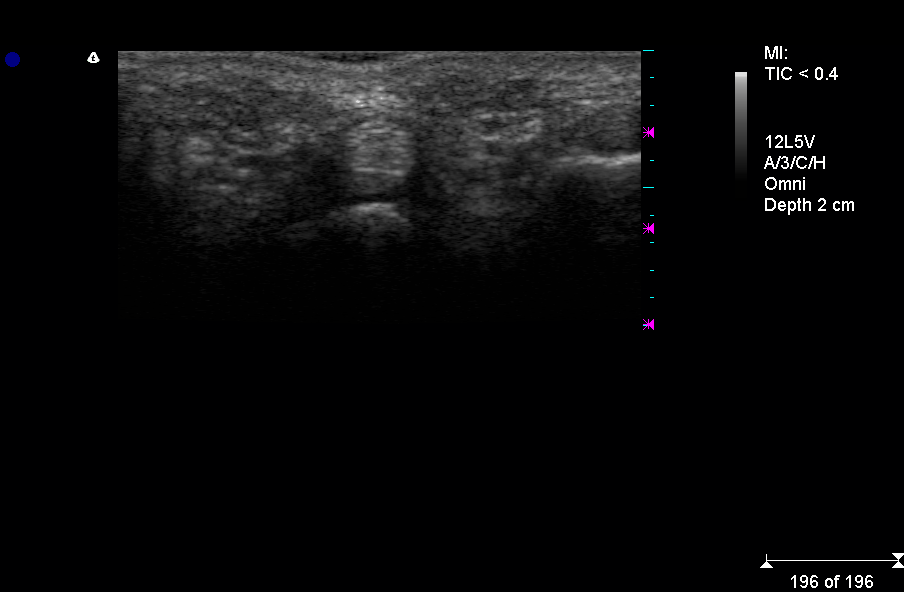

Supplement: S3 Dataset — (ZIP) [file pone.0187042.s003.zip › Classification Data/Normal/Left Hand/MD_15_L.bmp]

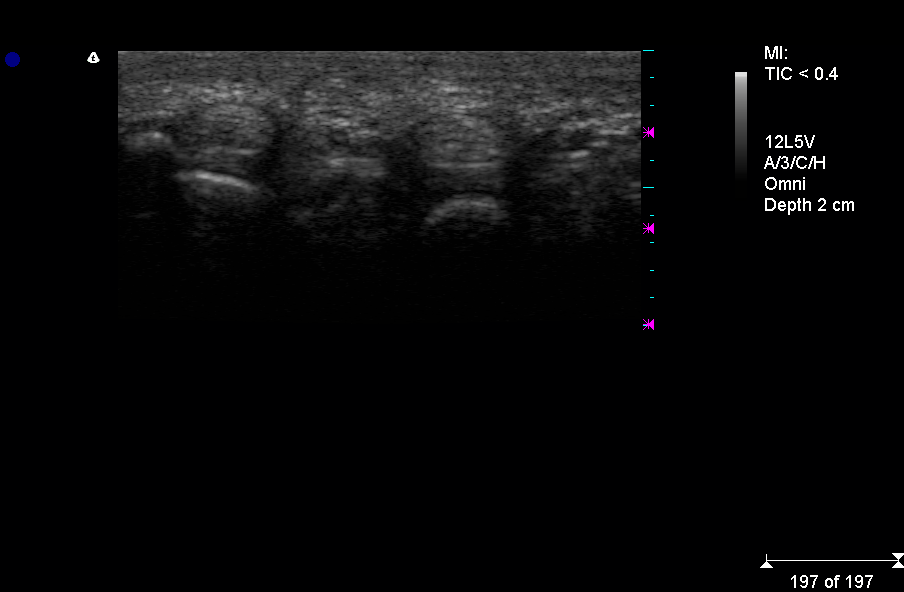

Supplement: S3 Dataset — (ZIP) [file pone.0187042.s003.zip › Classification Data/Normal/Left Hand/MD_1_L.bmp]

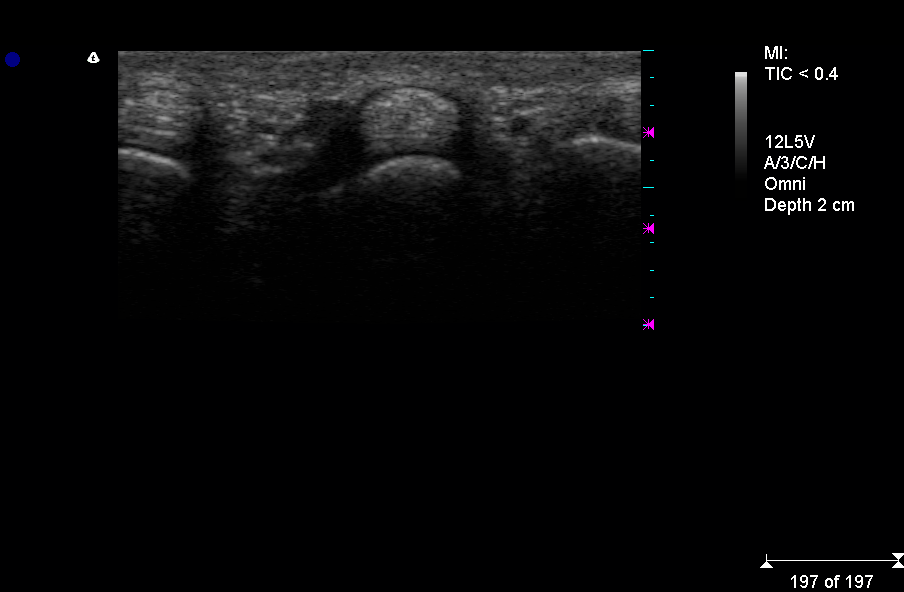

Supplement: S3 Dataset — (ZIP) [file pone.0187042.s003.zip › Classification Data/Normal/Left Hand/MD_2_L.bmp]

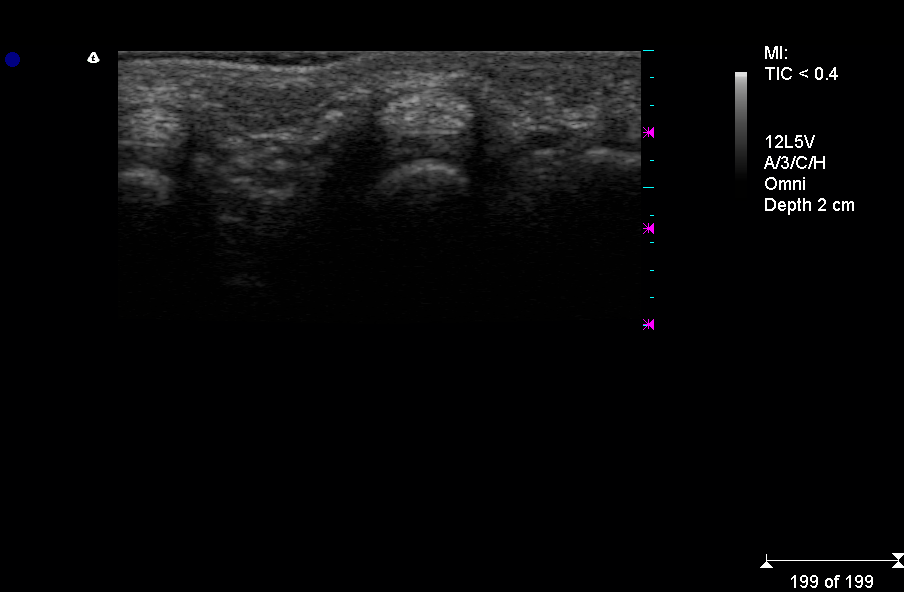

Supplement: S3 Dataset — (ZIP) [file pone.0187042.s003.zip › Classification Data/Normal/Left Hand/MD_3_L.bmp]

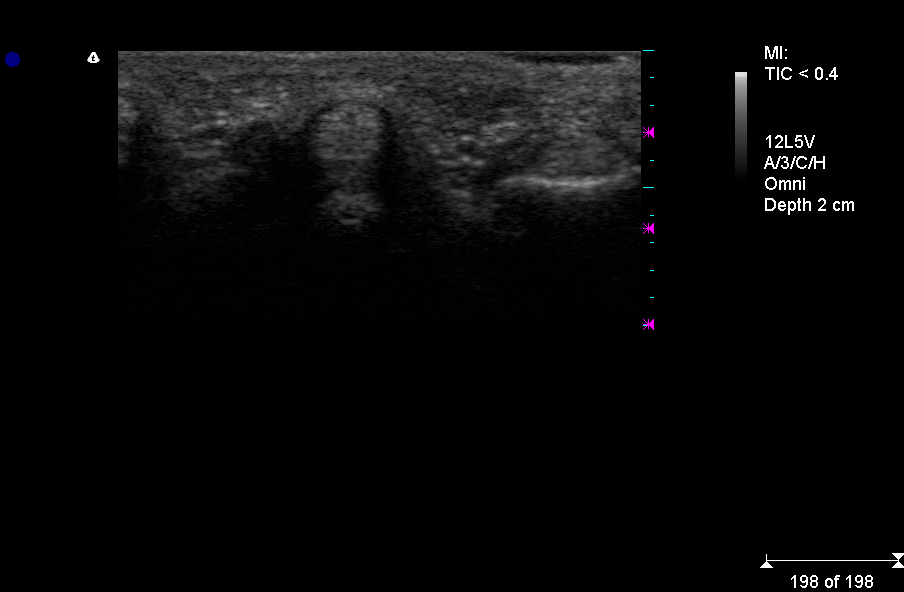

Supplement: S3 Dataset — (ZIP) [file pone.0187042.s003.zip › Classification Data/Normal/Left Hand/MD_4_L.bmp]

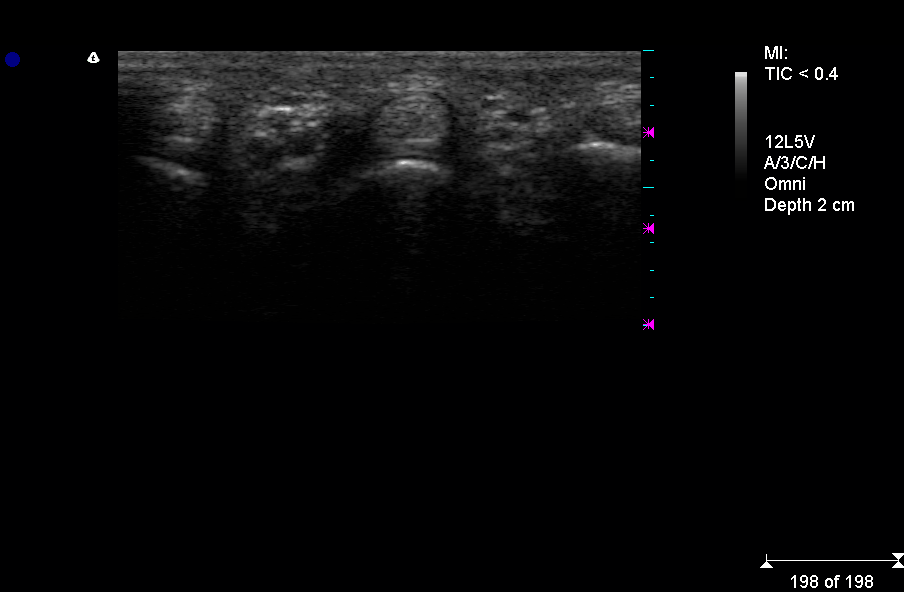

Supplement: S3 Dataset — (ZIP) [file pone.0187042.s003.zip › Classification Data/Normal/Left Hand/MD_5_L.bmp]

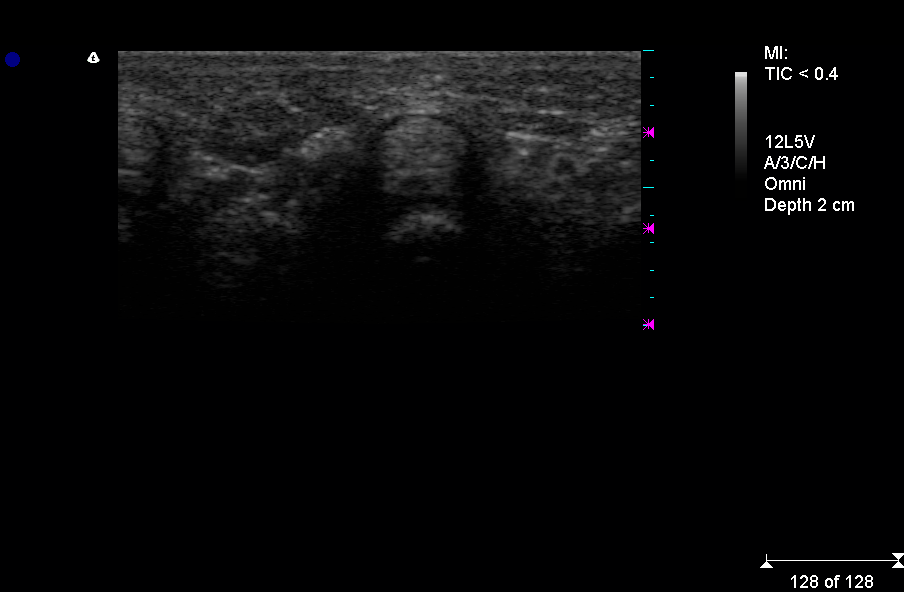

Supplement: S3 Dataset — (ZIP) [file pone.0187042.s003.zip › Classification Data/Normal/Left Hand/MD_6_L.bmp]

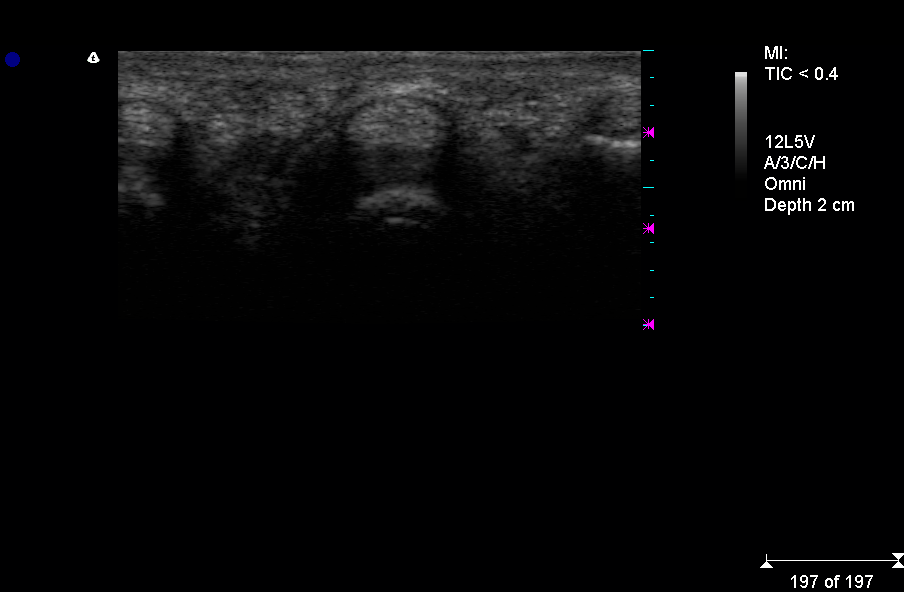

Supplement: S3 Dataset — (ZIP) [file pone.0187042.s003.zip › Classification Data/Normal/Left Hand/MD_7_L.bmp]

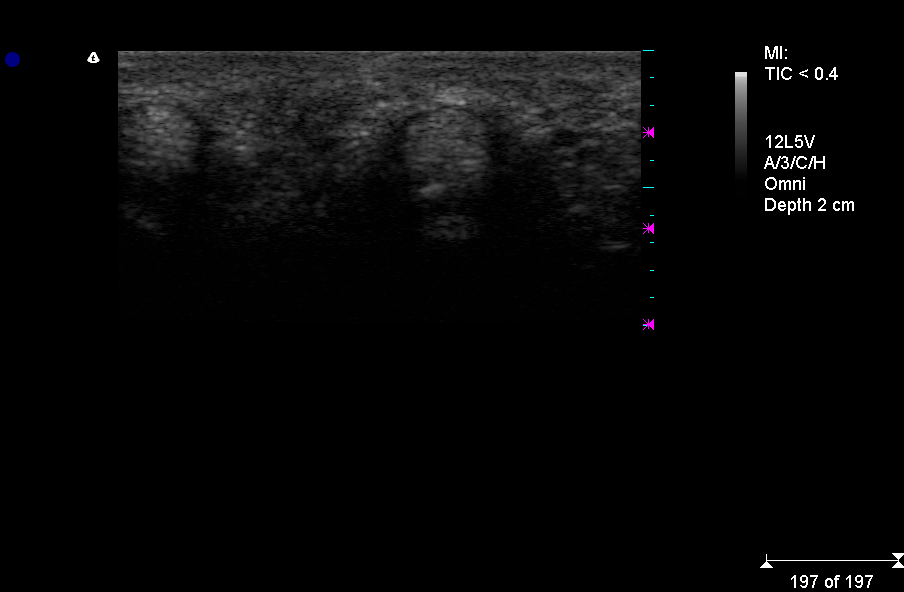

Supplement: S3 Dataset — (ZIP) [file pone.0187042.s003.zip › Classification Data/Normal/Left Hand/MD_8_L.bmp]

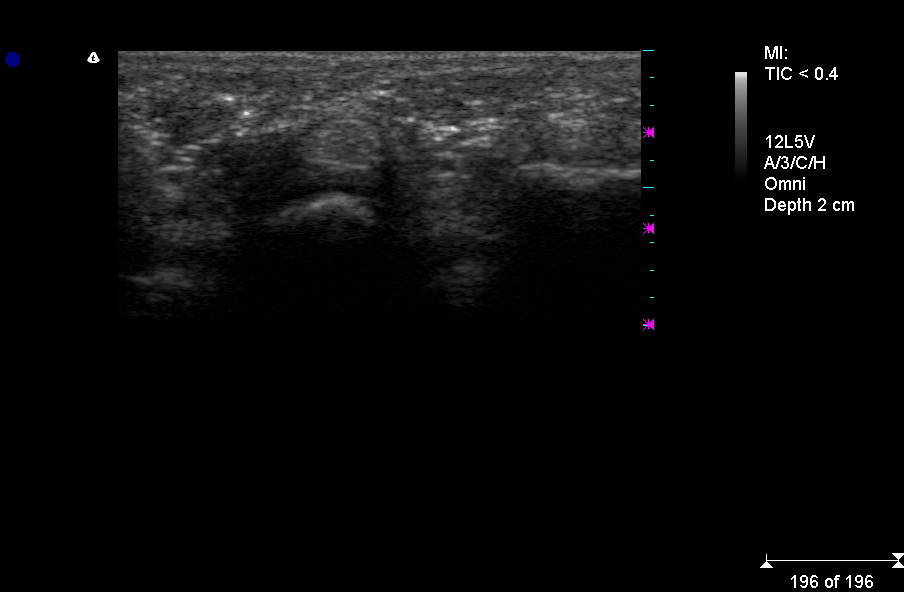

Supplement: S3 Dataset — (ZIP) [file pone.0187042.s003.zip › Classification Data/Normal/Left Hand/MD_9_L.bmp]

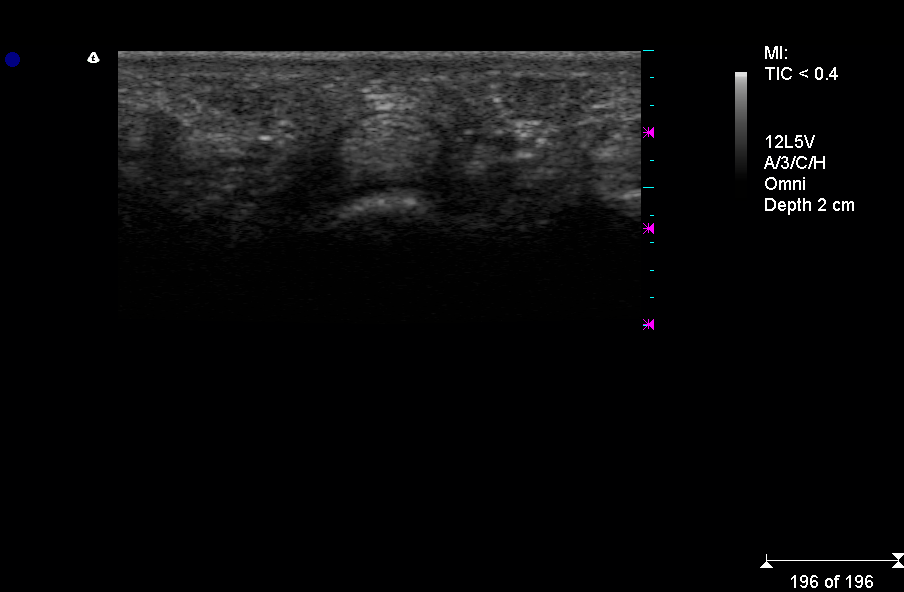

Supplement: S3 Dataset — (ZIP) [file pone.0187042.s003.zip › Classification Data/Normal/Left Hand/PD_1_L.bmp]

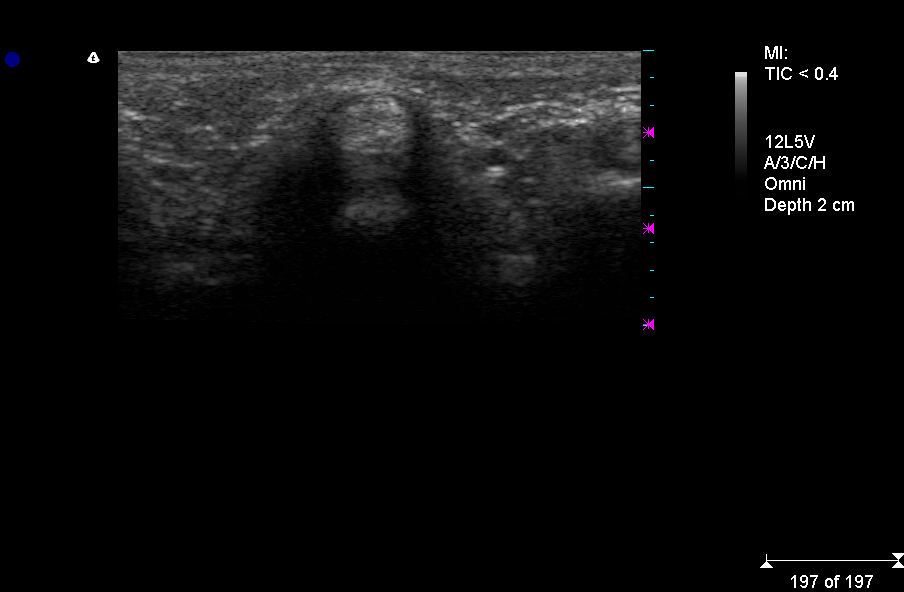

Supplement: S3 Dataset — (ZIP) [file pone.0187042.s003.zip › Classification Data/Normal/Left Hand/phD_1_L.bmp]

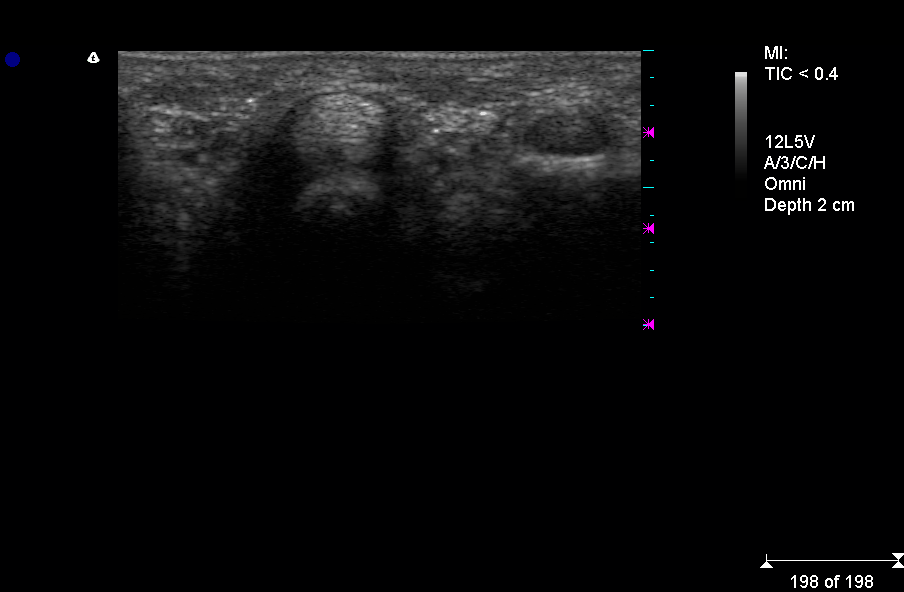

Supplement: S3 Dataset — (ZIP) [file pone.0187042.s003.zip › Classification Data/Normal/Left Hand/phD_2_L.bmp]

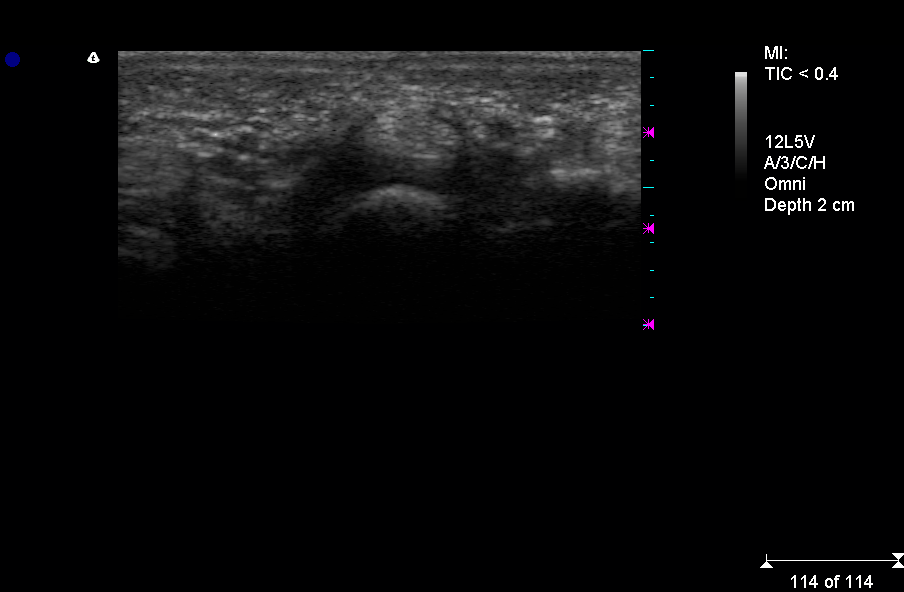

Supplement: S3 Dataset — (ZIP) [file pone.0187042.s003.zip › Classification Data/Normal/Left Hand/phD_3_L.bmp]

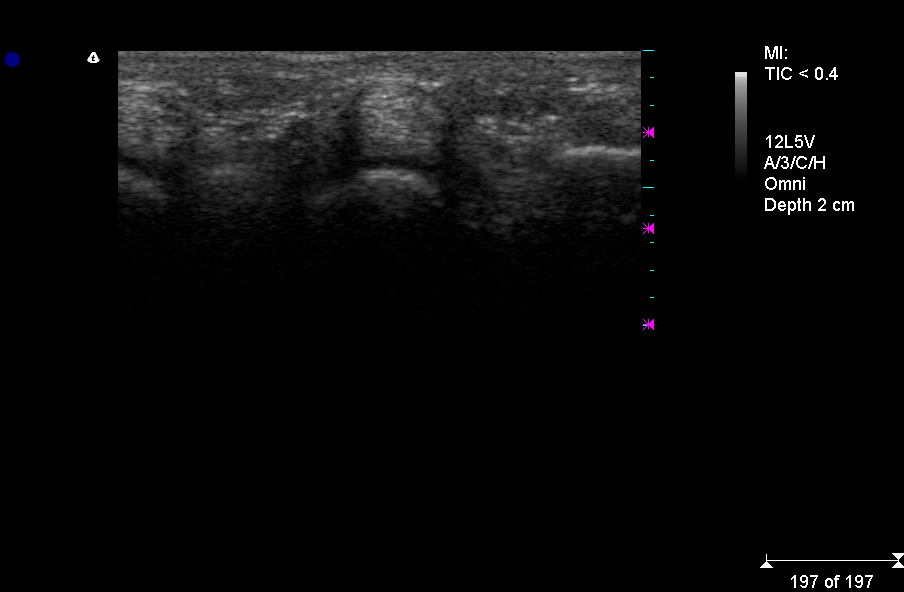

Supplement: S3 Dataset — (ZIP) [file pone.0187042.s003.zip › Classification Data/Normal/Left Hand/phD_4_L.bmp]

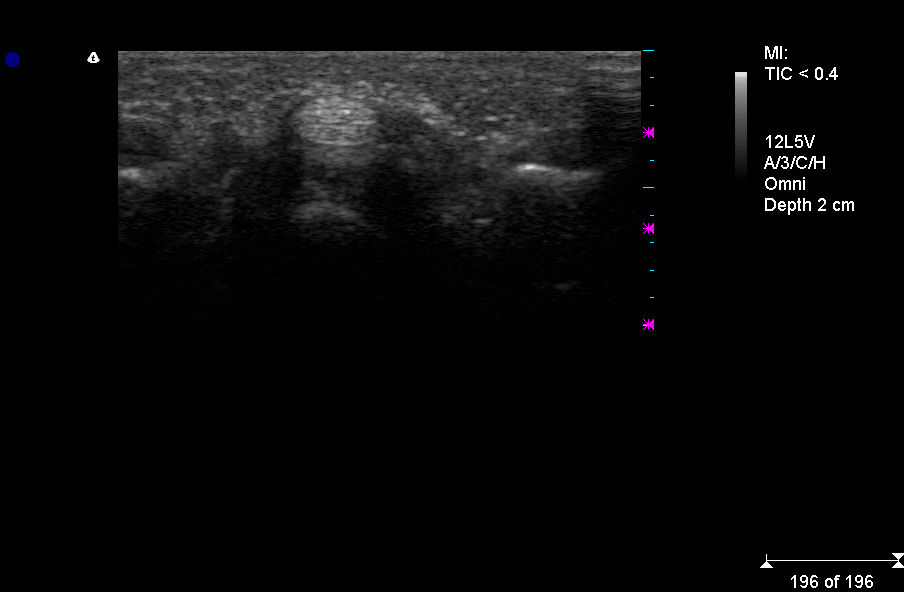

Supplement: S3 Dataset — (ZIP) [file pone.0187042.s003.zip › Classification Data/Normal/Right Hand/AS_1_R.bmp]

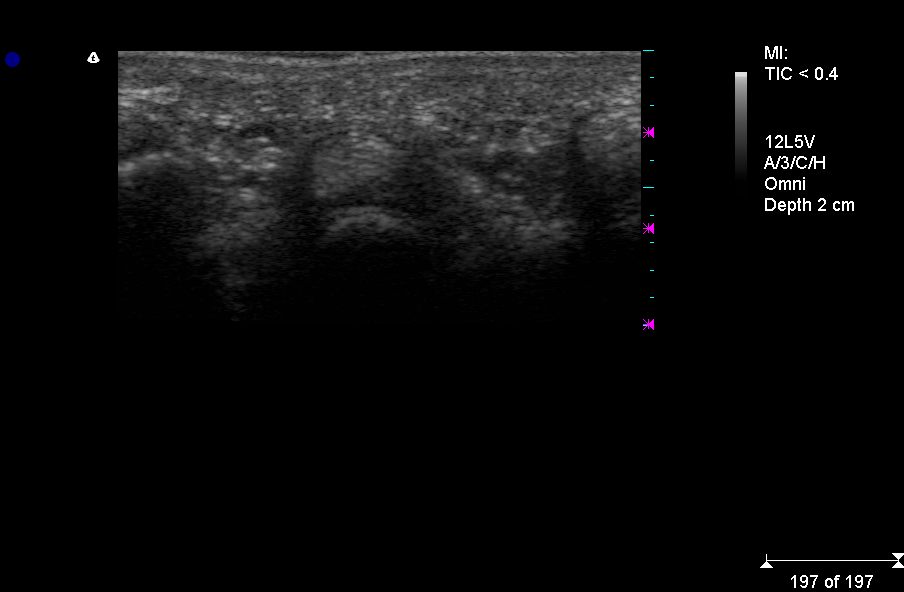

Supplement: S3 Dataset — (ZIP) [file pone.0187042.s003.zip › Classification Data/Normal/Right Hand/MD_10_R.bmp]

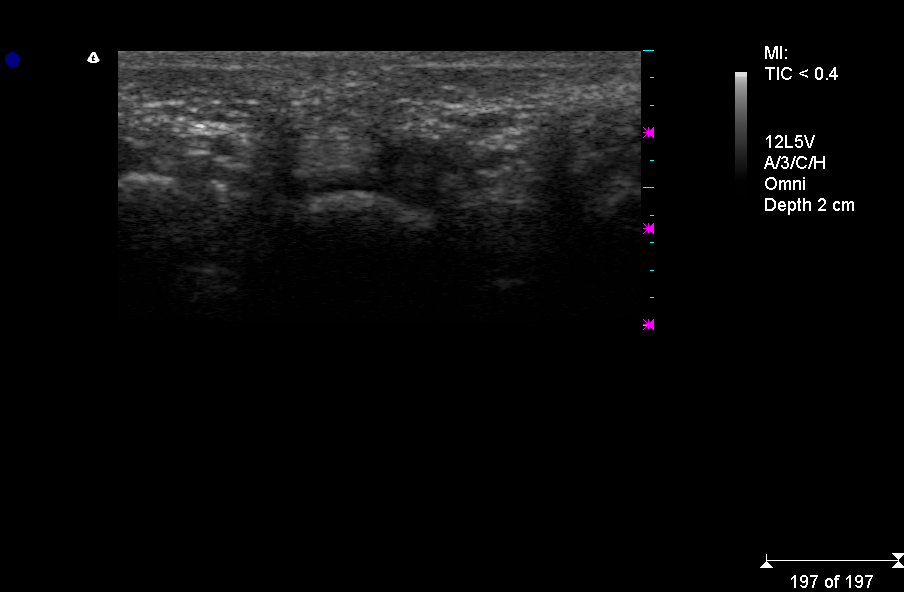

Supplement: S3 Dataset — (ZIP) [file pone.0187042.s003.zip › Classification Data/Normal/Right Hand/MD_11_R.bmp]

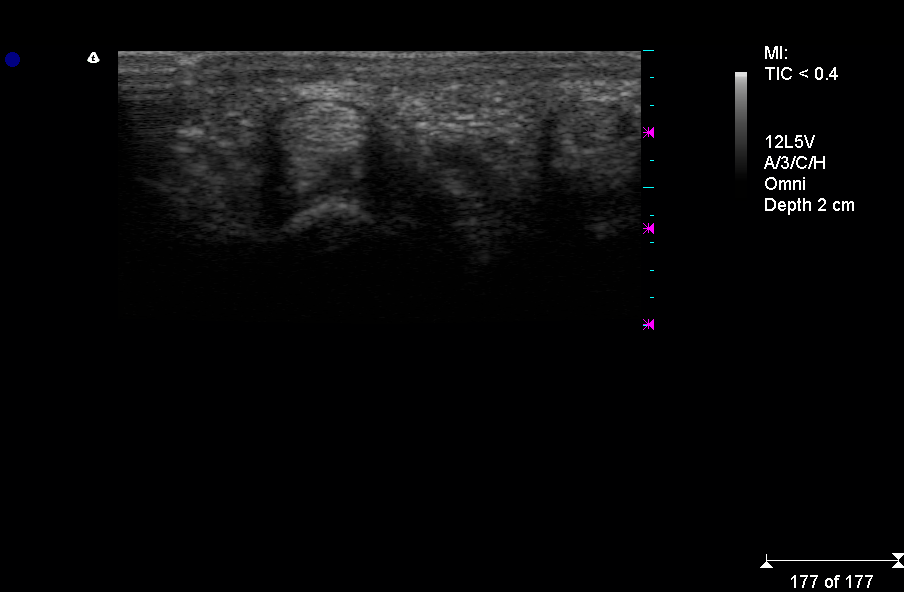

Supplement: S3 Dataset — (ZIP) [file pone.0187042.s003.zip › Classification Data/Normal/Right Hand/MD_12_R.bmp]

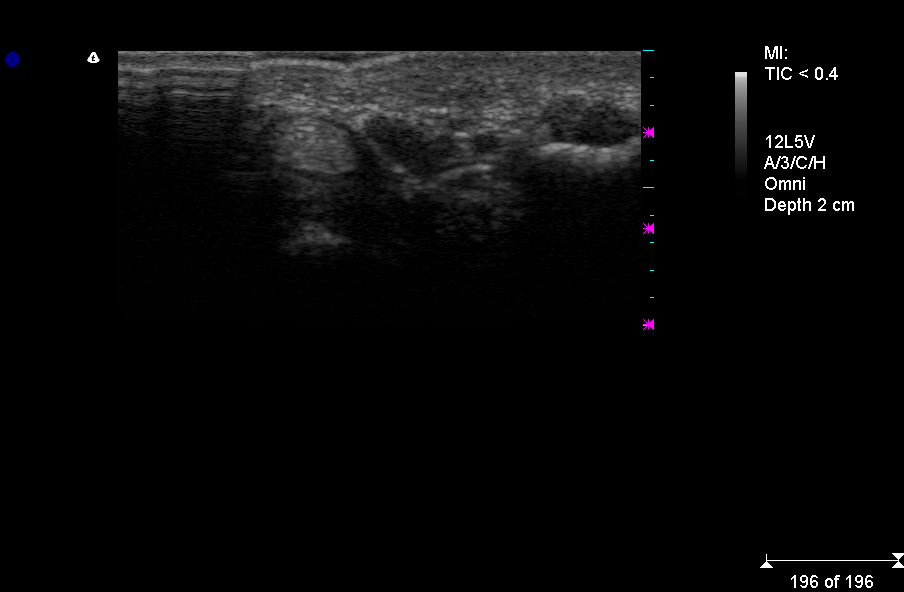

Supplement: S3 Dataset — (ZIP) [file pone.0187042.s003.zip › Classification Data/Normal/Right Hand/MD_13_R.bmp]
