# Supplementary figures and images for: A medical imaging analysis system for trigger finger using an adaptive texture-based active shape model (ATASM) in ultrasound images (part 2 of 2)
Source: PLoS One. 2017 Oct 27;12(10):e0187042. doi: 10.1371/journal.pone.0187042 (PMC5659776; doi:10.1371/journal.pone.0187042)

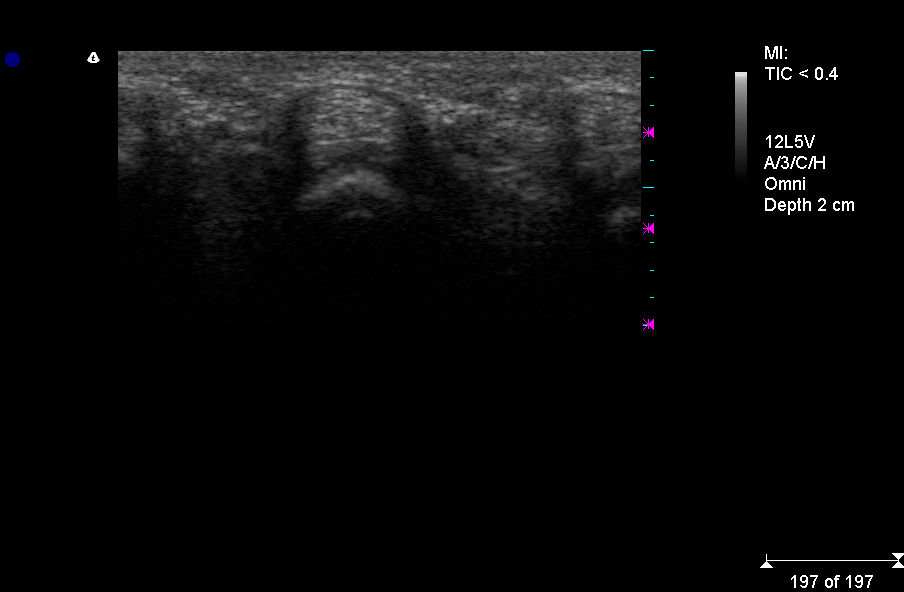

Supplement: S3 Dataset — (ZIP) [file pone.0187042.s003.zip › Classification Data/Normal/Right Hand/MD_14_R.bmp]

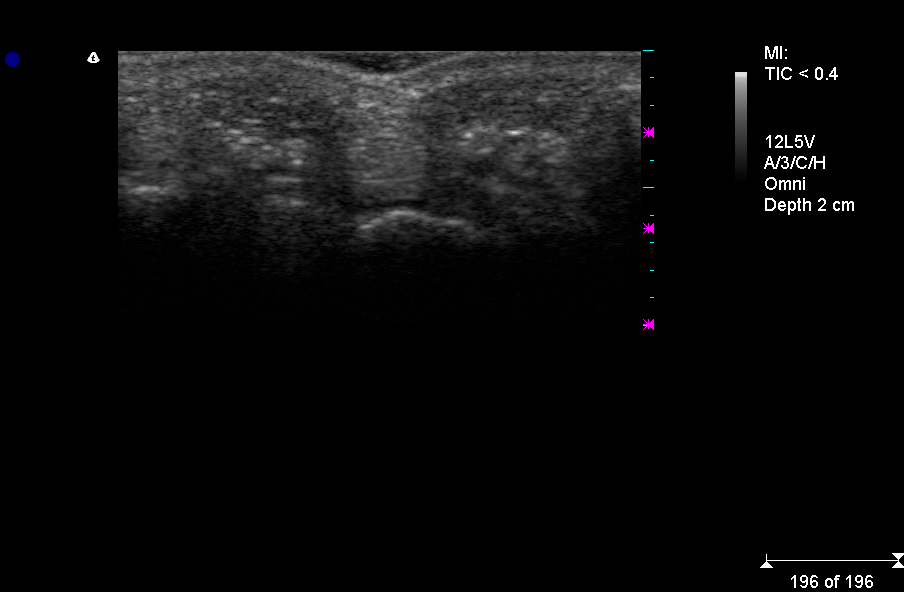

Supplement: S3 Dataset — (ZIP) [file pone.0187042.s003.zip › Classification Data/Normal/Right Hand/MD_15_R.bmp]

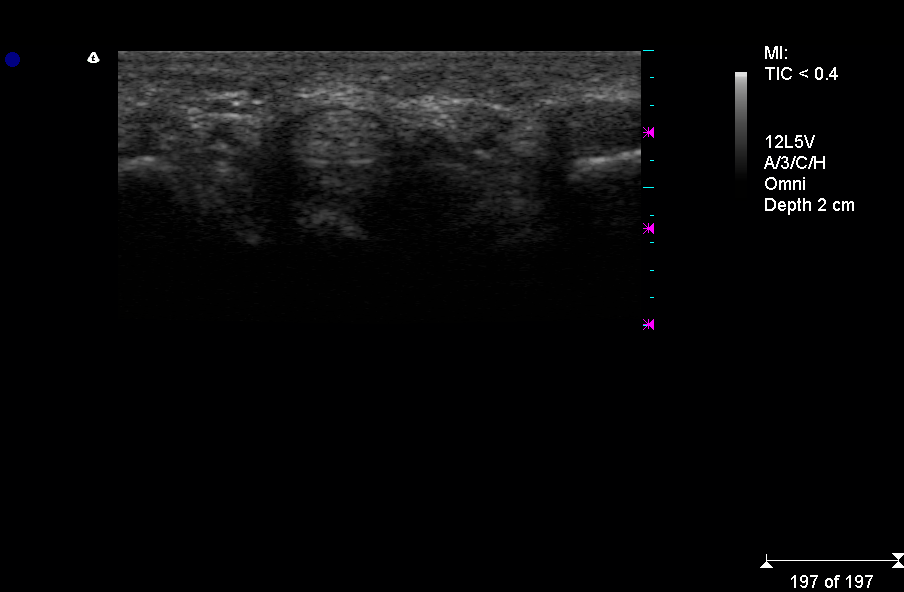

Supplement: S3 Dataset — (ZIP) [file pone.0187042.s003.zip › Classification Data/Normal/Right Hand/MD_1_R.bmp]

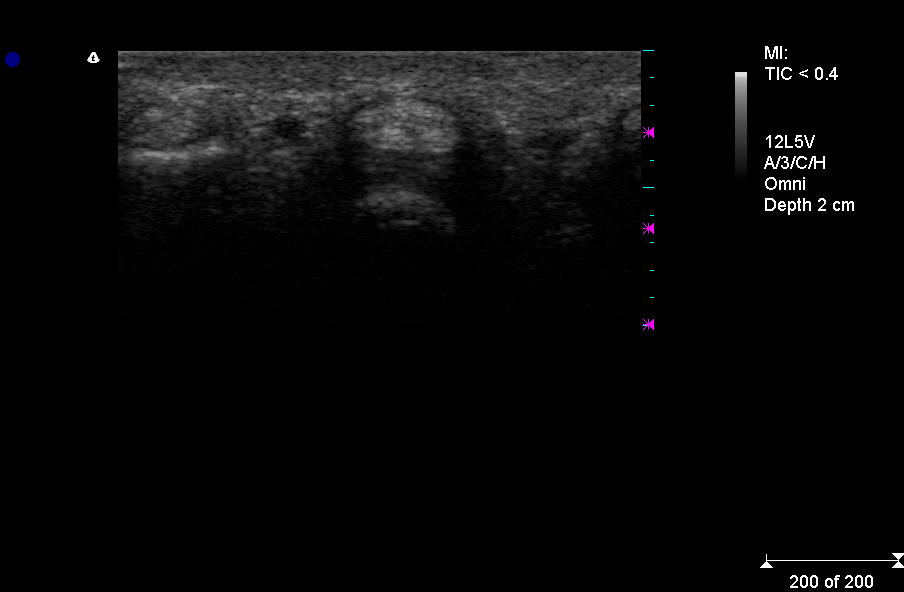

Supplement: S3 Dataset — (ZIP) [file pone.0187042.s003.zip › Classification Data/Normal/Right Hand/MD_2_R.bmp]

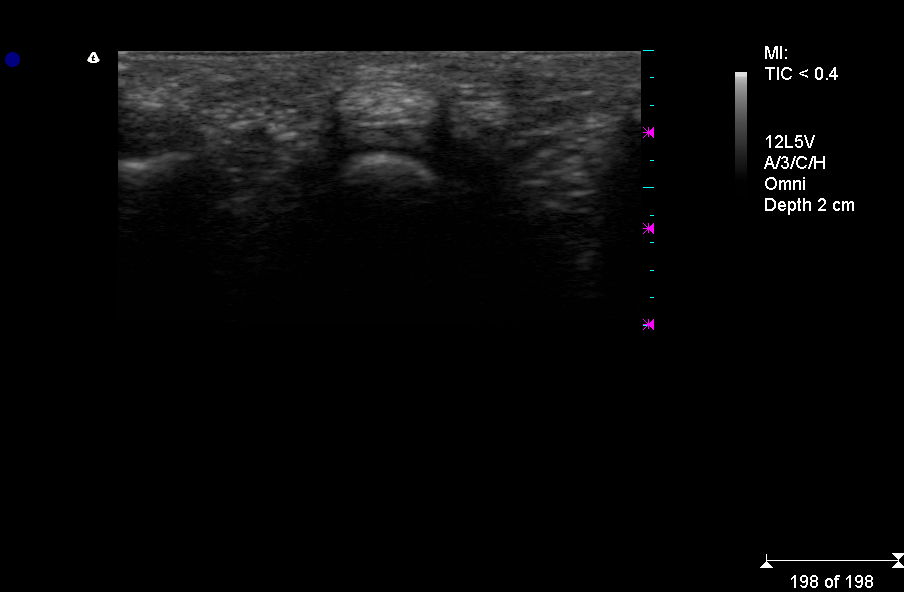

Supplement: S3 Dataset — (ZIP) [file pone.0187042.s003.zip › Classification Data/Normal/Right Hand/MD_3_R.bmp]

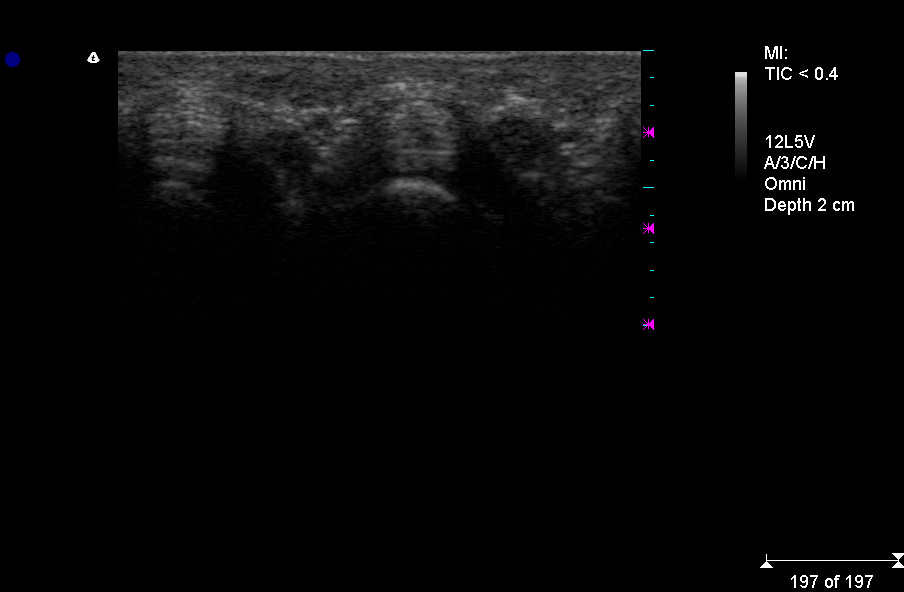

Supplement: S3 Dataset — (ZIP) [file pone.0187042.s003.zip › Classification Data/Normal/Right Hand/MD_4_R.bmp]

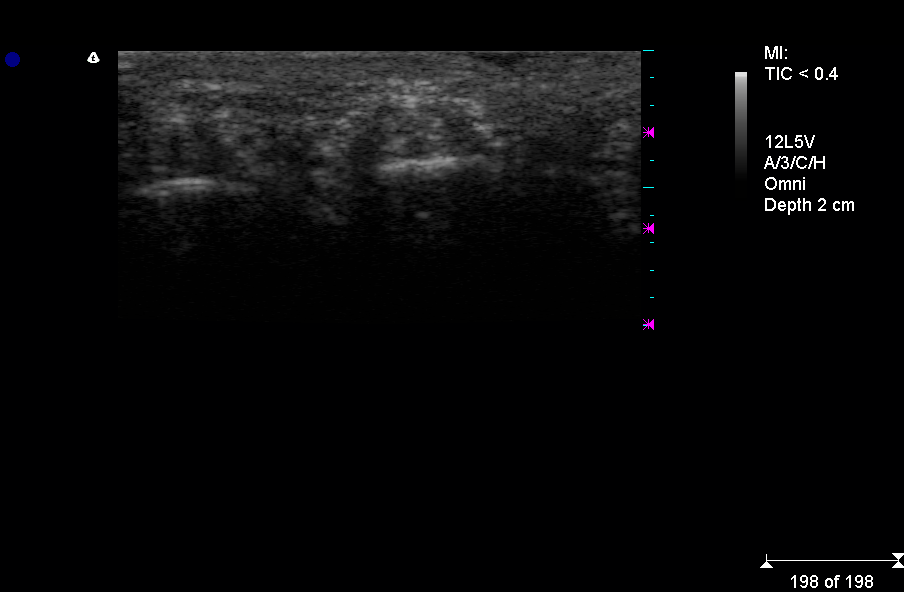

Supplement: S3 Dataset — (ZIP) [file pone.0187042.s003.zip › Classification Data/Normal/Right Hand/MD_5_R.bmp]

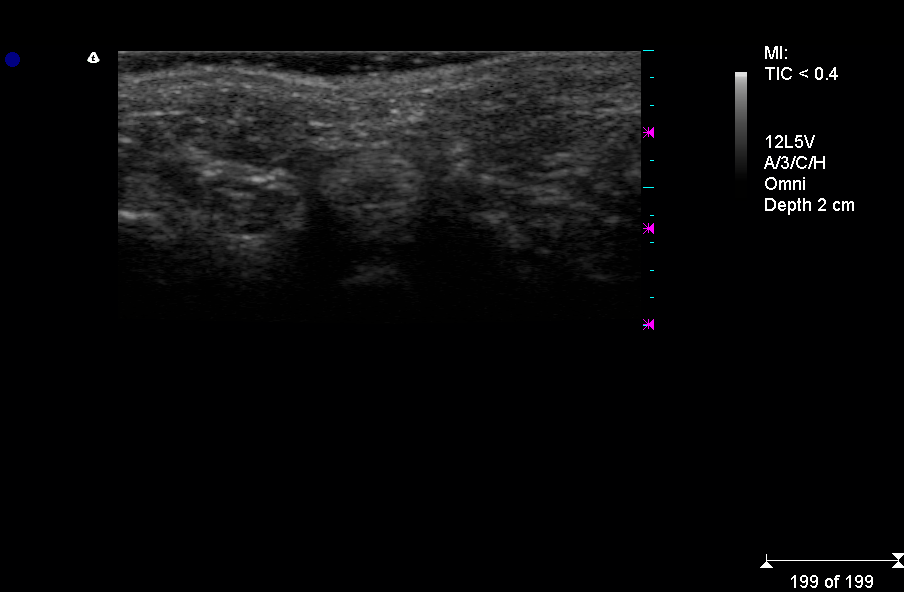

Supplement: S3 Dataset — (ZIP) [file pone.0187042.s003.zip › Classification Data/Normal/Right Hand/MD_6_R.bmp]

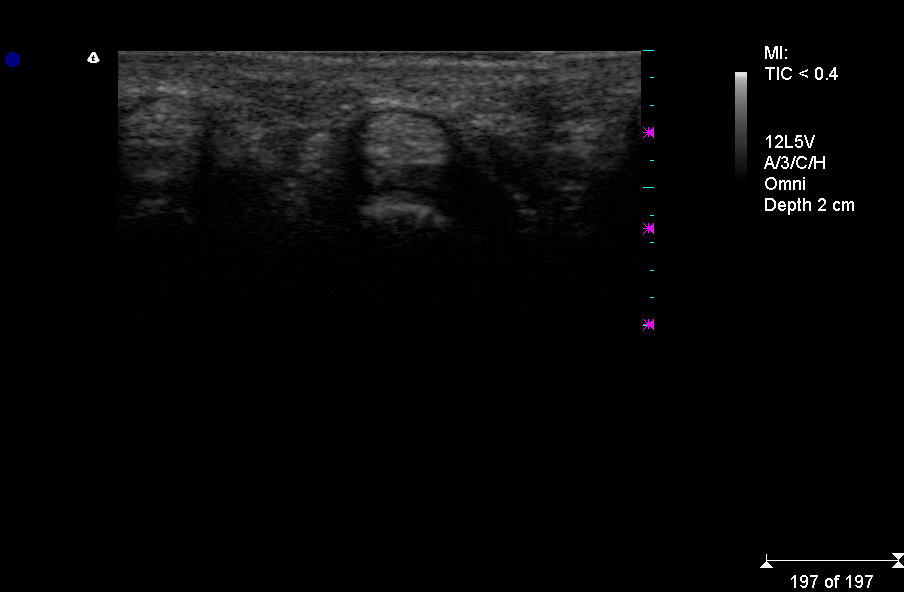

Supplement: S3 Dataset — (ZIP) [file pone.0187042.s003.zip › Classification Data/Normal/Right Hand/MD_7_R.bmp]

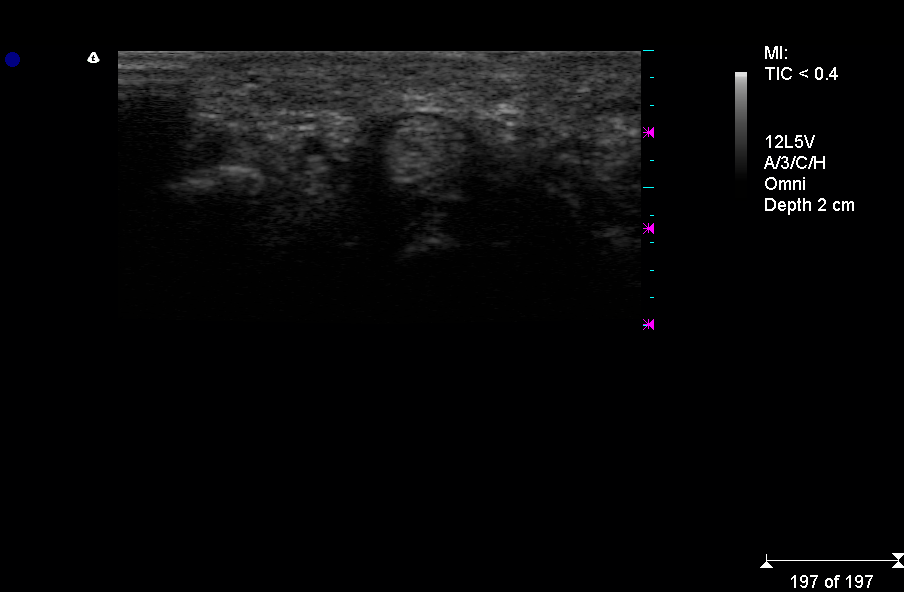

Supplement: S3 Dataset — (ZIP) [file pone.0187042.s003.zip › Classification Data/Normal/Right Hand/MD_8_R.bmp]

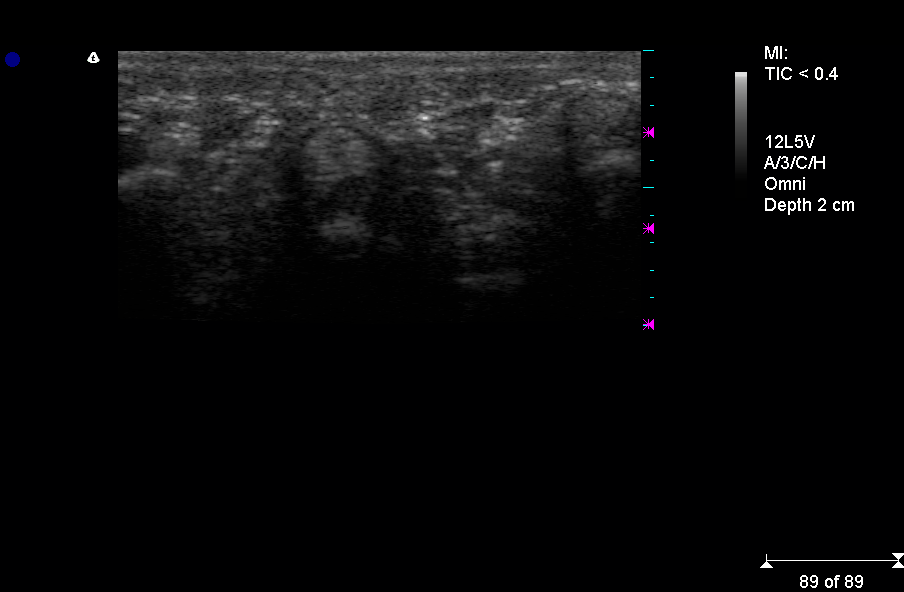

Supplement: S3 Dataset — (ZIP) [file pone.0187042.s003.zip › Classification Data/Normal/Right Hand/MD_9_R.bmp]

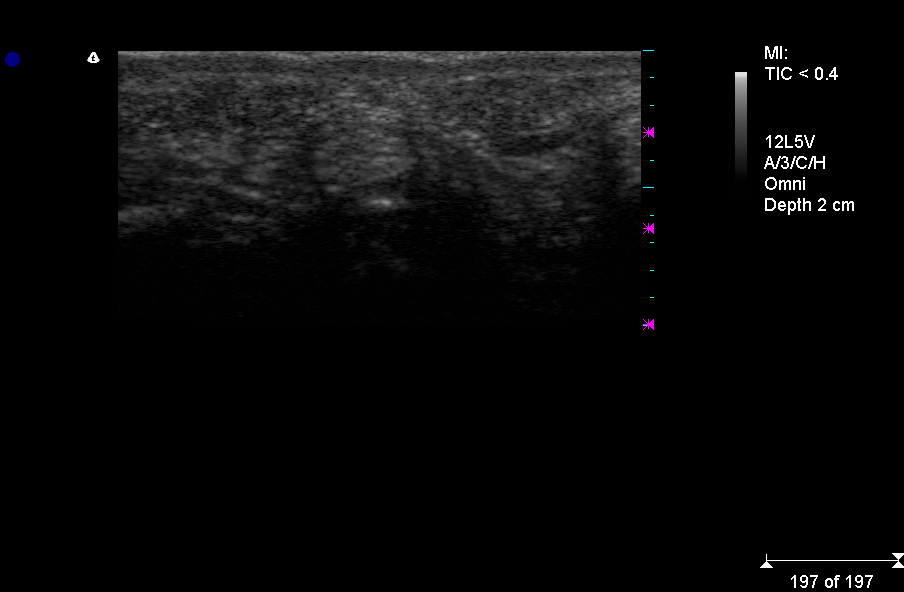

Supplement: S3 Dataset — (ZIP) [file pone.0187042.s003.zip › Classification Data/Normal/Right Hand/PD_1_R.bmp]

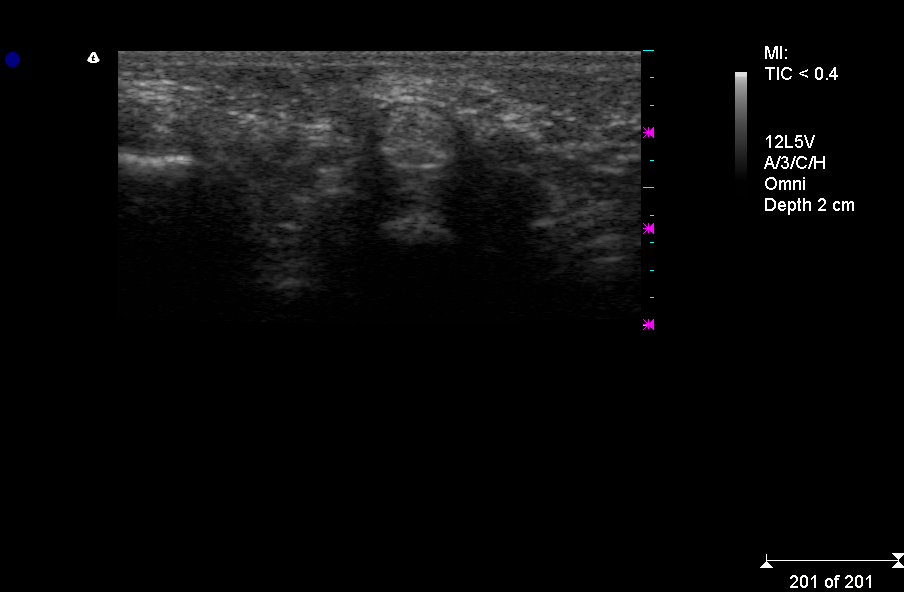

Supplement: S3 Dataset — (ZIP) [file pone.0187042.s003.zip › Classification Data/Normal/Right Hand/phD_1_R.bmp]

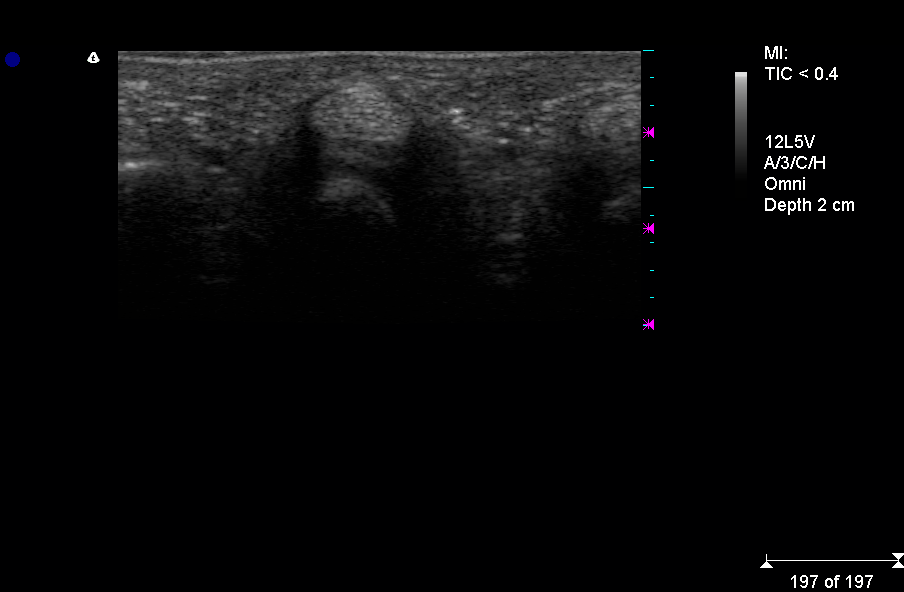

Supplement: S3 Dataset — (ZIP) [file pone.0187042.s003.zip › Classification Data/Normal/Right Hand/phD_2_R.bmp]

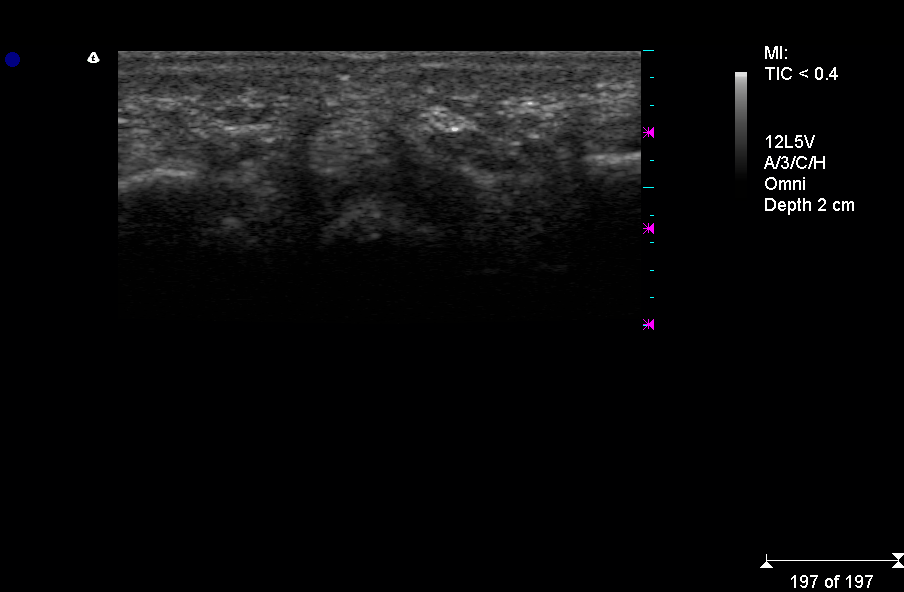

Supplement: S3 Dataset — (ZIP) [file pone.0187042.s003.zip › Classification Data/Normal/Right Hand/phD_3_R.bmp]

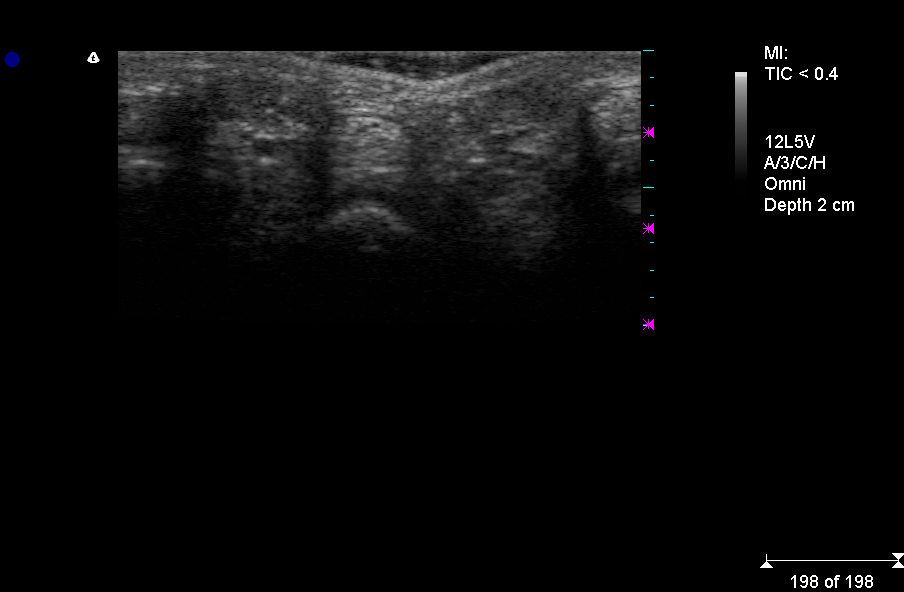

Supplement: S3 Dataset — (ZIP) [file pone.0187042.s003.zip › Classification Data/Normal/Right Hand/phD_4_R.bmp]

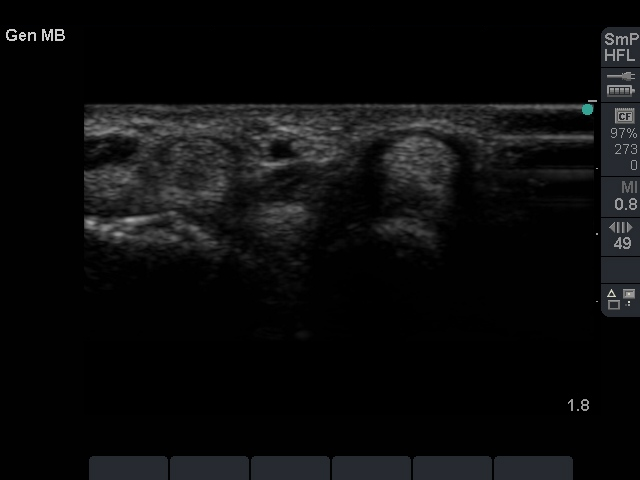

Supplement: S3 Dataset — (ZIP) [file pone.0187042.s003.zip › Classification Data/Patient/Normal Hand/108_R4T.bmp]

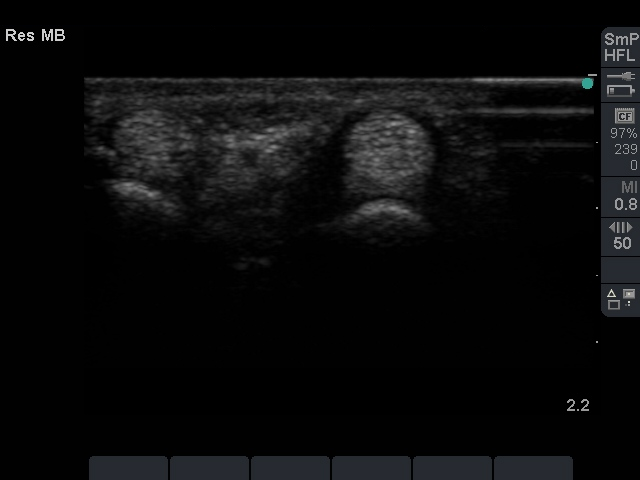

Supplement: S3 Dataset — (ZIP) [file pone.0187042.s003.zip › Classification Data/Patient/Normal Hand/113_R3T.bmp]

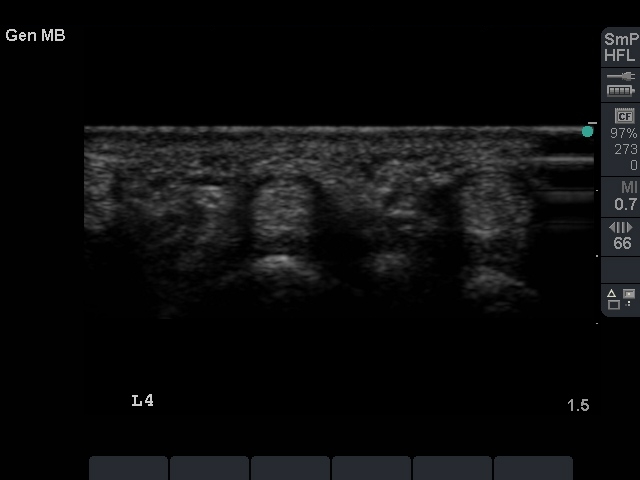

Supplement: S3 Dataset — (ZIP) [file pone.0187042.s003.zip › Classification Data/Patient/Normal Hand/115_L4T.bmp]

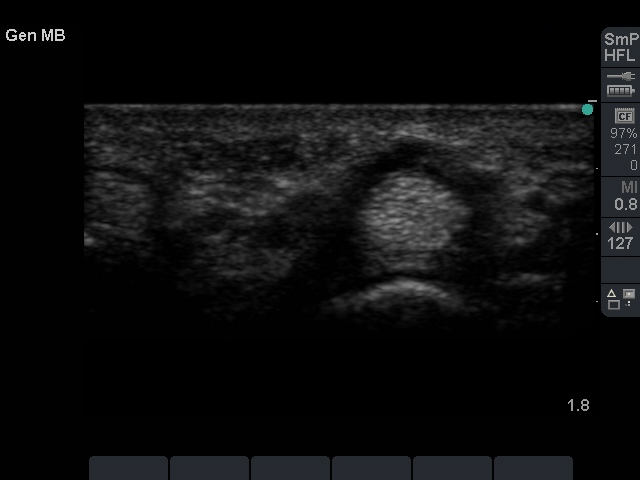

Supplement: S3 Dataset — (ZIP) [file pone.0187042.s003.zip › Classification Data/Patient/Normal Hand/128_R3T.bmp]

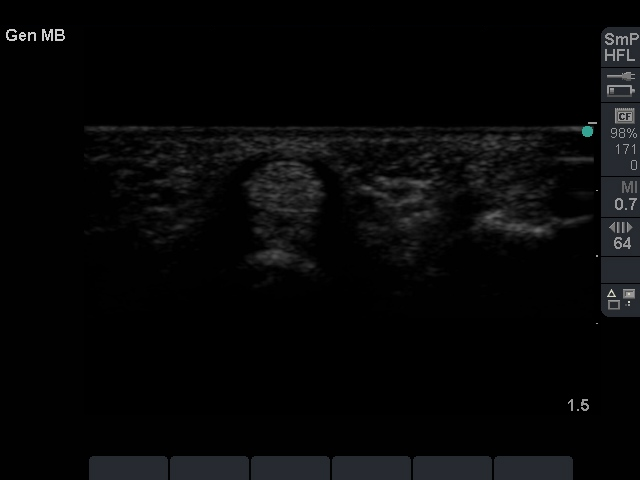

Supplement: S3 Dataset — (ZIP) [file pone.0187042.s003.zip › Classification Data/Patient/Normal Hand/129_L3T.bmp]

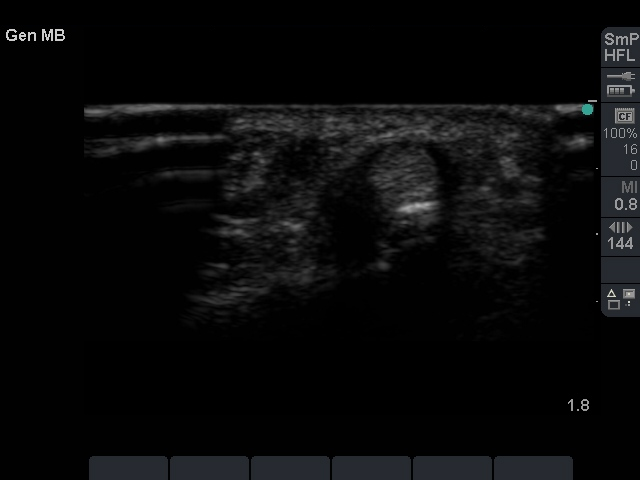

Supplement: S3 Dataset — (ZIP) [file pone.0187042.s003.zip › Classification Data/Patient/Normal Hand/131_R1T.bmp]

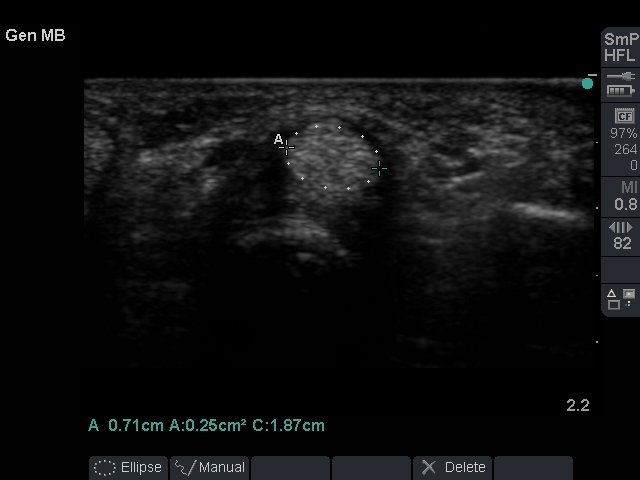

Supplement: S3 Dataset — (ZIP) [file pone.0187042.s003.zip › Classification Data/Patient/Normal Hand/134_R3T.bmp]

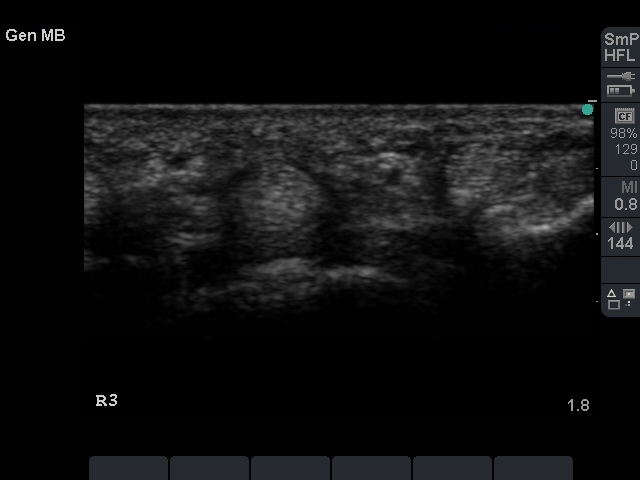

Supplement: S3 Dataset — (ZIP) [file pone.0187042.s003.zip › Classification Data/Patient/Normal Hand/136_R3T.bmp]

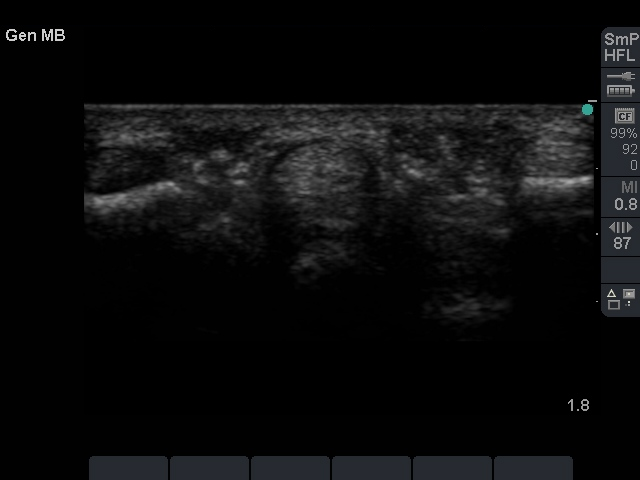

Supplement: S3 Dataset — (ZIP) [file pone.0187042.s003.zip › Classification Data/Patient/Normal Hand/139_L3T.bmp]

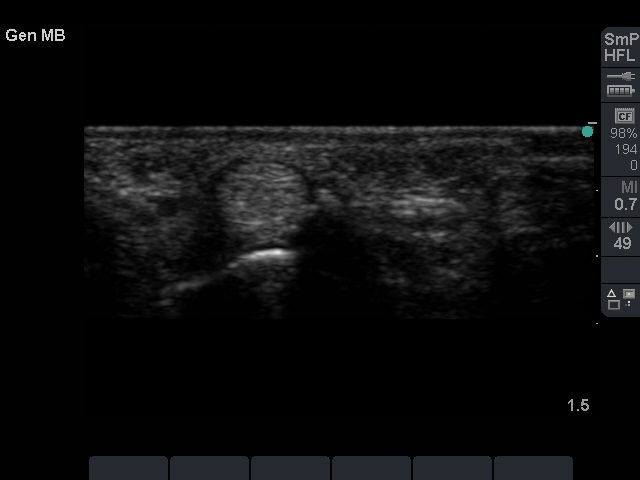

Supplement: S3 Dataset — (ZIP) [file pone.0187042.s003.zip › Classification Data/Patient/Normal Hand/149_L3T.bmp]

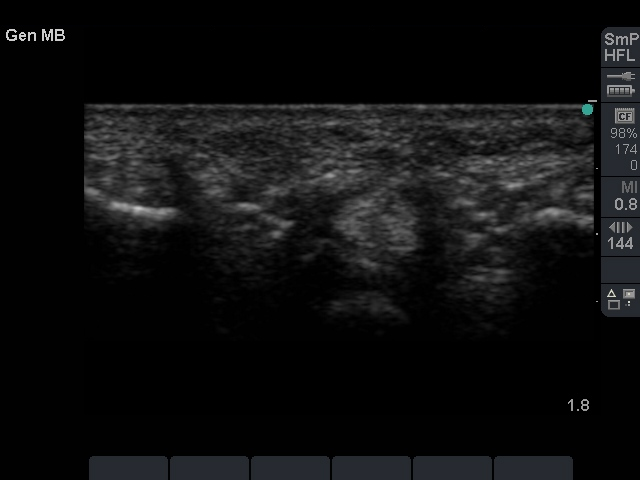

Supplement: S3 Dataset — (ZIP) [file pone.0187042.s003.zip › Classification Data/Patient/Normal Hand/156_R3T.bmp]

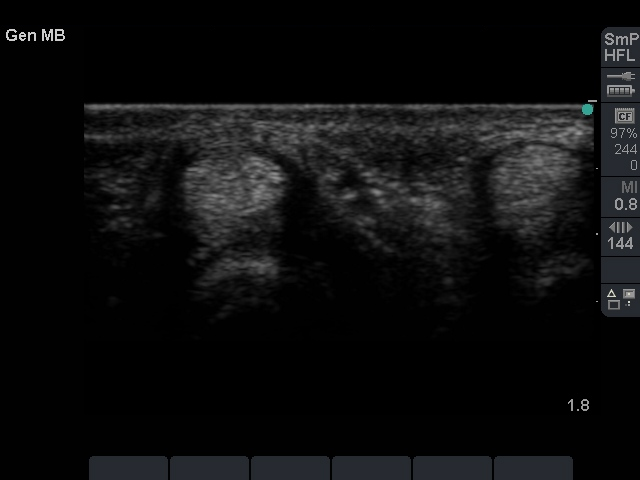

Supplement: S3 Dataset — (ZIP) [file pone.0187042.s003.zip › Classification Data/Patient/Normal Hand/159_R3T.bmp]

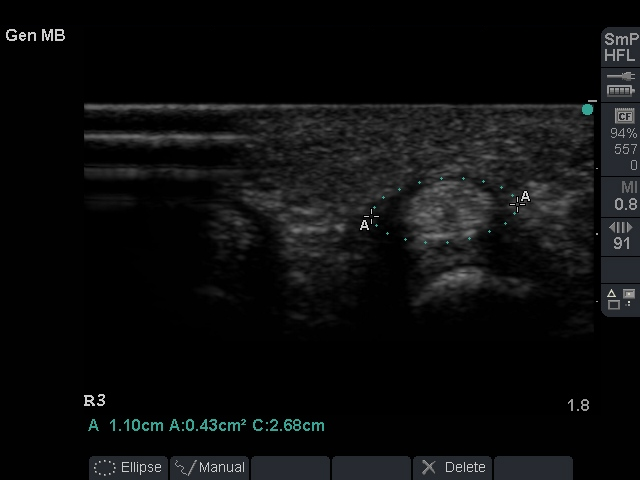

Supplement: S3 Dataset — (ZIP) [file pone.0187042.s003.zip › Classification Data/Patient/Normal Hand/185_R3T.bmp]

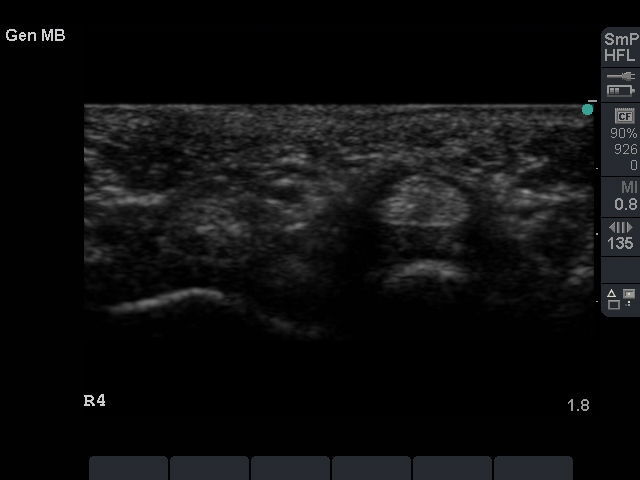

Supplement: S3 Dataset — (ZIP) [file pone.0187042.s003.zip › Classification Data/Patient/Normal Hand/190_R4T.bmp]

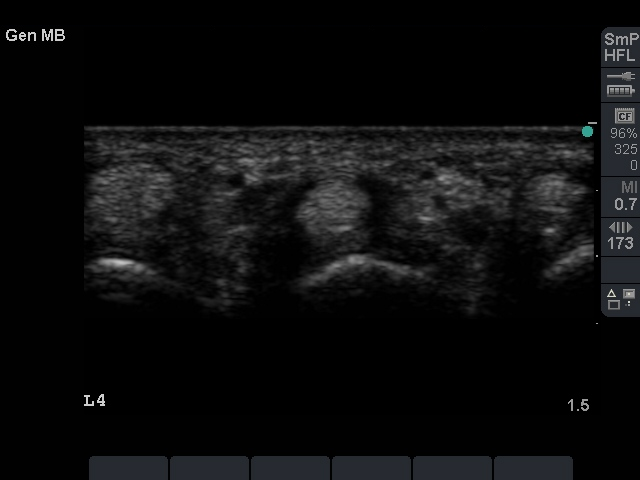

Supplement: S3 Dataset — (ZIP) [file pone.0187042.s003.zip › Classification Data/Patient/Normal Hand/193_L4T.bmp]

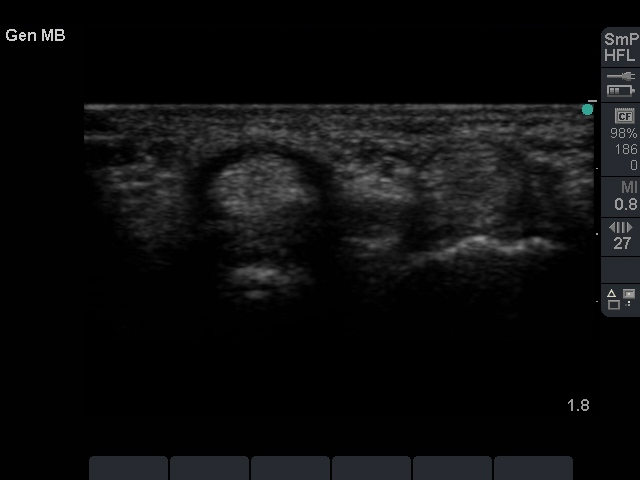

Supplement: S3 Dataset — (ZIP) [file pone.0187042.s003.zip › Classification Data/Patient/Normal Hand/251_R4T.bmp]

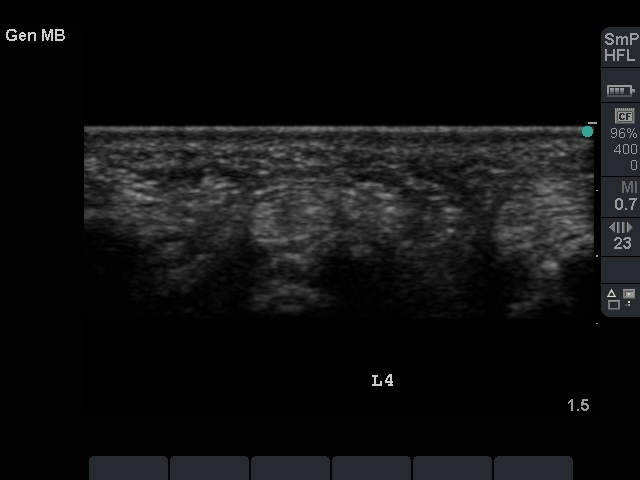

Supplement: S3 Dataset — (ZIP) [file pone.0187042.s003.zip › Classification Data/Patient/Normal Hand/256_L4T.bmp]

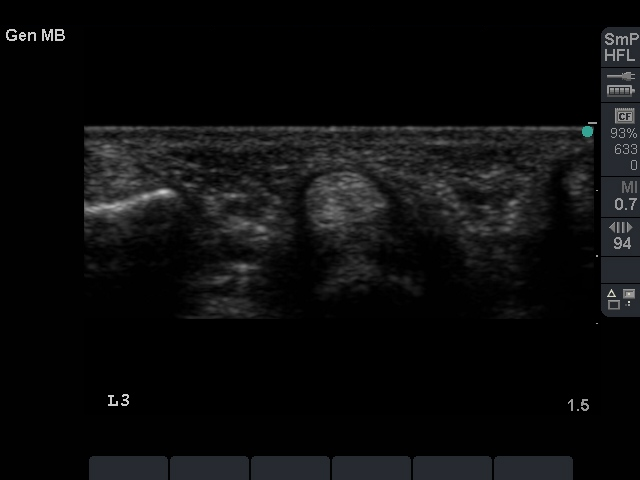

Supplement: S3 Dataset — (ZIP) [file pone.0187042.s003.zip › Classification Data/Patient/Normal Hand/258_L3T.bmp]

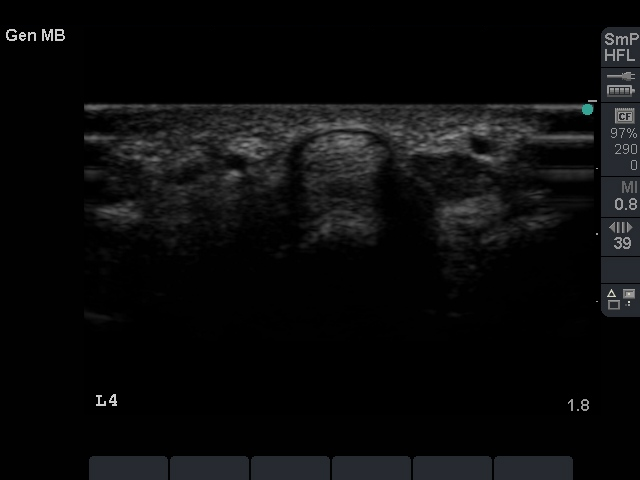

Supplement: S3 Dataset — (ZIP) [file pone.0187042.s003.zip › Classification Data/Patient/Trigger Finger/108_l4tnil.bmp]

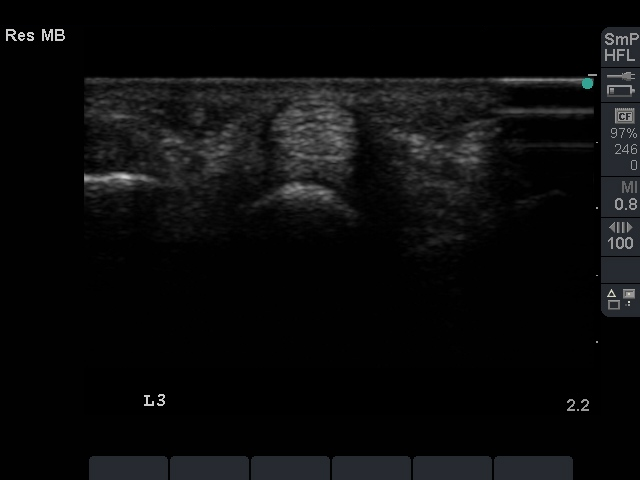

Supplement: S3 Dataset — (ZIP) [file pone.0187042.s003.zip › Classification Data/Patient/Trigger Finger/113_l3t.bmp]

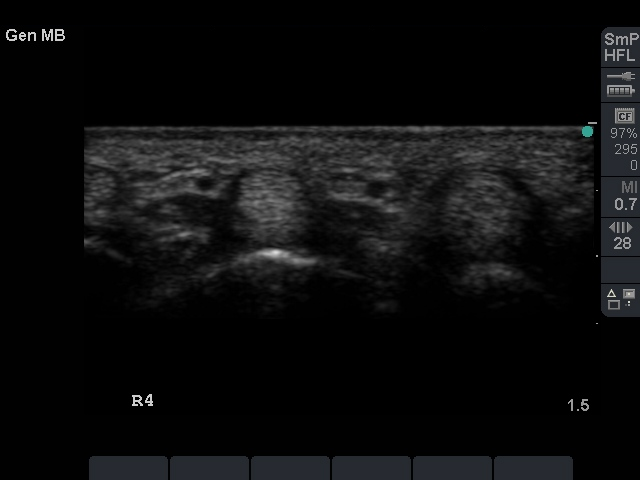

Supplement: S3 Dataset — (ZIP) [file pone.0187042.s003.zip › Classification Data/Patient/Trigger Finger/115_R4T.bmp]

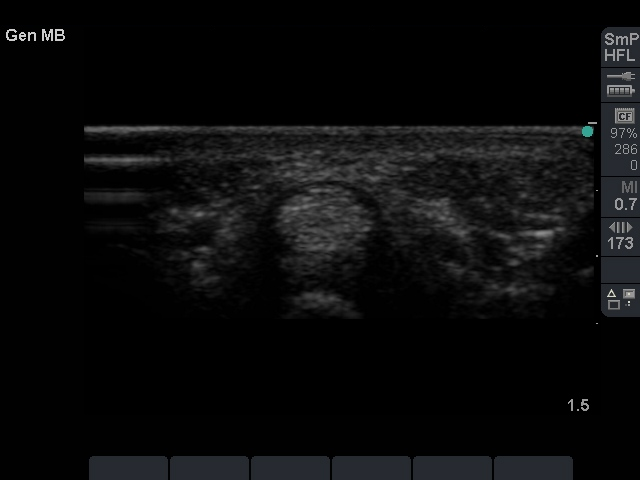

Supplement: S3 Dataset — (ZIP) [file pone.0187042.s003.zip › Classification Data/Patient/Trigger Finger/128_L3T.bmp]

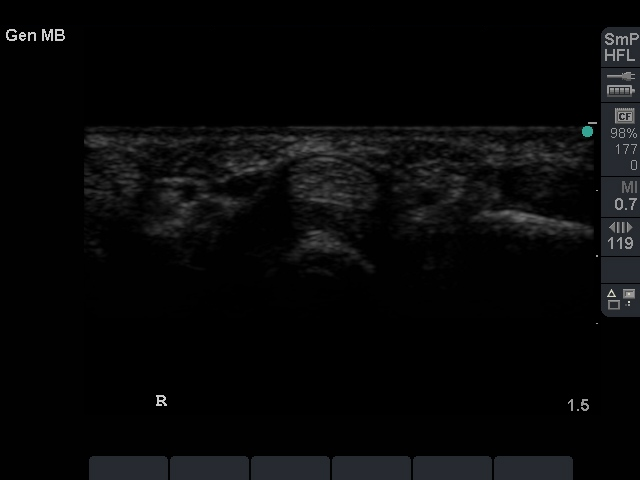

Supplement: S3 Dataset — (ZIP) [file pone.0187042.s003.zip › Classification Data/Patient/Trigger Finger/129_R3T.bmp]

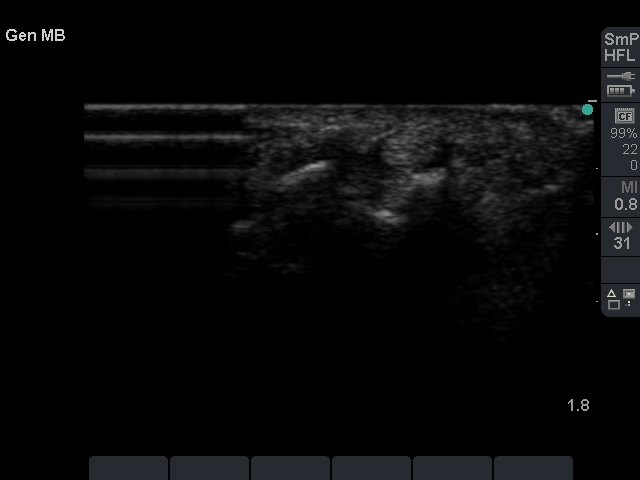

Supplement: S3 Dataset — (ZIP) [file pone.0187042.s003.zip › Classification Data/Patient/Trigger Finger/131_L1T.bmp]

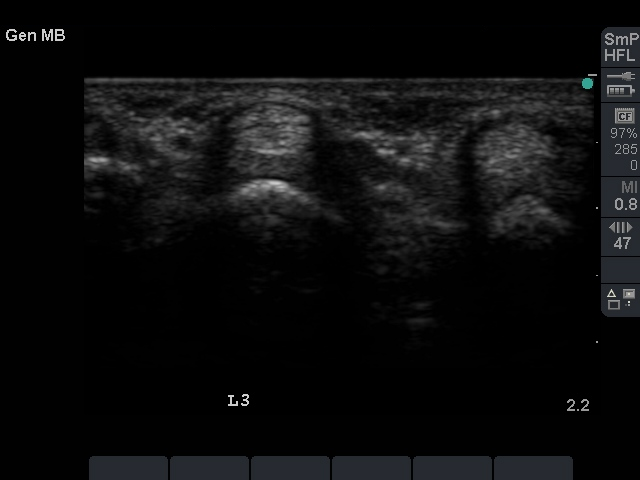

Supplement: S3 Dataset — (ZIP) [file pone.0187042.s003.zip › Classification Data/Patient/Trigger Finger/134_L3T.bmp]

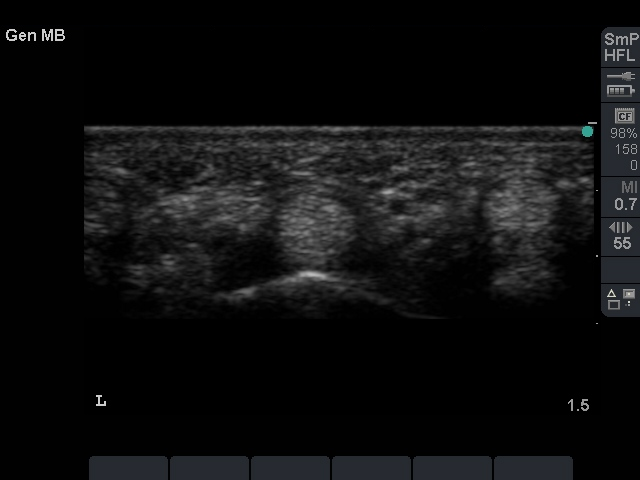

Supplement: S3 Dataset — (ZIP) [file pone.0187042.s003.zip › Classification Data/Patient/Trigger Finger/136_L3T3.bmp]

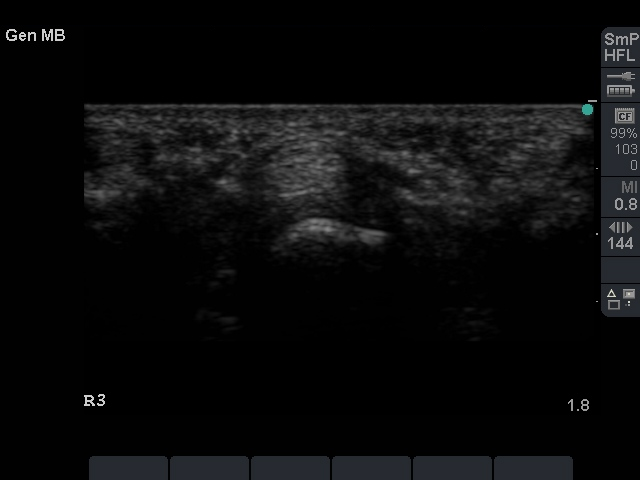

Supplement: S3 Dataset — (ZIP) [file pone.0187042.s003.zip › Classification Data/Patient/Trigger Finger/139_R3T.bmp]

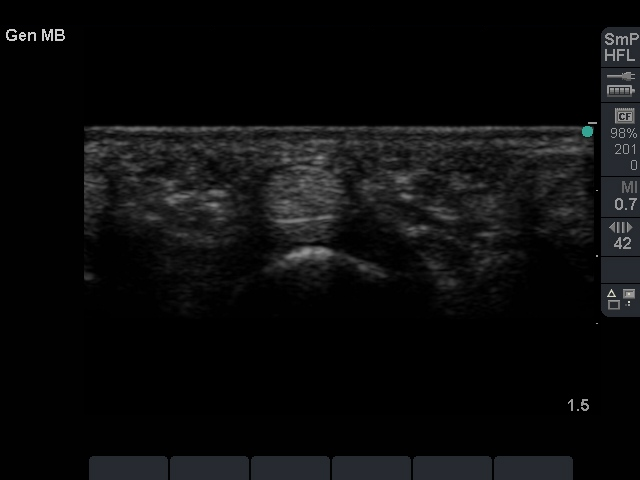

Supplement: S3 Dataset — (ZIP) [file pone.0187042.s003.zip › Classification Data/Patient/Trigger Finger/149_R3T.bmp]

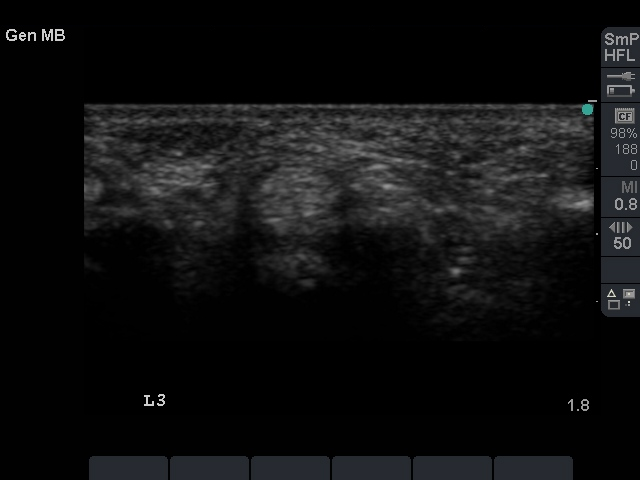

Supplement: S3 Dataset — (ZIP) [file pone.0187042.s003.zip › Classification Data/Patient/Trigger Finger/156_L3T.bmp]

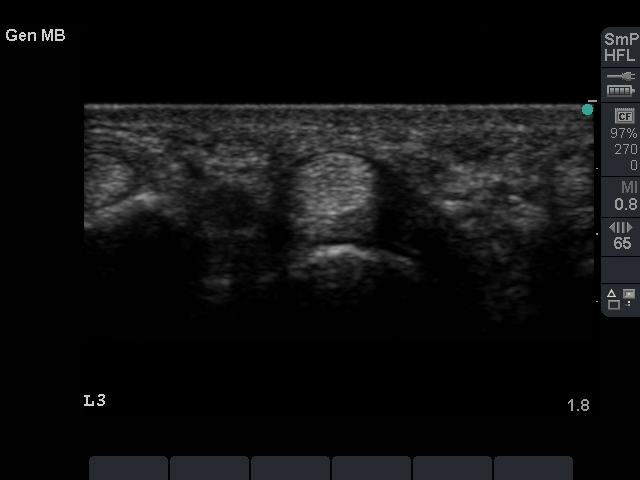

Supplement: S3 Dataset — (ZIP) [file pone.0187042.s003.zip › Classification Data/Patient/Trigger Finger/159_L3T.bmp]

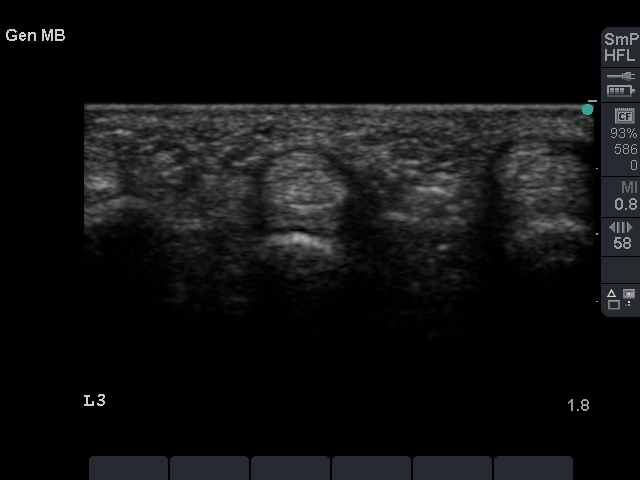

Supplement: S3 Dataset — (ZIP) [file pone.0187042.s003.zip › Classification Data/Patient/Trigger Finger/185_L3T.bmp]

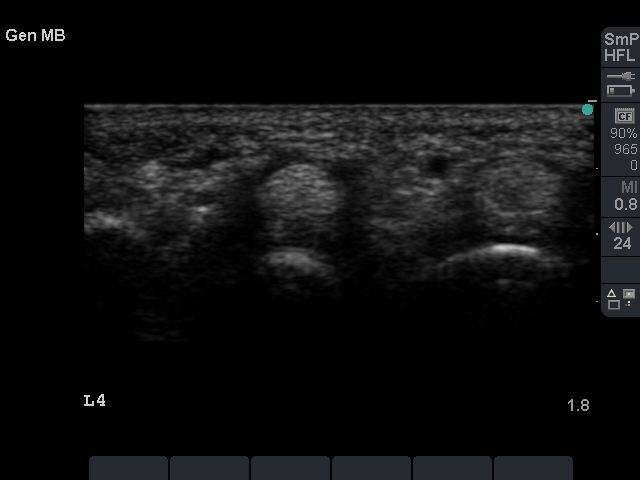

Supplement: S3 Dataset — (ZIP) [file pone.0187042.s003.zip › Classification Data/Patient/Trigger Finger/190_L4T.bmp]

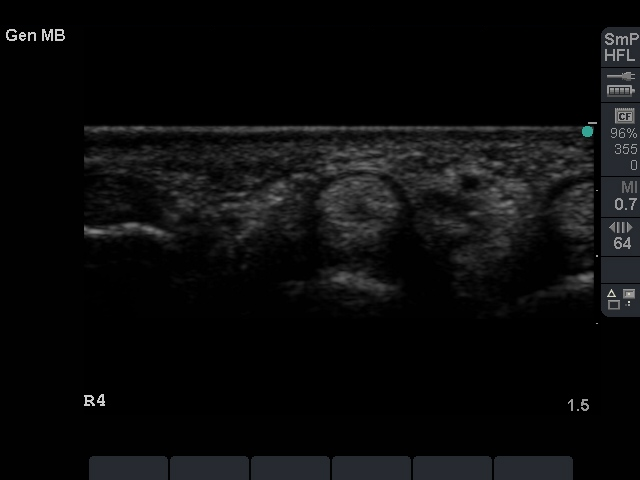

Supplement: S3 Dataset — (ZIP) [file pone.0187042.s003.zip › Classification Data/Patient/Trigger Finger/193_R4T.bmp]

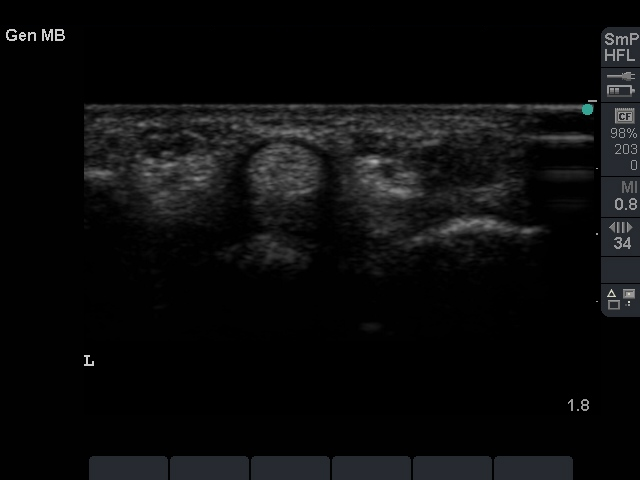

Supplement: S3 Dataset — (ZIP) [file pone.0187042.s003.zip › Classification Data/Patient/Trigger Finger/251_L4T.bmp]

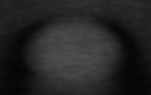

Supplement: S1 File — (ZIP) [file pone.0187042.s004.zip › ATASM_public/Param/sheath/SheathTemplate.bmp]

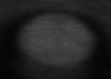

Supplement: S1 File — (ZIP) [file pone.0187042.s004.zip › ATASM_public/Param/tendon/clearboundary/TendonTemplate.bmp]

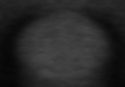

Supplement: S1 File — (ZIP) [file pone.0187042.s004.zip › ATASM_public/Param/tendon/fuzzyboundary/TendonTemplate.bmp]
